# Musculoskeletal networks reveal topological disparity in mammalian neck evolution

**DOI:** 10.1186/s12862-017-1101-1

**Published:** 2017-12-13

**Authors:** Patrick Arnold, Borja Esteve-Altava, Martin S. Fischer

**Affiliations:** 1 Department of Human Evolution, Max Planck Institute for Evolutionary Anthropology, Leipzig, Germany; 2 Institut für Spezielle Zoologie und Evolutionsbiologie mit Phyletischem Museum, Friedrich-Schiller-Universität Jena, Jena, Germany; 3 Structure & Motion Lab, Department of Comparative Biomedical Sciences, Royal Veterinary College, Hatfield, UK

**Keywords:** Anatomical network analysis, Network theory, Forelimb evolution, Mammalian cervical spine, Sloths, Meristic constraints, Modularity

## Abstract

**Background:**

The increase in locomotor and metabolic performance during mammalian evolution was accompanied by the limitation of the number of cervical vertebrae to only seven. In turn, nuchal muscles underwent a reorganization while forelimb muscles expanded into the neck region. As variation in the cervical spine is low, the variation in the arrangement of the neck muscles and their attachment sites (i.e., the variability of the neck’s musculoskeletal organization) is thus proposed to be an important source of neck disparity across mammals. Anatomical network analysis provides a novel framework to study the organization of the anatomical arrangement, or connectivity pattern, of the bones and muscles that constitute the mammalian neck in an evolutionary context.

**Results:**

Neck organization in mammals is characterized by a combination of conserved and highly variable network properties. We uncovered a conserved regionalization of the musculoskeletal organization of the neck into upper, mid and lower cervical modules. In contrast, there is a varying degree of complexity or specialization and of the integration of the pectoral elements. The musculoskeletal organization of the monotreme neck is distinctively different from that of therian mammals.

**Conclusions:**

Our findings reveal that the limited number of vertebrae in the mammalian neck does not result in a low musculoskeletal disparity when examined in an evolutionary context. However, this disparity evolved late in mammalian history in parallel with the radiation of certain lineages (e.g., cetartiodactyls, xenarthrans). Disparity is further facilitated by the enhanced incorporation of forelimb muscles into the neck and their variability in attachment sites.

**Electronic supplementary material:**

The online version of this article (10.1186/s12862-017-1101-1) contains supplementary material, which is available to authorized users.

## Background

The increase in locomotor and metabolic performance was one of the most important innovations in the evolution of mammals [1–6]. This innovation, however, was accompanied by an exceptionally low variability in the number of presacral vertebrae compared to other tetrapods (e.g., [7–14]). In fact, the number of cervical vertebrae in mammals is limited to seven, except in extant manatees and sloths [12]. As mammals evolved a new locomotor mode based on an increase in sagittal axial motions, their back and nuchal muscles underwent an anatomical reorganization [15, 16]. The epaxonic muscles (particularly the iliocostalis system) were reduced along with the decrease of lateral axial motion [15, 16]. With the predominance of girdle-limb system as the main propeller in mammals, pectoral muscles also expanded into the dorsal region [15] and were integrated into the head/neck functional unit. Studies on the evolution of the mammalian neck usually have focused on the role of those muscles emigrating from the cervical region during early development [17–20]. In contrast, the muscles that expanded into the neck have been solely investigated for their impact on shoulder and forelimb mechanics (e.g., [21–24]).

Differences in ecology and size resulted in interspecific differences in the posture and mobility of the head in mammals during standing, locomotion, foraging, oral grooming, and other daily activities (e.g., [25–30]). The morphological basis of these differences, however, is poorly understood. Variation of the cervical column length as a whole has recently been shown to be an important factor in generating morphological disparity of the neck in mammals [31]. As a consequence of the limited variability in the number of vertebrae [9, 12] and in vertebral shape [17, 32–35], the disparity of the cervical skeleton alone is still low. Hence, we suggest that interspecific variation in the arrangement of the neck muscles plus their attachment sites on the cervical vertebrae, the skull and other bones (i.e., the variability of the musculoskeletal organization) should be an important source of morphological disparity of the neck across mammals. Although there are numerous descriptions of the myology of the neck region for almost every mammalian family, only a few studies compared the neck muscle arrangement interspecifically in an evolutionary context (e.g., [36–39]). Moreover, these studies compared neck muscles only qualitatively, which prevents the quantification of the differentiation of the neck muscles arrangement. As a consequence, it is currently unknown whether the interspecific variation in muscle attachments actually affected the changes of the musculoskeletal organization of the neck across mammals.

Anatomical Network Analysis (AnNA) provides a novel framework to study the organization of the anatomical arrangement of bones and muscles of anatomical structures (i.e., the connectivity pattern) [40, 41]. Within this framework, bones and muscles are formalized as the nodes of the network, and the physical contacts among them are formalized as the links that connect the network’s nodes. Anatomical network models thus offer a mathematical description of the organization of the body [41]. Through such mathematical formalism we can identify and quantify structural patterns, such as anatomical modules, without a priori assumptions about the developmental or functional factors causing them [40] (for a recent review on morphological modularity, see [42]). This allows direct phylogenetic comparisons. In this context, AnNA has been used, for example, to infer evolutionary trends in the skull of tetrapods [43] and the phylogenetic relation between morphological complexity and modularity in the skull of primates [44]. AnNA formalization also allows combining information on skeletal and muscular tissues as it was done to study congenital musculoskeletal malformations [45], secondary injuries [46] in humans, and hindlimb functional integration in frogs [47].

We can also use network parameters as proxies to infer the morphological organization of the body. Table 1 summarizes the network parameters used in this study and their most common morphological interpretation. Further details on the interpretation of network concepts in a morphological context and their historical roots have been given elsewhere (see, e.g., [41, 43, 48–50]). In short, every interpretation derives from the biological role of connections in the network model. Broadly speaking, the connections we have modeled among the bones and muscles of the neck embody functional interactions (e.g., determining the range of neck motion), as well as developmental factors (e.g., those inducing muscles to attach to a specific vertebrae and not to other). Thus, for example, the number of such connections for a given element, or for the entire network (i.e., K), represents the amount of functional and developmental dependences of this element or of the whole network. Functional and developmental dependences are often associated to Rupert Rield’s concept of burden, or more generally, to the concept of constrain of body parts [40, 51–53]. Morphological interpretation of more elaborated network parameters, such as the density of connections (D), the mean clustering coefficient (C), and the mean shortest path length (L), follow a similar logic. Because connections represent biological interactions among anatomical parts, their relative amount (D) serves as a proxy of the complexity available to the system (e.g., to perform complex functions). In addition to quantifying the amount of connections, the way connections are set (e.g., creating intertwined patterns such as 3-node loops (C)) and their effects on topology (e.g., increasing the effective proximity of elements to interact together (L)) also have consequences in the overall integration of the anatomy. Thus, the greater the intertwining, the greater the integration; the closer the elements, the greater the integration. Moreover, differences in the amount of connections among the elements of the network (some have many, most have a few) introduces heterogeneity in the organization of the network. Such heterogeneity can be related before to structural disparity (or anisomerism sensu Gregory [43, 54]). Finally, the overall patterns of integration and heterogeneity among the parts of a network often results in the emergence of new properties, for example, modularity [48] (see [55–58] for general reviews on the origin and macroevolution of modularity at a morphological level). The more specific details of the modular organization of a network need a closer observation, but the overall degree of parcellation of the network into large, uniform modules (P) captures how much modular the neck is.

**Table 1:** Summary of network parameters used in this study

Network parameter	Mathematical definition	Morphological interpretation
Number of nodes (N)	Direct count of the number of nodes in the network	Number of anatomical elements
Number of links (K)	Direct count of the number of links in the network	Number of anatomical relations (burden or constrain), connectivity
Density of connections (D)	Relative amount of links: $D=\frac{2K}{N\left(N-1\right)}$	Morphological complexity
Mean clustering coefficient (C)	Relative amount of 3-node loops: $C=\frac{1}{N}\sum \frac{\sum {loops}_i}{k_i\left({k}_i-1\right)}$ where *e*_*i*_ is the existing number of links among the neighbors of node *i* and *k*_*i*_ is the total number of links of a node *i*	Co-dependency (integration)
Mean shortest path length (L)	Average distance between every pair of nodes: $L=\frac{1}{N-1}\sum {d}_{n_i,{n}_j}$ where *d* is the shortest distance in number of links between a given pair of nodes *n*_*i*_ and *n*_*j*_	Effective proximity (integration)
Heterogeneity of connections (H)	Disparity in the number of links per node: *H* = *σ*_*K*_/*μ*_*K*_ where *σ*_*K*_ and *μ*_*K*_ are the standard deviation and mean of *K*, respectively	Anisomerism
Parcellation (P)	Extent and uniformity of the modular division: *P* = 1 − ∑ (*N*_*m*_/*N*)^2^ where *N*_*m*_ is the number of nodes in module *m*	Degree of modularity

**Fig. 1:**
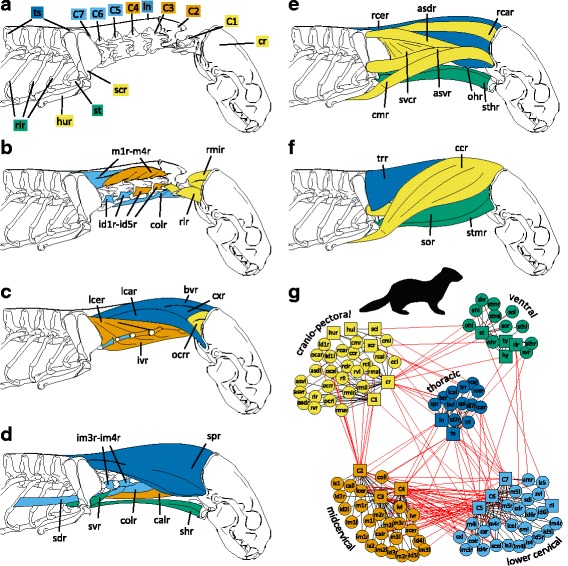
Musculoskeletal anatomy of the mammalian neck and its translation into the anatomical network. **a** Skeletal and (**b**-**f**) muscular elements of the neck included in the analysis (from deep to superficial) exemplified for the lesser grison (*Galictis cuja*). **g** Anatomical network representing the same topological information. Colors code for the identified connectivity modules. Red links between-modules. Black links within modules. **a**-**f** adopted and modified after [138, 139]

Here we applied AnNA to a phylogenetically broad dataset of mammalian necks, including all bones (cervical vertebrae, cranium, sternum, hyoid and pectoral girdle) and muscles involved in the motion of the head and neck (Fig. 1a-f). First, we modeled the neck of each species as a network in which nodes represented the aforementioned bones and muscles and links represented their physical arrangement or contacts. Then, we quantified seven network parameters that serve as proxies for the morphological organization of the neck anatomy (Table 1). Finally, we explored the disparity of neck musculoskeletal organization through time to answer, specifically: 1) whether the musculoskeletal organization (as captured by network parameters) of the neck really differs among mammals; 2) whether closely related species share similar network organization; 3) whether there is a consistent pattern of modularity across mammalian necks; 4) how extreme elongation and deviating vertebral numbers alter neck organization; and 5) how disparity in neck anatomical organization changed during mammalian evolutionary history.

## Results

### Network parameters and phylogenetic signal

**Fig. 2:**
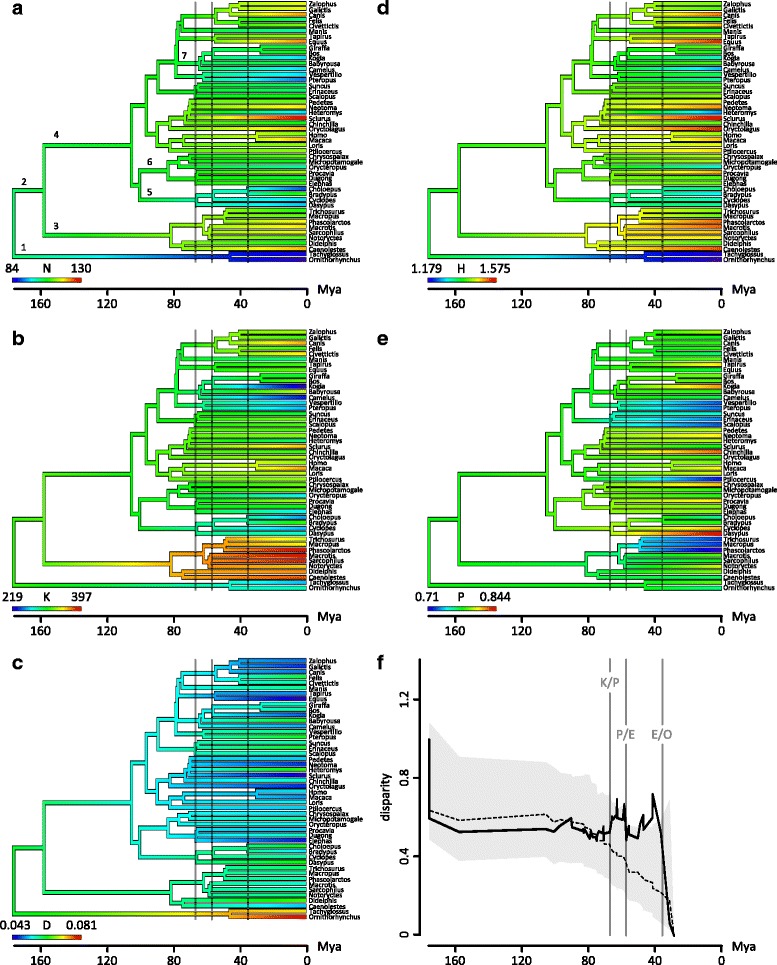
Phylogenetic signal and disparity through time (DTT) for neck network parameters across mammals. **a** Number of elements *N*; (**b**) Number of connections *K*; (**c**) Density of connections *D*; (**d**) Heterogeneity of connections *H*; (**e**) Parcellation index *P*; (**f**) mean subclade disparity through time plot. Gray vertical lines indicate Cretaceous-Tertiary (K/T) Paleocen-Eocene (P/E), and Eocene-Oligocene (E/O) boundary. Numbers indicate selected taxa: 1 Monotremata: 2 Theria; 3 Marsupialia; 4 Placentalia; 5 Xenarthra; 6 Afrotheria; 7 Cetartiodactyla. In (**f**). the solid line indicates actual median subclade DTT of the sample. The dashed line indicates the median subclade DTT based on 10,000 simulations of character evolution under Brownian motion. The shaded area indicates the 95% DTT range for the simulated data

The values of the network parameters used as proxies for the musculoskeletal organization of the neck for individual taxa are listed in Table 2. The phylogenetic signal is statistically significant for the multivariate dataset of all network parameters (Kmult = 0.89, *p* < 0.001) as well as for five of the seven parameters (*N*, *K*, *D*, *H*, *P*; Table 3, Additional file 1: AF1). This suggests similar variation in neck organization in closely related species (Fig. 2). A Brownian motion model of evolution best explains trait evolution of the network parameters (see Additional file 1: AF1). Relative variability in connectivity *K*, complexity *D*, and integration by co-dependency *C* is high among the species examined (significant higher coefficients of variation; Msrl = 139.58, *p* < 0.001) (Table 3, Additional file 1: AF1). In contrast, relative variability of integration by effective proximity *L*, anisomerism *H*, and the degree of modularity *P* are low across all mammals (Table 3, Additional file: AF1). There is no significant relationship between the network parameters and either body mass (F = 0.170, *p* = 0.68), absolute neck length (F = 0.005, *p* = 0.94), relative neck length (F = 0.661, *p* = 0.42), or predatory behavior (i.e., predatory vs. non-predatory; F = 0.191, p = 0.68).

**Table 2:** Network parameters of the musculoskeletal organization of the neck of 48 mammalian species

Order	Species	N	K	D	C	L	H	P
Afrosoricida	*Chrysospalax trevelyani*	112	328	0.053	0.456	2.81	1.448	0.820
	*Micropotamogale ruwenzorii*	108	329	0.057	0.482	2.771	1.449	0.784
Carnivora	*Canis lupus*	123	368	0.049	0.351	2.714	1.529	0.784
	*Civettictis civetta*	113	357	0.056	0.468	2.706	1.504	0.767
	*Felis silvestris*	103	320	0.061	0.452	2.65	1.401	0.784
	*Galictis cuja*	118	322	0.047	0.448	2.928	1.416	0.780
	*Zalophus californianus*	118	338	0.049	0.479	2.795	1.43	0.801
Cetartiodactyla	*Babyrousa babyrussa*	106	344	0.062	0.398	2.734	1.409	0.784
	*Bos taurus*	108	329	0.057	0.41	2.777	1.395	0.789
	*Camelus bactrianus*	96	232	0.051	0.295	2.903	1.268	0.771
	*Giraffa camelopardalis*	106	309	0.056	0.435	2.731	1.421	0.793
	*Kogia breviceps*	96	219	0.048	0.488	3.099	1.316	0.825
Chiroptera	*Pteropus vampyrus*	92	260	0.062	0.452	2.778	1.329	0.732
	*Vespertilio murinus*	96	264	0.058	0.476	2.821	1.358	0.729
Cingulata	*Dasypus novemcinctus*	95	279	0.062	0.524	2.769	1.331	0.844
Dasyuromorpha	*Sarcophilus harrisii*	118	390	0.056	0.449	2.714	1.502	0.779
Didelphimorphia	*Didelphis virginiana*	108	374	0.065	0.385	2.668	1.413	0.795
Diprotodontia	*Macropus rufus*	112	355	0.057	0.446	2.726	1.425	0.725
	*Phascolarctos cinereus*	119	397	0.057	0.429	2.699	1.538	0.710
	*Trichosurus vulpecula*	109	348	0.059	0.402	2.743	1.432	0.725
Eulipotyphla	*Erinaceus europaeus*	104	330	0.062	0.441	2.747	1.419	0.734
	*Scalopus aquaticus*	108	322	0.056	0.425	2.788	1.439	0.728
	*Suncus murinus*	104	304	0.057	0.411	2.835	1.418	0.753
Hyracoidea	*Procavia capensis*	115	340	0.052	0.451	2.84	1.507	0.815
Lagomorpha	*Oryctolagus cuniculus*	122	343	0.046	0.422	2.831	1.556	0.786
Monotremata	*Ornithorhynchus anatinus*	84	282	0.081	0.382	2.575	1.179	0.765
	*Tachyglossus aculeatus*	85	260	0.073	0.389	2.709	1.183	0.791
Notoryctemorphia	*Notoryctes typhlops*	112	363	0.058	0.455	2.736	1.485	0.806
Paucituberculata	*Caenolestes fuliginosus*	122	383	0.052	0.435	2.767	1.544	0.770
Peramelemorphia	*Macrotis lagotis*	120	396	0.055	0.434	2.72	1.541	0.790
Perissodactyla	*Equus caballus*	124	327	0.043	0.454	2.874	1.555	0.774
	*Tapirus indicus*	114	321	0.05	0.463	2.862	1.463	0.815
Pholidota	*Manis pentadactyla*	101	292	0.058	0.456	2.873	1.375	0.791
Pilosa	*Bradypus tridactylus*	110	318	0.053	0.548	2.909	1.293	0.782
	*Choloepus didactylus*	90	255	0.064	0.489	2.708	1.339	0.762
	*Cyclopes didactylus*	96	260	0.057	0.456	2.917	1.319	0.820
Primates	*Homo sapiens*	113	334	0.053	0.512	2.712	1.442	0.793
	*Loris tardigradus*	114	344	0.053	0.352	2.731	1.47	0.808
	*Macaca mulatta*	122	369	0.05	0.344	2.719	1.504	0.812
Proboscidea	*Elephas maximus*	110	273	0.046	0.439	2.829	1.424	0.797
Rodentia	*Chinchilla lanigera*	108	320	0.055	0.404	2.761	1.423	0.831
	*Heteromys desmarestianus*	96	294	0.064	0.356	2.719	1.256	0.803
	*Neotoma fuscipes*	120	333	0.047	0.409	2.825	1.521	0.814
	*Pedetes capensis*	112	323	0.052	0.434	2.775	1.474	0.802
	*Sciurus vulgaris*	130	364	0.043	0.421	2.807	1.575	0.780
Scandentia	*Ptilocercus lowii*	114	336	0.052	0.483	2.785	1.49	0.719
Sirenia	*Dugong dugon*	106	310	0.056	0.505	2.782	1.413	0.795
Tubulidentata	*Orycteropus afer*	101	272	0.054	0.504	2.86	1.308	0.783

*N* Number of elements, *K* Number of connections, *D* Density of connections, *C* Mean clustering coefficient, *L* Mean shortest path length, *H* Heterogeneity of connections, *P* Parcellation index

**Table 3:** Variability and phylogenetic signal in neck network parameters

	N	K	D	C	L	H	P
Minimum	84	219	0.043	0.295	2.575	1.179	0.71
1st Quartil	102.5	293.5	0.052	0.41	2.72	1.371	0.769
Mean	108.6	321.5	0.056	0.437	2.782	1.421	0.782
3rd Quartil	115.8	345	0.058	0.464	2.83	1.493	0.802
Maximum	130	397	0.081	0.548	3.099	1.575	0.844
Coefficient of Variation	0.097	0.132	0.128	0.114	0.031	0.066	0.04
95% Confidence Intervalls	0.077/0.115	0.104/0.155	0.092/0.16	0.085/0.138	0.022/0.04	0.051/0.079	0.032/0.047
Abouheif’s Cmean	0.372***	0.421***	0.366***	0.133	0.172	0.359***	0.354***
Blomberg’s K	0.995***	0.879***	1.479***	0.559	0.762	1.108***	0.719**

*N* Number of elements, *K* Number of connections, *D* Density of connections, *C* Mean clustering coefficient, *L* Mean shortest path length, *H* Heterogeneity of connections, *P* Parcellation index. Significance levels of the tests for phylogenetic signal: *p* < 0.01**; p < 0.001***

**Fig. 3:**
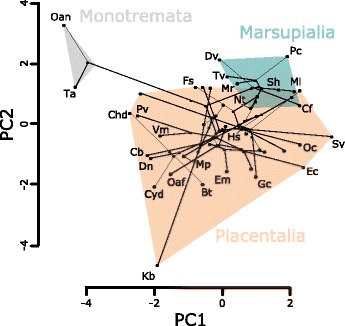
Phylomorphospace of the network parameters. Monotreme, marsupial and selected placental species are labeled. PC1 and PC2 represent 44% and 27% of the total variation, respectively. Species abbreviations: Bt *Bradypus tridactylus*; Cb *Camelus bactrianus;* cf *caenolestes fuliginosus*; Chd *Choloepus didactylus*; Cyd *Cyclopes didactylus*; Dn *Dasypus novemcinctus*; Dv *Didelphis virginiana*; Ec *Equus caballus*; Em *Elephas maximus*; Fs *Felis silvestris*; Gc *Galictis cuja*; Hs *Homo sapiens*; Kb *Kogia breviceps*; Ml *Macrotis lagotis*; Mp *Manis pentadactyla*; Mr. *Macropus rufus*; Nt *Notoryctes typhlops*; Oaf *Orycteropus afer*; Oan *Ornithorhynchus anatinus*; Oc *Oryctolagus cuniculus*; PC *Phascolarctos cinereus*; Pv *Pteropus vampyrus*; Sh *Sarcophilus harrisii*; Sv *Sciurus vulgaris*; Ta *Tachyglossus aculeatus*; Tv *Trichosurus vulpecula*; Vm *Vespertilio murinus*

The number of anatomical elements *N* is low in monotremes compared to the general pattern of therians (Fig. 2a). However, most xenarthrans, the chiropterans, and the Pygmy sperm whale (*Kogia breviceps*) also have a decreased number of elements in their neck network. The number of anatomical connections K is uniformly high in marsupials in contrast to most other mammals (Fig. 2b). *K* is decreased in monotremes, xenarthrans, chiropterans, the Pygmy sperm whale and the Bactrian camel (*Camelus bactrianus*). Morphological complexity *D* is high in monotremes, intermediate in marsupials and xenarthrans and tends to decrease in most of the other placental lineages (Fig. 2c). For H, the largest contrast can be found between monotremes (very low H) and therians in general, whereas the pattern within therians is not uniform (Fig. 2d). The degree of modularity *P* is relatively invariable and only slight decreases can be found in diprotodonts, Pen-tailed treeshrew (*Ptilocercus lowii*), chiropterans and eulipotyphlans (Fig. 2e). The phylomorphospace (Fig. 3; Additional file 1: AF1) highlights the differences in neck musculoskeletal organization between monotreme and therian mammals (F = 1.695, *p* < 0.001). Marsupials cluster closely together. They slightly overlap with the distribution of placental mammal, which occupy a huge part of the morphospace. The Pygmy sperm whale is far from the other placental mammals.

### Community structure and phenotypic modules in neck networks

**Fig. 4:**
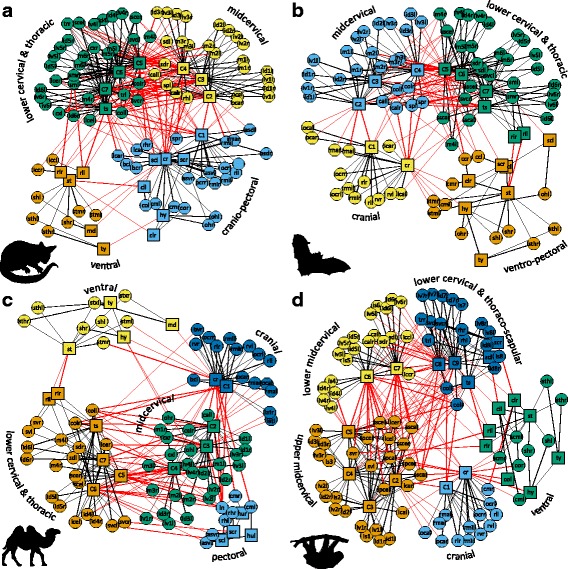
Network representations and connectivity modules of the neck of different mammals. **a** Common brushtail possum (*Trichosurus vulpecula*); (**b**) Parti-coloured bat (*Vespertilio murinus*); (**c**) Bactrian camel (*Camelus bactrianus*); (**d**) Three-toed sloth (*Bradypus tridactylus*). Colors code for the identified connectivity modules. Red links between-modules. Black links within modules

The number and constitution of the connectivity modules in the neck varies among the mammalian species examined (a summary is given in Table 4; for detailed information see Additional file 1: AF1). In many cases however, module number varies based on a left-right split of modules that are united in other species (e.g., the pectoral elements are separated in left and right modules). Overall, five principal connectivity modules were detected: 1) cranio-pectoral, 2) ventral, 3) mid-cervical, 4) lower cervical, and 5) thoracic. This pattern is exemplified here for the lesser grison (*Galictis cuja*) (Fig. 1g). The cranio-pectoral module (present in 32 out of 48 taxa) groups the cranium, the C1, and the bones of the pectoral girdle (scapulae, claviculae, and, if included, humeri), as well as the suboccipital, cleidocephalic (or cephalohumeralis), atlantoscapularis, capital longus, and capital rhomboid muscles. The ventral module groups the sternum, hyoid, thyroid, mandible (when included), and the sternocephalic and infrahyoid muscles. In 32 out of 48 cases, ribs and the related scalenii muscles are also included in this module. The ventral module is combined with (parts of) the pectoral bones and muscles in 15 species, none of which are aclaviculate (Fig. 4b). The mid-cervical module groups C2 to C4 as well as the longus cervicis, spinalis, and their related interspinal, intertransversarii, and multifidii muscles. The lower cervical module groups C5 to C7 with the cervical longissimus, spinalis, and their related interspinal, intertransversarii, and multifidi muscles. These mid-cervical and lower cervical modules are present in all networks (Fig. 4a-d). The border between them is, however, shifted in some species (e.g., C4/C5 to C3/C4). If the attachments of scalenii muscles are limited to few specific cervical vertebrae, these muscle and the ribs are also included in the mid or lower cervical module. The thoracic module groups the thoracic spine, nuchal ligament, semispinalis (complexus + biventer cervicis), capital longissimus, cervical rhomboid and trapezius muscle.

**Table 4:** Summary of network modules

Order	Species	M1	M2	M3	M4	M5
Afrosoricida	*Chrysospalax trevelyani*	**cranio-atlantal pectoral**	midcervcial	lower cervical - thoracic	ventral	
	*Micropotamogale ruwenzorii*	cranio-atlantal	midcervcial^a^	lower cervical - thoracic	ventral	**pectoral**
Carnivora	*Canis lupus*	**cranio-atlantal pectoral**	midcervical	lower cervical	ventral	thoracic
	*Civettictis civetta*	**cranio-atlantal pectoral**	midcervcial	lower cervical - thoracic	ventral	
	*Felis silvestris*	**cranio-atlantal pectoral**	midcervcial	lower cervical	ventral	thoracic
	*Galictis cuja*	**cranio-atlantal pectoral**	midcervcial	lower cervical	ventral	thoracic
	*Zalophus californianus*	cranio-atlantal	midcervcial	lower cervical - thoracic	ventral	**pectoral**
Cetartiodactyla	*Babyrousa babyrussa*	**cranio-atlantal humeral**	midcervical	lower cervical - thoracic	ventral	**scapular**
	*Bos taurus*	**cranio-atlantal humeral**	midcervcial	lower cervical - thoracic	ventral	**scapular**
	*Camelus bactrianus*	cranio-atlantal	midcervical	lower cervical - thoracic	ventral	**pectoral**
	*Giraffa camelopardalis*	cranio-atlantal	**midcervcial-pectoral**	lower cervical	ventral	thoracic
	*Kogia breviceps*	cranio-atlantal costal	midcervcial	lower cervical - thoracic	ventral	**pectoral**
Chiroptera	*Pteropus vampyrus*	cranio-atlantal	midcervcial	lower cervical - thoracic	**ventro-pectoral**	
	*Vespertilio murinus*	cranio-atlantal	midcervcial	lower cervical - thoracic	**ventro-pectoral**	
Cingulata	*Dasypus novemcinctus*	cranio-atlantal axial	midcervcial	lower cervical	ventral	thoracic
Dasyuromorpha	*Sarcophilus harrisii*	cranio-atlantal	midcervcial	lower cervical - thoracic	ventral	pectoral
Didelphimorphia	*Didelphis virginiana*	**cranio-atlantal pectoral**	midcervcial	lower cervical - thoracic	ventral	
Diprotodontia	*Macropus rufus*	**cranio-atlantal pectoral**	midcervcial	lower cervical - thoracic	ventral^d^	
	*Phascolarctos cinereus*	**cranio-atlantal pectoral**	midcervcial	lower cervical - thoracic	ventral	
	*Trichosurus vulpecula*	**cranio-atlantal pectoral**	midcervcial	lower cervical - thoracic	ventral	
Eulipotyphla	*Erinaceus europaeus*	**cranio-atlantal pectoral**	midcervcial	lower cervical - thoracic	ventral	
	*Scalopus aquaticus*	**cranio-atlantal pectoral**	midcervcial	lower cervical - thoracic	ventral	
	*Suncus murinus*	atlantal	midcervcial	lower cervical - thoracic	**cranio-ventro-pectoral**	costal
Hyracoidea	*Procavia capensis*	**cranio-atlantal pectoral**	midcervcial	lower cervical	ventral	thoracic
Lagomorpha	*Oryctolagus cuniculus*	**cranio-atlantal pectoral**	midcervical^b^	lower cervical - thoracic	ventral	
Monotremata	*Ornithorhynchus anatinus*	cranio-atlantal	midcervcial	lower cervical - thoracic	**ventro-pectoral**	
	*Tachyglossus aculeatus*	cranio-atlantal axial	midcervcial	lower cervical - thoracic	ventral	**pectoral**
Notoryctemorphia	*Notoryctes typhlops*	cranio-atlantal	midcervcial	lower cervical	**ventro-pectoral**	thoracic
Paucituberculata	*Caenolestes fuliginosus*	**cranio-atlantal pectoral**	midcervical^c^	lower cervical - thoracic	ventral	
Peramelemorphia	*Macrotis lagotis*	**cranio-atlantal pectoral**	midcervcial^a^	lower cervical - thoracic	ventral	
Perissodactyla	*Equus caballus*	**cranio-atlantal pectoral**	midcervcial	lower cervical	ventral	thoracic
	*Tapirus indicus*	**cranio-atlantal pectoral**	midcervcial	lower cervical	ventral	thoracic
Pholidota	*Manis pentadactyla*	**cranio-atlantal pectoral**	midcervical^b^	lower cervical - thoracic	ventral	
Pilosa	*Bradypus tridactylus*	cranio-atlantal	upper midcervical	lower midcervical	**ventro-clavicular**	**lower cervical - thoracic scapular**
	*Choloepus didactylus*	cranio-atlantal	midcervcial	lower cervical - thoracic	ventral	**pectoral**
	*Cyclopes didactylus*	cranio-atlantal	midcervcial - thoracic	lower cervical C5&rest	ventral	**pectoral**
Primates	*Homo sapiens*	cranio-atlantal	midcervical^b^	lower cervical - thoracic	ventral	**pectoral**
	*Loris tardigradus*	**cranio-atlantal pectoral**	midcervcial^a^	lower cervical - thoracic	ventral	thoracic
	*Macaca mulatta*	**cranio-atlantal pectoral**	midcervical^c^	lower cervical - thoracic	ventral	costal
Proboscidea	*Elephas maximus*	**cranio-atlantal pectoral**	midcervical^c^	lower cervical - thoracic	ventral	
Rodentia	*Chinchilla lanigera*	**cranio-atlantal scapular**	midcervical	lower cervical	**ventro-clavicular**	thoracic
	*Heteromys desmarestianus*	cranio-atlantal	midcervcial	lower cervical - thoracic	ventral	**pectoral**
	*Neotoma fuscipes*	**cranio-atlantal pectoral**	midcervical	lower cervical - thoracic	ventral	
	*Pedetes capensis*	**cranio-atlantal pectoral**	midcervcial^a^	lower cervical - thoracic	ventral	
	*Sciurus vulgaris*	**cranio-atlantal scapular**	midcervcial	lower cervical - thoracic	**ventro-clavicular**	
Scandentia	*Ptilocercus lowii*	**cranio-atlantal pectoral**	midcervcial	lower cervical - thoracic	ventral	
Sirenia	*Dugong dugon*	**cranio-atlantal pectoral**	midcervcial	lower cervical	ventral	thoracic
Tubulidentata	*Orycteropus afer*	**cranio-atlantal pectoral**	midcervical^b^	lower cervical - thoracic	ventral	

Contribution of the pectoral elements to different modules marked in bold. Left-right division of pectoral and costal elements is not considered in this summary table. Scapular, humeral, and clavicular elements are separately indicated when the pectoral bones and associated muscles are not group within the same module

^a^ no clear assignment of C5 to the midcervical or lower cervical module

^b^ potential subdivision of the midcervical module into C2/C3 and C4/C5

^c^ no clear assignment of C2 to the cranio-atlantal or midcervical module

^d^ no clear division between the cranio-atlantal and ventral module

In several species, the pectoral bones (plus the related muscles) are not grouped together with the cranium and C1 but separated; otherwise they are included in the ventral or thoracic module, respectively. For instance, in the long-necked camel the pectoral bones and muscles constitute a distinct module together with the nuchal ligament (Fig. 4c). In the giraffe (*Giraffa camelopardalis*), attachment sites of the pectoral muscles are shifted proximally to the midcervical module. In contrast, the pectoral bones and muscles are combined with the ventral elements into a ventro-pectoral unit in the parti-colored bat (*Vespertilio murinus*) (Fig. 4b). The sloths differ in the organization of their neck due to their aberrant number of cervical vertebrae. In the two-toed sloth (*Choloepus didactylus*; six cervical vertebrae), the pectoral elements form a separate module. The C5, C6, thoracic spine, and related muscles are grouped within one module. In the three-toed sloth (*Bradypus tridactylus*; nine cervical vertebrae; Fig. 4d), there is an upper (C2-C5) and lower (C6-C7) midcervical module. The evolutionary ‘new’ vertebrae C8 and C9, however, are grouped with the thoracic spine, scapulae, and related muscles. The claviculae are included in the ventral module. The ribs are grouped with the cranium and atlas in the Pygmy sperm whale.

### Disparity in neck organization through time

The mean subclade disparity values for the observed and simulated data were plotted against node age (Fig. 2f). Subclade disparity through time is low, which is particularly obvious in the first two-thirds of mammalian evolution (i.e., during the Mesozoic). However, it is not significantly different from the expectation under a Brownian motion model of neck organizational evolution (morphological disparity index = 0.016, *p* = 0.65). Nevertheless, major shifts in disparity rate occurred in the middle to late Paleocene and in the middle to late Eocene. These shifts resulted in disparity peaks exceeding the 95% confidence interval of the simulated data. The disparity in neck musculoskeletal organization decreased after the Eocene-Oligocene border. A larger sample size would be required to infer significant results for the post-Eocene ages (i.e., more divergence events are needed).

## Discussion

### Variation in neck organization across phylogeny

The more conserved network parameters (*L*, *H*, *P*) represent measurements of the neck’s integration by effective proximity, anisomerism, and degree of modularity [41, 43]. These measurements capture how distant parts of the neck (e.g., head and trunk, lower and upper cervical column) are integrated, that anatomical connections are not evenly distributed across bone (e.g. vertebrae) and the way the neck is modularized. Thus, our results indicate a basic constructional set-up of the neck across mammals determined by morphological regionalization (see further below). These conserved features likely arise due to shared developmental [8, 9, 17, 19, 59–61] and/or biomechanical/constructional constraints [31, 62, 63]. However, the musculoskeletal organization of the neck is not uniform for other morphological features captured by network proxies. Specifically, mammalian necks considerably vary in terms of their morphological burden, complexity, and integration by co-dependency (quantified by *K*, *D*, and *C*, respectively). These features describe the grade of specialization in the neck due to reduction of elements or enhancement of passive structures (e.g., the nuchal ligament) and the way the neck is structurally constrained by the setup of its muscular connections (i.e., its evolvability) [43, 49]. The variation arises from the major trends of epaxonic muscle modification during mammalian evolution, leading to differences in nuchal muscle organization among monotremes, marsupials and therians. Our findings confirm the plesiomorphic pattern of epaxonic neck muscle arrangement in monotremes [64–67]. It results in a musculoskeletal organization that is distinctively different from that of therian mammals (Fig. 3). Their low differentiation within the three longitudinal systems (longissimus, iliocostalis, transversospinalis; in particular the deep intervertebral muscles; low number of muscles) and muscle attachments that are evenly distributed among the vertebrae (high complexity but low irregularity) suggest low specialization to specific neck motion patterns. In marsupials, epaxonic muscles are more differentiated in deep and superficial layers. Moreover, most of the superficial muscles are attached to every cervical vertebra [66, 68–72]. This high connectivity results in structural constraints in the neck (i.e., morphological burdens) and low musculoskeletal disparity among marsupials in comparison to placentals (Fig. 3). Attachments of epaxonic neck muscles are very variable among placental mammals [70] and thus network parameters are as well. However, two major trends in neck evolution have been shown: First, there is a reduction of attachment sites of the neck muscles to only a few vertebrae/the skull; and second, there is an increased bracing of the head-trunk distance by ligamentous structures to accommodate for increasing head weight and neck length [15, 73–78]. This results in placental mammals generally having necks that are less complex compared to monotreme and marsupial mammals. For instance, few but specialized muscles have the small attachment sites and are able to induce a similar motion or the superficial epaxonic muscles attach only secondarily to the head via the nuchal ligament. In addition, neck variation in placental mammals is also highly influenced by variation in the organization of pectoral bones and muscles (see below).

The phylogenetic signal of most of the network parameters reveals that phylogenetic relationship accounts for much of the variation in neck organization. At the same time, network parameters also discriminate between monotreme, marsupial, and placental mammals. Within placental mammals, however, variation of network parameters is mostly limited to xenarthrans, chiropterans, and some cetartiodactyls. Although relatively species-poor, xenarthrans, show highly specialized neck morphologies related to their diverse fossorial or arboreal ecologies [79–81] and unique development [33, 82, 83]. In chiropterans, back muscles contribute only marginally to the stabilization of the head because of the lack of most cranial and cervical attachments [84–86]. Sagittal stability is instead achieved by the modified morphology of the cervical vertebrae in accordance with roosting behaviors [87]. Cetartiodactyls have recently been show to exhibit the highest disparity in neck morphology across mammals [31]. It ranges from the very short necks of cetaceans up to the extreme long ones of camelids and giraffids. As a consequence, neck musculoskeletal organization is similarly diverse. Several muscles with cervical attachment are reduced in the Pygmy sperm whale (and other cetaceans) or their attachment is shifted to the skull (e.g. scaleni muscles) and thus head stabilization is increased [88, 89]. On the other hand, cranial attachments of the dorsal neck muscle are mainly reduced in long necked species, such as the camel and giraffe. Muscle force is instead transferred by the modified nuchal ligament [74, 75, 77]. Surprisingly, neck network parameters in the dugong (*Dugong dugon*), although also being fully aquatic, do not show a similar alteration as in the Pygmy sperm whale. Instead, it closely resembles the Asian elephant (*Elephas maximus*) and other afrotherians (Table 1, Fig. 2).

In accordance with our findings, [90, 91] also showed that the effect of size and prey capture behavior is low in the neck compared to the thoracolumbar region. However, functional interpretations of the results of the analysis of the topological arrangement of parts needs to be inferred on a one to one basis and taking into account the specific ecological context of each taxa.

### Regionalization and modularity in the mammalian neck

Despite the relative low and invariant number of neck vertebrae in mammals, several studies have uncovered a tripartite regionalization of the cervical spine based on developmental, morphological, allometric, and functional evidence [17, 25, 31, 32, 91]. Our results have now uncovered a corresponding regionalization of the musculoskeletal organization of the mammalian neck into an upper (cranium, C1), mid (C2-C4), and lower cervical module (C5-C7, in some species also the thoracic spine). This modularity pattern is conserved across mammals despite variations in size, feeding mechanisms, and locomotor modes (indicated by a uniform grade of modularity). This conserved pattern probably arose from the high number of connections between the vertebrae of the same module (or the cranium and C1) resulting in an increase of structural constraints and integration of these elements [40, 49, 52]. However, the boundaries between adjacent modules/regions are not consistent across different studies analyzing the morphology of the neck using different criteria. For example, vertebrae C1 and C2 are not part of the same connectivity module despite their close developmental, functional, and evolutionary relationship [25, 92, 93]. A similar dissociation of C1 and C2 into different regions has been shown for their scaling properties [31] and highlights the role of C2 as a functional mediator between the head joint and the postaxial column (see also [94]). In addition to the three ‘inner’ axial modules, there are two additional ‘outer’ modules bridging the distance between the trunk and the head (or the hyoid or upper vertebrae), with a muscular cuff on the dorso-lateral (pectoral) and ventro-lateral side. Many of these muscles were crucial for the evolutionary origin of the vertebrate neck [95, 96].

### Neck organization in sloths

In general, a similar regionalization of the neck is observed in both genera of sloths, despite their variation in the number of cervical vertebrae. The evolutionary new C8 and C9 in *Bradypus* and their associated muscles are grouped together with the thoracic spine. This agrees with their thoracic origin and ossification sequence [82]. Conversely, the evolutionary new Th1 provides the basis for the close association of the thoracic vertebral region to C5 and C6 in *Choloepus* neck organization. Divergence of the sloths’ necks becomes obvious when including their pectoral bones and muscles in the comparison. Their neck-shoulder arrangement represents two different solutions of locomotor possibilities under common functional constraints [80, 97]. Neck organization and modularity of *Bradypus* resembles those of other long necked species. The resemblance stems from its nearly complete lack of cervical and cranial attachments of the neck/shoulder muscles and its unusual clavicular and shoulder morphology [80, 98–100] (see the unusual lower cervical-thoracic-scapular module in Table 4). The muscles of *Choloepus*, in contrast, are so placed as to offer the greatest possible support dorsally and ventrally to the head as well as to the scapula [80, 98, 101, 102]. Thus, neck organization and modularity is closer to the general pattern as seen in the lesser grison (i.e., pronounced head support, functional connection of head and forelimb) [15].

### Evolutionary integration of the neck and the forelimb

The enduring evolutionary and developmental relationship between the neck and the forelimb in mammals (and other amniotes) is well documented (e.g., [17, 19, 20, 95, 96, 103]). This relationship is most obvious in the brachial plexus innervating shoulder and forelimb muscles [104, 105]. However, there is also a strong functional integration between the neck and the forelimb, with several muscles connecting the pectoral girdle and the head/neck (often with repeated slips). Based on this functional connection, the posture and movements of the neck have a crucial influence on the mechanics of the forelimb in terms of gait efficiency, balance, stabilization, ground reaction forces, and kinematics [30, 106–112]. Our findings now highlight the consequences of this integration on the musculoskeletal organization of the neck. Although there is a conserved tripartition of the cervical spine and its associated muscles, the varying contribution of pectoral bones and muscles to different connectivity modules accounts for much of the observable neck disparity across mammals (e.g., see results on sloths). Major shifts in forelimb morphology and function (e.g., mobilization of the pectoral girdle, reduction of the claviculae) [113–115] are associated with increasing decoupling of the pectoral elements from the ventral module and their connection to the cranium and upper cervical region. This coincides with the increased role of head/neck movements on forelimb mechanics during fast and endurance running (i.e., cursoriality) [30, 108]. In mammals with extreme long necks (camel, giraffe), although also being capable of enduring walking, the pectoral bones and muscles are separated from the cranial module. However, it has recently been shown that neck pendulum mechanics and function in long-necked mammals is different compared to actual cursors [108].

### Implications for the evolution and disparity of the mammalian neck

**Fig. 5:**
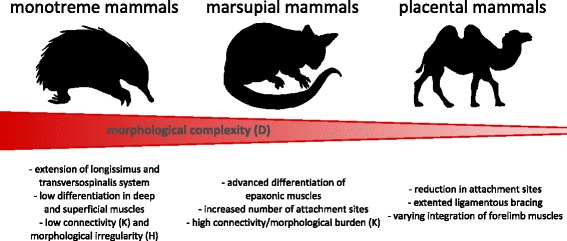
Differences in the musculoskeletal organization of the neck between monotreme, marsupial, and placental mammals. In general. The morphological complexity of the neck (estimated by the density of connections D) decreases from monotreme to placental mammals (red bar) despite the disparity in neck organization and neck length among the latter

The differences in neck organization between monotremes and therian mammals is one of the striking findings of this study. They result in high disparity between them whereas their within-subclade disparity is low during the first two-thirds of mammalian evolution. Accordingly, the disparity of the neck of mammals was low during the Mesozoic (Fig. 2f) [116]. Figure 5 illustrates the major grades of musculoskeletal organization during mammalian neck evolution. Monotreme, marsupial, and placental mammals differ in their degree of epaxonic muscle differentiation and the varying integration of the forelimb muscles. In general, the morphological complexity of the neck decreases from monotremes to placentals (Fig. 5) but disparity increases (Fig. 3).

The therian radiation that followed the K/T mass extinction and the appearance of most of the therian and marsupial (supra)orders during the Paleocene was accompanied by an abrupt increase in neck musculoskeletal disparity after a long period of low neck disparity. This was even associated with the appearance of locomotor and foraging specializations [117] and the diversification in body size (e.g., [118]). The second disparity peak during the Eocene coincides with the radiation and increased diversity of modern placental orders, like cetartiodactyls, perissodactyls, carnivorans, and xenarthrans (see [117] and references therein). Thus, disparity in neck organization emerged relatively late in the long mammalian history and is associated with the origin and radiation of specific lineages.

## Conclusion

One of our crucial findings is that the musculoskeletal organization of the neck differs between monotreme, marsupial, and placental mammals (Fig. 5). Moreover, particularly the necks of placental mammals are characterized by a reduced complexity despite their increased disparity in musculoskeletal organization and length. Our network analyses revealed a mosaic complexity and disparity in the musculoskeletal organization of the mammalian neck despite the more obvious meristic (and other) constraints on the cervical spine. Musculoskeletal irregularity, effective proximity, degree of modularity, and the occurrence of three inner/axial regions are conserved features among mammalian necks. Thus, a shared biomechanical construction and common developmental interrelationships not only constrain variation in the cervical spine, but are similarly likely to limit musculoskeletal variability in the neck. The conservation of these traits contrasts, however, with the high variability in morphological burden, integration by co-relation, morphological complexity, and the configuration of the ventral and (cranio-)pectoral module in the neck. The expansion of limb muscles in the cervical region not only facilitated enhanced forelimb mechanics but also increased structural disparity (and thus derived motor patterns and mechanics) in the neck. Thus, we highlight the close integration of the neck and the forelimb during mammalian evolution. The disparity in neck musculoskeletal organization evolved late in mammalian history and in parallel with the radiation of some lineages (e.g., cetartiodactyls, xenarthrans). Finally, our findings show that the limited number of vertebrae in the cervical spine does not necessarily result in low musculoskeletal disparity during mammalian evolutionary diversification.

## Methods

### Data collection and anatomical network modeling

We collected the topographic data of the musculoskeletal system of the neck in 48 mammalian species through an extensive literature review (Table 2, Additional file 2: AF2). The sample represents all major monotreme, marsupial, and placental clades, as well a diversity of locomotor and feeding strategies. Rodent diversity is represented by members of all suborders (sciuromorphs, myomorphs, hystricomorphs, castorimorphs, and anumaluromorphs, respectively). Representatives of both genera of extant sloth (*Bradypus* and *Choloepus*) were included to examine the influence of their deviating number of cervical vertebrae on neck organization. We documented the number and specific connections/attachments of all skeletal structures and muscles constituting the neck motion system in these taxa.

In contrast to the domestic mammals, for which veterinary textbooks as well as anatomical publications are available, information for exotic species is scarce and literature descriptions are in many cases old. To overcome this problem, in all but two cases we always consulted two or more references to compare the data. We made sure that at least one detailed anatomical monograph was included. As the network models used here focus on the presence/absence of attachments among bones and muscles rather than more specific information on the nature and area of such attachments, the literature reviewed was of enough quality for our modeling approach.

We included all muscles originating from the cervical vertebrae, skull (cranium or mandible), nuchal ligament, or hyoid/thyroid, and inserting on the (cervical or thoracic) vertebrae, sternum, pectoral girdle (scapulae, claviculae, humeri), or ribs (see details in Additional file 3: AF3). Accordingly, we excluded the masticatory, facial, laryngeal, pharyngeal, and suprahyoid muscles from the analysis. A calibrated phylogenetic tree was constructed using the data from the Timetree of Life database [119, 120] (Fig. 2).

We built anatomical network models of the necks’ musculoskeletal systems, which comprised all anatomical units as well as the different types of physical interaction among them. Network nodes represented all bones (cranium, cervical vertebrae, thoracic spine, left and right ribs, hyoid, left and right claviculae, left and right scapulae, sternum, and, if involved, left and right humeri, and mandible), other passive elements (nuchal ligament, if present, thyroid), and all cervical muscles, as described above. Network connections represented all physical articulations between bones and other passive elements described, as well as the fleshy and tendinous attachments of the muscles onto the bones (see adjacency matrices in Additional file 4: AF4). Network models were analyzed using the *igraph* package [121] in R [122].

### Network parameter analyses

The mathematical definitions and calculations of the network parameters examined here (*N*, *K*, *D*, *C*, *L*, *H*, *P*) are provided in Table 1. The degree of modularity (parcellation *P*) was measured from the connectivity modules identified using a spin-glass model and simulated annealing algorithm implemented in the R package *netcarto* [123, 124]. A connectivity module is defined as a group of nodes highly connected among them and poorly connected to nodes outside the group. *P* is 0 when all nodes are in a same module, and tends to 1 when nodes are evenly distributed within many modules. We tested the phylogenetic signal in the multivariate dataset of all network parameters by calculating K_mult_ [125] using R package *phylocurve* [126]. We additionally tested the phylogenetic signal of the individual parameters using the Abouheif’s test with 1000 permutations [127] and Blomberg’s test [128] in the R package *phytools* [129]. Mode of trait evolution was explored by comparing multivariate fits of Brownian motion, Ornstein-Uhlenbeck (single adaptive optimum), and Early burst models using Akaike information criterion (AIC) weights in R package *mvMORPH* [130]. Distribution of the network parameters was visualized with a phylomorphospace of the first two principal components using R package *phytools* [129].

Relative variability of the network parameters was analyzed by statistical comparison of their coefficients of variation (CVs). 95% confidence intervals of the CVs were calculated by 10,000 bootstrap resampling. Significant differences among the parameters’ CVs were tested using the modified signed-likelihood ratio test (MSLRT) for equality of CVs (all parameters) and the asymptotic test for the equality of CVs (pair-wise comparisons, Bonferroni corrected) in the R package *cvequality* [131]. In order to test for allometric effects on network parameters they were regressed against logtransformed body mass, absolute neck length, and relative neck length. Body mass and neck length data were taken from [31]. Relative neck lengths were calculated by dividing absolute neck length by body mass^1/3^. Allometric analyses were done using phylogenetic generalized least square regressions in the R package *caper* [132]. The effect of predatory behavior on logtransformed network parameters was examined by testing for significant differences between predatory and non-predatory mammals using a multivariate distance-based phylogenetic generalized least square regression (D-PGLS) with 1000 permutations in the R package *geomorph* [133]. Species were classified as predatory when food intake involves head-neck movements to hold the food counteracting its resisting movements (carnivorous, insectivorous, piscivorous species). In contrast, species were classified as non-predatory when food intake does not involve such head-neck movements (food is just picked or harvested, e.g., browsers, grazers, but also myrmecophagous species). Differences in network parameters between monotreme, marsupial, and placental mammals were tested using a multivariate D-PGLS regression with 1000 permutations.

### Modularity analysis

We calculated the quality of the partitions identified by the community detection algorithm using the optimization function Q [134]. According to Newman and Girvan [134], if the number of connections within modules is not different from that expected at random, then Q will be close to 0. The higher the Q the stronger the modular pattern of the network (Q_max_ = 1). In practice, strongly modular networks show Q values ranging from 0.3 to 0.7 [134]. Thus, we considered that an anatomical network has a strongly modular structure if Q − Q_error_ > 0.3. The expected error of Q was calculated using a jackknife procedure, where every link was taken as an independent observation [134] (more details are provided in Additional file 3: AF3). Finally, we estimated the statistical significance of each module using a two-sample Wilcoxon rank-sum test on the internal vs. external links of the module’s nodes. The null hypothesis was that the number of connections is the same inside as outside the module (i.e., as expected if the module were created at random); the alternative hypothesis was that the number of connections is higher inside than outside the module (i.e., the definition of connectivity module). An extensive account of these methods has been given elsewhere [41, 48, 135].

### Disparity through time analysis

We carried out a disparity through time (DTT) analysis using the R package *geiger* [136] to trace the variation in neck organization through the evolution of mammals. First, we performed a principal component analysis (PCA) of the network parameters used as proxies of the morphological organization of the neck (i.e., *N*, *K*, *C*, *D*, *L*, *H*, and *P*) to account for their co-variation structure. Mean subclade disparity through time for the PC scores were calculated [116, 137]. Observed disparity in neck organization across our phylogeny was compared with that expected under a Brownian motion process performing 10,000 iterations. High disparity values indicate high variance within subclades; low disparity values indicate conservation within subclades and high variance among subclades. Finally, we calculated the morphological disparity index to quantify the overall difference in relative disparity of a clade compared to that expected under the null Brownian motion model [116, 137].

### Abbreviations of network elements

Left and right side are indicated by *l* and *r*, respectively, added to the abbreviations.

*asd* atlantoscapularis dorsalis; *asv* atlantoscapularis ventralis; *bc* biventer cervicis; *C1-C9* cervical vertebrae; *cal* longus capitis; *cc* cleidocervicalis; *cl* clavicle; *cm* cleidomastoideus; *co* cleidooccipitalis; *col* longus colli; *cx* complexus; *cr* cranium; *hu* humerus; *hy* hyoid; *icc* iliocostalis cervicis; *id1-id8* intertransversarii cervicis dorsales; *im1-im4* intertransversarii cervicis mediales; *is1-is8* interspinalis; *iv/iv1-iv8* intertransversarii cervicis ventrales (fused/separate); *lat* longus atlantis; *lca* longissimus capitis; *lce* longissimus cervicis; *ln* nuchal ligament; *m1-m6* multifidi; *md* mandible; *oca* obliquus capitis caudalis; *ocr* obliquus capitis cranialis; *oh* omohyoideus; *rca* rhomboideus capitis; *rce* rhomboideus cervicis; *rci* rectus capitis dorsalis intermedius; *rh* rhomboideus (undifferentiated); *ri* ribs; *rl* rectus capitis lateralis; *rma* rectus capitis dorsalis major; *rmi* rectus capitis dorsalis minor; *rv* rectus capitis ventralis; *sce* spinalis cervicis; *sc* scapula; *scm* sternocleidomastoideus (sternal and clavicle part not separate); *sd* scalenus dorsalis; *sh* sternohyoideus; *sm* scalenus medius; *so* sternooccipitalis; *spca* splenius capitis; *spce* splenius cervicis; *sp* splenius (undifferentiated); *ssca* semispinalis capitis; *ssce* semispinalis cervicis; *st* sternum; *sth* sternothyroideus; *stm* sternomastoideus; *stx* sternomaxillaris; *svc* serratus ventralis cervicis; *sv* scalenus ventralis; *tr* trapezius; *ts* thoracic spine; *ty* thyroid

## Additional files

Additional file 1: AF1 Additional information on the results of phylogenetic, network, and modularity analyses. (PDF 1108 kb)

Additional file 2: AF2 Systematics and references of investigated species. (PDF 414 kb)

Additional file 3: AF3 Additional information on methods. (PDF 210 kb)

Additional file 4: AF4 Adjacency matrices coding for the topological information of neck’s musculoskeletal organization of investigated species. (XLS 677 kb)
