# Supplementary material for: Musculoskeletal networks reveal topological disparity in mammalian neck evolution
Source: BMC Evol Biol. 2017 Dec 13;17:251. doi: 10.1186/s12862-017-1101-1 (PMC5729486; doi:10.1186/s12862-017-1101-1)
Supplement: Supplementary file 1 — AF1 Additional information on the results of phylogenetic, network, and modularity analyses. (PDF 1108 kb) [file 12862_2017_1101_MOESM1_ESM.pdf]

Musculoskeletal networks reveal topological disparity in mammalian neck evolution

Patrick Arnold*, Borja Esteve-Altava, Martin S. Fischer

Additional file AF1_results

*author of correspondence: patrick_arnold@eva.mpg.de

Phylogenetic Analysis

Table A1 Abouheif's test results

	Observed	Standard deviation	p-value
N	0.3716330	3.6217111	0.0009990
K	0.4205506	4.0252417	0.0009990
D	0.3664639	3.9855066	0.0009990
C	0.1329669	1.3724624	0.1878122
L	0.1715729	1.7239277	0.0899101
H	0.3588361	3.5817741	0.0009990
M	0.0136676	0.1738902	0.8761239
P	0.3542345	3.6404221	0.0009990

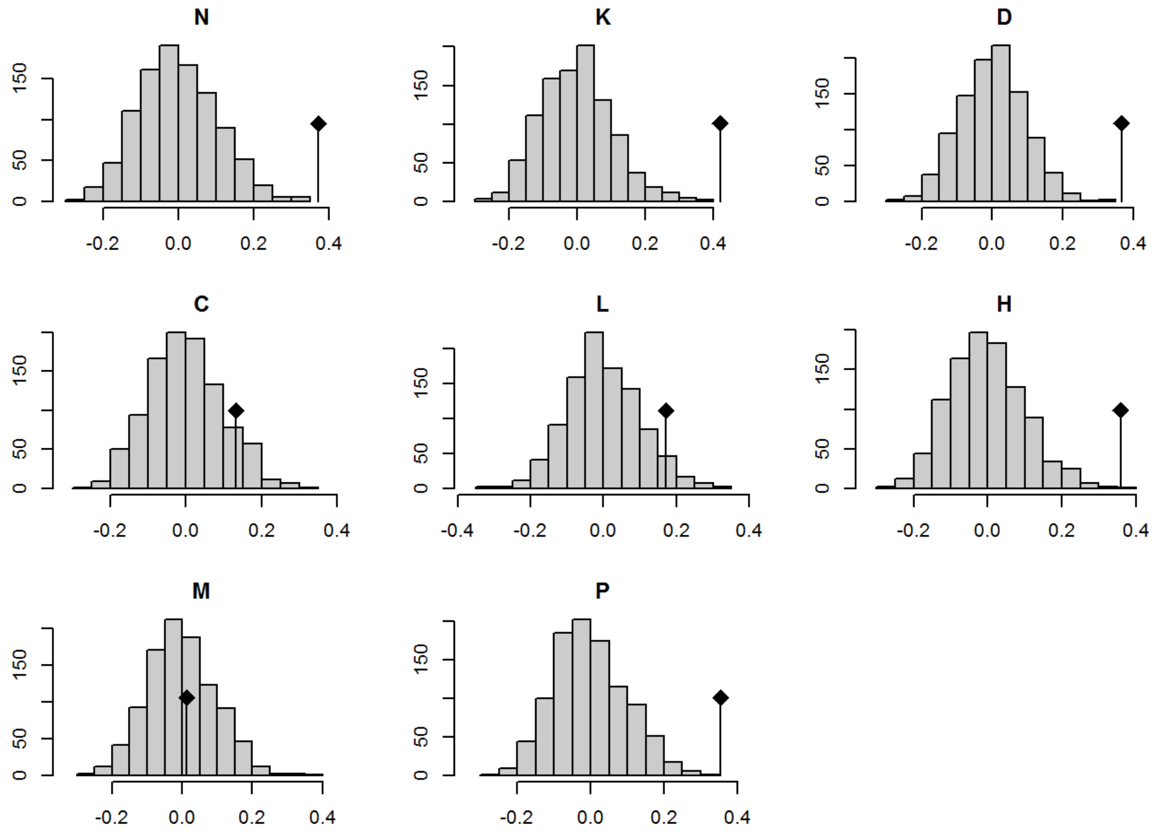

Figure A1 Abouheif's test results (graphically)

Table A2 Blomberg's test results

	K	Observed variance	Expected variance	p-value
N	0.9949704	1.0183979	1.6611474	0.0009990
K	0.8787754	15.1372167	26.6005918	0.0009990
D	1.4785578	0.0000004	0.0000007	0.0009990
C	0.5590563	0.0000347	0.0000364	0.3466533
L	0.7615715	0.0000943	0.0001109	0.0719281
H	1.1083660	0.0000750	0.0001315	0.0009990
M	0.5257506	0.0118682	0.0124085	0.3336663
P	0.6403939	0.0000112	0.0000143	0.0119880

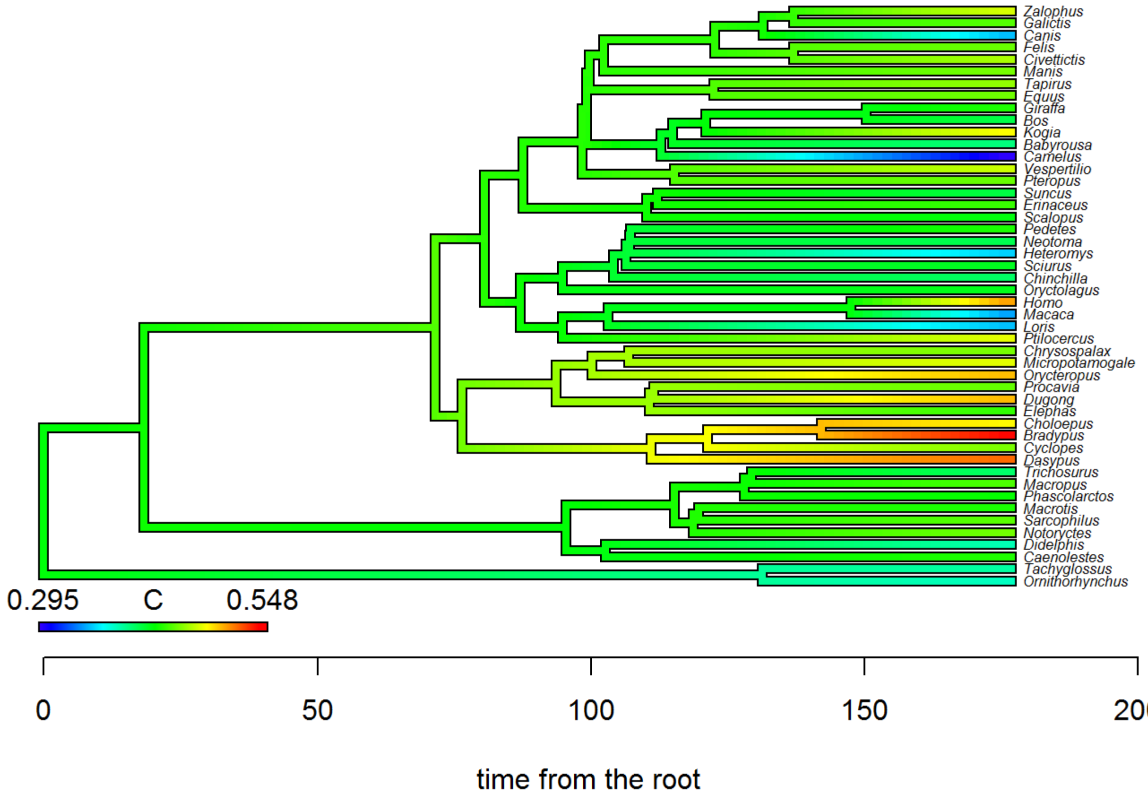

Figure A2 Network average clustering coefficient (C) mapped on the phylogeny

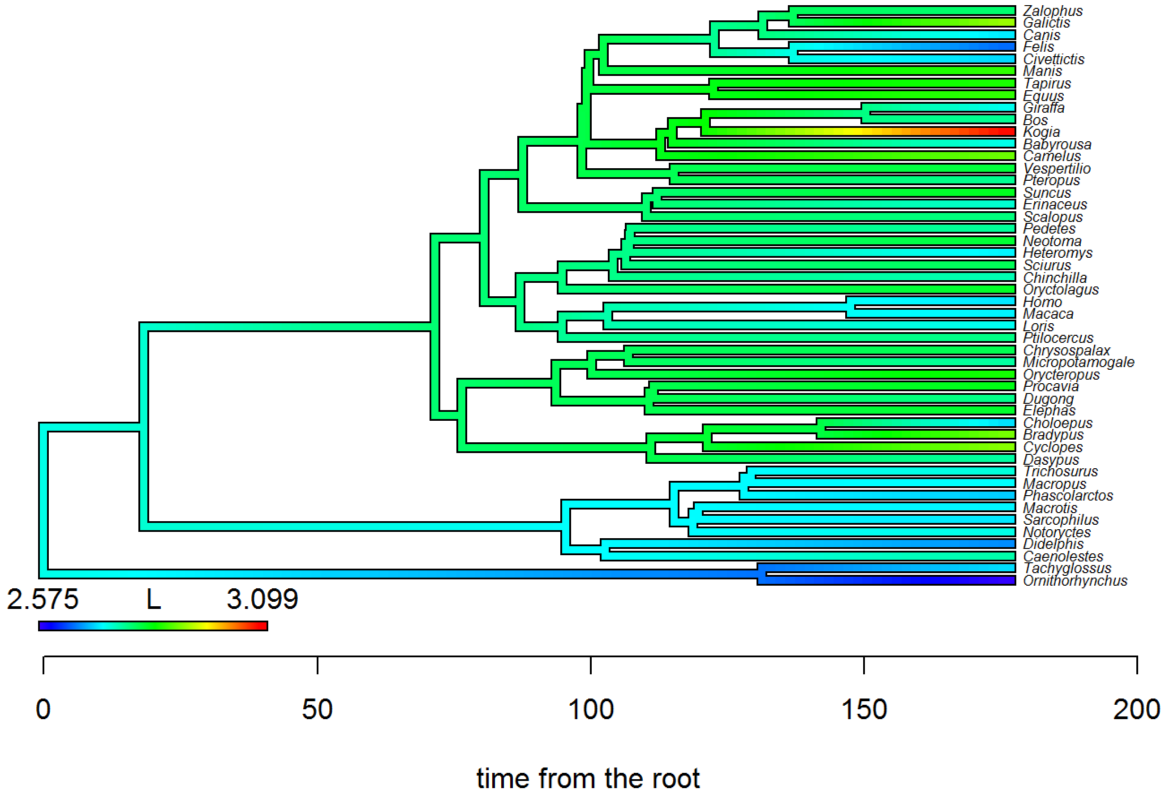

Figure A3 Network average shortest path length (L) mapped on the phylogeny

Table A3 Results of model fitting on trait evolution of network parameters. BM Brownian Motion model; EB Early Burst model; OU Ornbeck-Uhlenstein model.

	**log-likelihood**	**AIC**	**AICc**	**ΔAIC**	**AIC weights**
BM	-282.834	635.6676	644.0676	0	0.7701
EB	-282.834	637.6676	646.5773	2.51	0.2196
OU	-248.521	623.041	652.688	8.62	0.0103

Relative Variability of Network Parameters

Table A4 Results of pair-wise asymptotic test for the equality of coefficients of variation for network parameters. Asymtotic test values and Bonferroni corrected p-values are above and below the diagonal, respectively. P-values < 0.05 are in bold. P-values that were below < 0.05 before correction are underlined.

	**N**	**K**	**D**	**C**	**L**	**H**	**P**
**N**		4.109	3.364	1.098	49.358	6.692	31.896
**K**	<u>0.896</u>		0.039	0.981	70.573	20.115	52.179
**D**	1.000	1.000		0.632	68.541	18.569	50.154
**C**	1.000	1.000	1.000		60.267	12.830	42.080
**L**	**0.000**	**0.000**	**0.000**	**0.000**		24.213	3.067
**H**	<u>0.203</u>	**0.000**	**0.000**	**0.007**	**0.000**		11.040
**P**	**0.000**	**0.000**	**0.000**	**0.000**	1.000	**0.019**	

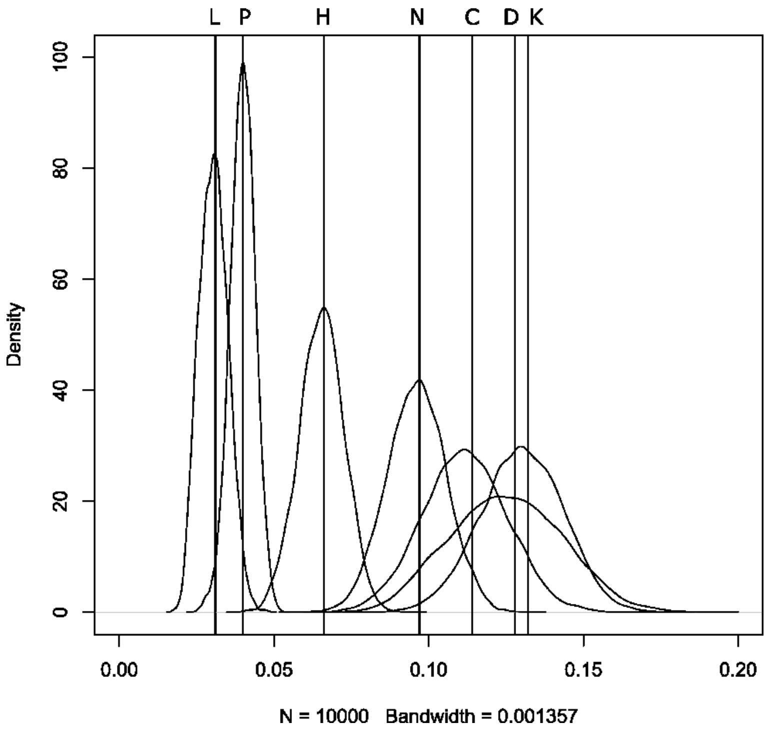

Figure A4 Distribution of coefficients of variation from the network parameters

Tabel A5 Results of the phylomorphospace analysis

	**PC1**	**PC2**	**PC3**	**PC4**	**PC5**	**PC6**	**PC7**
N	0.562086	-0.010337	-0.025384	-0.005640	0.128096	0.387099	0.719043
K	0.458417	0.366197	0.026895	0.218483	0.614035	0.034877	-0.478585
D	-0.420547	0.439927	0.045545	0.245556	0.374967	-0.421678	0.498818
C	-0.015528	-0.326727	0.709268	0.618124	-0.045404	0.076082	0.00446
L	0.011537	-0.674752	0.045538	-0.326746	0.569983	-0.328293	0.055522
H	0.544624	0.052081	0.068548	0.02648	-0.372834	-0.744483	0.044851
P	0.008244	-0.327877	-0.697656	0.634345	-0.002099	-0.057499	0.000517
Standard deviation	1.7606	1.3709	0.9963	0.8838	0.38787	0.29842	0.08817
Proportion of variance	0.4428	0.2685	0.1418	0.1116	0.02149	0.01272	0.00111
Cumulative proportion	0.4428	0.7113	0.8531	0.9647	0.98617	0.99889	100

Community structure and modularity

Table A6 Summary of the modularity analysis

	Modules (M)	Q value	Q expected error	Parcellation (P)
Babyrousa babyrussa	6	0.3157534	0.0279366	0.7842649
Bos taurus	5	0.3570920	0.0289084	0.7690329
Bradypus tridactylus	5	0.4107779	0.0279242	0.7828099
Caenolestes fuliginosus	5	0.3289442	0.0257673	0.7704918
Camelus bactrianus	5	0.4276531	0.0329551	0.7714844
Canis lupus	5	0.3742136	0.0271834	0.7837927
Chinchilla lanigera	7	0.3234277	0.0287324	0.8237311
Choloepus didactylus	6	0.3588158	0.0328270	0.7876543
Chrysospalax trevelyani	4	0.3576601	0.0278696	0.7240115
Civettictis civetta	5	0.3340317	0.0273956	0.7535437
Cyclopes didactylus	7	0.3671672	0.0324112	0.8196615
Dasypus novemcinctus	7	0.3461030	0.0313319	0.8106371
Didelphis virginiana	5	0.3149897	0.0264548	0.7513717
Dugong dugon	6	0.3470239	0.0286939	0.7953008
Elephas maximus	5	0.4066538	0.0298776	0.7760331
Equus caballus	6	0.4095849	0.0277378	0.7855099
Erinaceus europaeus	4	0.3202525	0.0290442	0.7337278
Felis silvestris	5	0.3157275	0.0287367	0.7840513
Galictis cuja	5	0.4156813	0.0280701	0.7795174
Giraffa camelopardalis	5	0.3596841	0.0298892	0.7933428
Heteromys desmarestianus	6	0.3135210	0.0311058	0.7949219
Homo sapiens	5	0.3755961	0.0283249	0.7930143
Kogia breviceps	7	0.4761160	0.0329074	0.8246528
Loris tardigradus	6	0.3302630	0.0281179	0.8077870
Macaca mulatta	7	0.3247553	0.0273871	0.8118785
Macropus rufus	4	0.3515929	0.0265834	0.7252870
Macrotis lagotis	7	0.3000523	0.0258054	0.8120833
Manis pentadactyla	5	0.3460312	0.0299786	0.7905107
Micropotamogale ruwenzorii	5	0.3412755	0.0279693	0.7844650
Neotoma fuscipes	5	0.3575422	0.0275174	0.7406944
Notoryctes typhlops	6	0.3146984	0.0271449	0.8051658
Ornithorhynchus anatinus	5	0.3185768	0.0301478	0.7638889
Orycteropus afer	5	0.4051552	0.0301417	0.7850211
Oryctolagus cuniculus	5	0.3527229	0.0277139	0.7863478
Pedetes capensis	6	0.3380556	0.0284042	0.8018176
Phascolarctos cinereus	5	0.3127772	0.0248347	0.7102606
Procavia capensis	6	0.3436721	0.0283184	0.8145180
Pteropus vampyrus	5	0.3740459	0.0302108	0.7318053
Ptilocercus lowii	4	0.3660936	0.0273621	0.7160665

	Modules (M)	Q value	Q expected error	Parcellation (P)
Sarcophilus harrisii	7	0.3197995	0.0260749	0.7789428
Scalopus aquaticus	4	0.3601520	0.0286475	0.7282236
Sciurus vulgaris	5	0.3776944	0.0258325	0.7591716
Suncus murinus	6	0.3542893	0.0293603	0.7527737
Tachyglossus aculeatus	6	0.3371228	0.0321765	0.7916955
Tapirus indicus	6	0.3817704	0.0290636	0.8054786
Trichosurus vulpecula	4	0.3307777	0.0273543	0.7250231
Vespertilio murinus	4	0.3902735	0.0311857	0.7285156
Zalophus californianus	6	0.4106255	0.0269593	0.7848319

Table A7 Connectivity modules identified for *Babyrousa babyrussa*.

ID	p-value	Elements
1	0.07324	C2, C3, C4, iliocostalis.cervicis.left, iliocostalis.cervicis.right, longus.capitis.left, longus.capitis.right, scalenus.medius.left, scalenus.medius.right, serratus.ventralis.cervicis.right, interspinalis.1, interspinalis.2, interspinalis.3, multifidius.submultifidius.1.left, multifidius.submultifidius.2.left, multifidius.submultifidius.3.left, intertransversarius.dorsalis.2.left, intertransversarius.dorsalis.3.left, multifidius.submultifidius.1.right, multifidius.submultifidius.2.right, multifidius.submultifidius.3.right, intertransversarius.dorsalis.2.right, intertransversarius.dorsalis.3.right, intertransversarius.ventralis.longus.left, intertransversarius.ventralis.longus.right
2	0.00017	C5, C6, C7, thoracic.spine, complexus.left, complexus.right, longissimus.capitis.left, longissimus.capitis.right, longissimus.cervicis.left, longissimus.cervicis.right, longus.colli.left, longus.colli.right, rhomboideus.cervicis.left, rhomboideus.cervicis.right, scalenus.ventralis.left, scalenus.ventralis.right, spinalis.cervicis.left, spinalis.cervicis.right, interspinalis.4, interspinalis.5, interspinalis.6, multifidius.submultifidius.4.left, multifidius.submultifidius.5.left, multifidius.submultifidius.6.left, intertransversarius.dorsalis.4.left, intertransversarius.dorsalis.5.left, intertransversarius.dorsalis.6.left, multifidius.submultifidius.4.right, multifidius.submultifidius.5.right, multifidius.submultifidius.6.right, intertransversarius.dorsalis.4.right, intertransversarius.dorsalis.5.right, intertransversarius.dorsalis.6.right
3	0.00149	cranium, C1, humerus.left, biventer.cervicis.left, biventer.cervicis.right, cephalohumeralis.left, cleidomastoideus.left, obliquus.capitis.caudalis.left, obliquus.capitis.caudalis.right, obliquus.capitis.cranialis.left, obliquus.capitis.cranialis.right, rectus.capitis.dorsalis.major.left, rectus.capitis.dorsalis.major.right, rectus.capitis.dorsalis.minor.left, rectus.capitis.dorsalis.minor.right, rectus.capitis.lateralis.left, rectus.capitis.lateralis.right, rectus.capitis.ventralis.left, rectus.capitis.ventralis.right, splenius.left, splenius.right
4	0.78916	scapula.left, atlantoscapularis.ventralis.left, omohyoideus.left, rhomboideus.capitis.left, serratus.ventralis.cervicis.left, trapezius.left
5	0.40832	scapula.right, humerus.right, atlantoscapularis.ventralis.right, cephalohumeralis.right, cleidomastoideus.right, omohyoideus.right, rhomboideus.capitis.right, trapezius.right
6	0.04049	sternum, hyoid, thyroid, ribs.left, ribs.right, scalenus.dorsalis.left, scalenus.dorsalis.right, sternohyoideus.left, sternohyoideus.right, sternomastoideus.left, sternomastoideus.right, sternothyroideus.left, sternothyroideus.right

Table A8 Connectivity modules identified for *Bos taurus*.

ID	p-value	Elements
1	0.15196	scapula.left, scapula.right, nuchal.ligament, omotransversarius.left, omotransversarius.right, rhomboideus.cervicis.left, rhomboideus.cervicis.right, trapezius.left, trapezius.right
2	4e-05	cranium, C1, humerus.left, humerus.right, cleidomastoideus.left, cleidomastoideus.right, cleidooccipitalis.left, cleidooccipitalis.right, obliquus.capitis.caudalis.left, obliquus.capitis.caudalis.right, obliquus.capitis.cranialis.left, obliquus.capitis.cranialis.right, rectus.capitis.dorsalis.major.left, rectus.capitis.dorsalis.major.right, rectus.capitis.dorsalis.minor.left, rectus.capitis.dorsalis.minor.right, rectus.capitis.lateralis.left, rectus.capitis.lateralis.right, rectus.capitis.ventralis.left, rectus.capitis.ventralis.right, splenius.capitis.left, splenius.capitis.right, splenius.cervicis.left, splenius.cervicis.right
3	7e-05	C5, C6, C7, thoracic.spine, iliocostalis.cervicis.left, iliocostalis.cervicis.right, longissimus.capitis.left, longissimus.capitis.right, longissimus.cervicis.left, longissimus.cervicis.right, longus.colli.right, scalenus.medius.left, scalenus.medius.right, scalenus.ventralis.left, scalenus.ventralis.right, semispinalis.capitis.left, semispinalis.capitis.right, semispinalis.cervicis.left, semispinalis.cervicis.right, interspinalis.4, interspinalis.5, interspinalis.6, multifidius.submultifidius.4.left, multifidius.submultifidius.5.left, multifidius.submultifidius.6.left, intertransversarius.dorsalis.4.left, intertransversarius.dorsalis.5.left, intertransversarius.dorsalis.6.left, multifidius.submultifidius.4.right, multifidius.submultifidius.5.right, multifidius.submultifidius.6.right, intertransversarius.dorsalis.4.right, intertransversarius.dorsalis.5.right, intertransversarius.dorsalis.6.right
4	0.00266	mandible, sternum, hyoid, thyroid, ribs.left, ribs.right, scalenus.dorsalis.left, scalenus.dorsalis.right, sternohyoideus.left, sternohyoideus.right, sternomandibularis.left, sternomandibularis.right, sternomastoideus.left, sternomastoideus.right, sternothyroideus.left, sternothyroideus.right
5	0.04034	C2, C3, C4, longus.capitis.left, longus.capitis.right, longus.colli.left, omohyoideus.left, omohyoideus.right, serratus.ventralis.cervicis.left, serratus.ventralis.cervicis.right, interspinalis.1, interspinalis.2, interspinalis.3, multifidius.submultifidius.1.left, multifidius.submultifidius.2.left, multifidius.submultifidius.3.left, intertransversarius.dorsalis.2.left, intertransversarius.dorsalis.3.left, intertransversarius.ventralis.longus.left, intertransversarius.ventralis.longus.right, multifidius.submultifidius.1.right, multifidius.submultifidius.2.right, multifidius.submultifidius.3.right, intertransversarius.dorsalis.2.right, intertransversarius.dorsalis.3.right

Table A9 Connectivity modules identified for *Bradypus tridactylus*.

ID	p-value	Elements
1	0.00044	C2, C3, C4, C5, longissimus.capitis.left, longissimus.capitis.right, longissimus.cervicis.left, longissimus.cervicis.right, scalenus.anticus.left, scalenus.anticus.right, semispinalis.capitis.left, semispinalis.capitis.right, semispinalis.cervicis.left, semispinalis.cervicis.right, splenius.capitis.left, splenius.capitis.right, splenius.cervicis.left, splenius.cervicis.right, interspinalis.1, interspinalis.2, interspinalis.3, intertransversarius.dorsalis.1.left, intertransversarius.dorsalis.2.left, intertransversarius.dorsalis.3.left, intertransversarius.ventralis.1.left, intertransversarius.ventralis.2.left, intertransversarius.ventralis.3.left, intertransversarius.dorsalis.1.right, intertransversarius.dorsalis.2.right, intertransversarius.dorsalis.3.right, intertransversarius.ventralis.1.right, intertransversarius.ventralis.2.right, intertransversarius.ventralis.3.right
2	0.00103	cranium, C1, cleido.mastoideus.right, obliquus.capitis.caudalis.left, obliquus.capitis.caudalis.right, obliquus.capitis.cranialis.left, obliquus.capitis.cranialis.right, rectus.capitis.dorsalis.major.left, rectus.capitis.dorsalis.major.right, rectus.capitis.dorsalis.minor.left, rectus.capitis.dorsalis.minor.right, rectus.capitis.lateralis.left, rectus.capitis.lateralis.right, rectus.capitis.ventralis.left, rectus.capitis.ventralis.right
3	0.00125	clavicle.left, clavicle.right, sternum, hyoid, thyroid, ribs.left, ribs.right, clavotrapezius.left, clavotrapezius.right, cleido.mastoideus.left, sternocleidomastoideus.left, sternocleidomastoideus.right, sternohyoid.left, sternohyoid.right, sternothyroideus.left, sternothyroideus.right
4	0.01892	C6, C7, iliocostalis.cervicis.left, iliocostalis.cervicis.right, longus.capitis.left, longus.capitis.right, scalenus.posticus.left, scalenus.posticus.right, interspinalis.4, interspinalis.5, interspinalis.6, intertransversarius.dorsalis.4.left, intertransversarius.dorsalis.5.left, intertransversarius.dorsalis.6.left, intertransversarius.ventralis.4.left, intertransversarius.ventralis.5.left, intertransversarius.ventralis.6.left, intertransversarius.dorsalis.4.right, intertransversarius.dorsalis.5.right, intertransversarius.dorsalis.6.right, intertransversarius.ventralis.4.right, intertransversarius.ventralis.5.right, intertransversarius.ventralis.6.right
5	0.00244	C8, C9, thoracic.spine, scapula.left, scapula.right, longus.colli.left, longus.colli.right, rhomboideus.cervicis.left, rhomboideus.cervicis.right, serratus.ventralis.cervicis.left, serratus.ventralis.cervicis.right, trapezius.left, trapezius.right, interspinalis.7, interspinalis.8, intertransversarius.dorsalis.7.left, intertransversarius.dorsalis.8.left, intertransversarius.ventralis.7.left, intertransversarius.ventralis.8.left, intertransversarius.dorsalis.7.right, intertransversarius.dorsalis.8.right, intertransversarius.ventralis.7.right, intertransversarius.ventralis.8.right

Table A10 Connectivity modules identified for *Caenolestes fuliginosus*.

ID	p-value	Elements
1	0.02453	sternum, hyoid, thyroid, ribs.right, longus.colli.right, omohyoideus.left, omohyoideus.right, sternohyoideus.left, sternohyoideus.right, sternomastoideus.left, sternomastoideus.right, sternothyroideus.left, sternothyroideus.right
2	0.53254	C2, complexus.left, obliquus.capitis.caudalis.left, obliquus.capitis.caudalis.right, rectus.capitis.dorsalis.major.left, rectus.capitis.dorsalis.major.right, rectus.capitis.dorsalis.superficialis.left, rectus.capitis.dorsalis.superficialis.right, interspinalis.1, intertransversarii.dorsalis.1.left, intertransversarii.ventralis.1.left, intertransversarii.dorsalis.1.right, intertransversarii.ventralis.1.right
3	0.04481	C6, C7, thoracic.spine, ribs.left, iliocostalis.dorsi.left, iliocostalis.dorsi.right, longissimus.capitis.left, longissimus.capitis.right, longissimus.cervicis.left, longissimus.cervicis.right, longus.colli.left, semispinalis.cervicis.left, semispinalis.cervicis.right, splenius.left, splenius.right, interspinalis.5, interspinalis.6, multifidius.submultifidius.5.left, multifidius.submultifidius.6.left, intertransversarii.dorsalis.4.left, intertransversarii.dorsalis.5.left, intertransversarii.dorsalis.6.left, intertransversarii.ventralis.5.left, intertransversarii.ventralis.6.left, spinalis.cervicis.left, multifidius.submultifidius.5.right, multifidius.submultifidius.6.right, intertransversarii.dorsalis.5.right, intertransversarii.dorsalis.6.right, intertransversarii.ventralis.4.right, intertransversarii.ventralis.5.right, intertransversarii.ventralis.6.right, spinalis.cervicis.right
4	1e-04	cranium, C1, clavicle.left, clavicle.right, scapula.left, scapula.right, acromiotrapezius.left, atlantoacromialis.left, atlantoacromialis.right, atlantoscapularis.left, atlantoscapularis.right, biventer.cervicis.left, biventer.cervicis.right, cleidomastoideus.left, cleidomastoideus.right, cleidooccipitalis.left, cleidooccipitalis.right, complexus.right, obliquus.capitis.cranialis.left, obliquus.capitis.cranialis.right, rectus.capitis.dorsalis.minor.left, rectus.capitis.dorsalis.minor.right, rectus.capitis.lateralis.left, rectus.capitis.lateralis.right, rectus.capitis.ventralis.left, rectus.capitis.ventralis.right, rhomboideus.capitis.left, rhomboideus.capitis.right, rhomboideus.major.left, rhomboideus.major.right
5	0.00011	C3, C4, C5, acromiotrapezius.right, levator.scapulae.left, levator.scapulae.right, longus.capitis.left, longus.capitis.right, scalenus.anticus.left, scalenus.anticus.right, scalenus.medius.left, scalenus.medius.right, interspinalis.2, interspinalis.3, interspinalis.4, multifidius.submultifidius.1.left, multifidius.submultifidius.2.left, multifidius.submultifidius.3.left, multifidius.submultifidius.4.left, intertransversarii.dorsalis.2.left, intertransversarii.dorsalis.3.left, intertransversarii.ventralis.2.left, intertransversarii.ventralis.3.left, intertransversarii.ventralis.4.left, multifidius.submultifidius.1.right, multifidius.submultifidius.2.right, multifidius.submultifidius.3.right, multifidius.submultifidius.4.right, intertransversarii.dorsalis.2.right, intertransversarii.dorsalis.3.right, intertransversarii.dorsalis.4.right, intertransversarii.ventralis.2.right, intertransversarii.ventralis.3.right

Table A11 Connectivity modules identified for *Camelus bactrianus*.

ID	p-value	Elements
1	4e-05	C5, C6, C7, thoracic.spine, ribs.left, ribs.right, intertransversarius.dorsalis.4.left, intertransversarius.dorsalis.5.left, intertransversarius.dorsalis.6.left, levator.scapulae.left, longissimus.cervicis.left, longus.colli.left, multifius.4.left, scalenus.primocostal.left, scalenus.supracostal.left, spinalis.cervicis.left, intertransversarius.dorsalis.4.right, intertransversarius.dorsalis.5.right, intertransversarius.dorsalis.6.right, levator.scapulae.right, longissimus.cervicis.right, longus.colli.right, multifius.4.right, scalenus.primocostal.right, scalenus.supracostal.right, spinalis.cervicis.right
2	0.00168	scapula.left, scapula.right, humerus.left, humerus.right, nuchal.ligament, mastohumeralis.left, omotransversalis.left, rhomboideus.cervicis.left, mastohumeralis.right, omotransversalis.right, rhomboideus.cervicis.right
3	0.00395	C2, C3, C4, complexus.minor.left, intertransversarius.dorsalis.1.left, intertransversarius.dorsalis.2.left, intertransversarius.dorsalis.3.left, intertransversarius.ventralis.brevis.1.left, intertransversarius.ventralis.brevis.2.left, intertransversarius.ventralis.brevis.3.left, intertransversarius.ventralis.longus.left, longus.capitis.left, multifius.1.left, multifius.2.left, multifius.3.left, omohyoideus.left, complexus.minor.right, intertransversarius.dorsalis.1.right, intertransversarius.dorsalis.2.right, intertransversarius.dorsalis.3.right, intertransversarius.ventralis.brevis.1.right, intertransversarius.ventralis.brevis.2.right, intertransversarius.ventralis.brevis.3.right, intertransversarius.ventralis.longus.right, longus.capitis.right, multifius.1.right, multifius.2.right, multifius.3.right, omohyoideus.right
4	0.00075	mandible, sternum, hyoid, thyroid, sternohyoideus.left, sternomatoideus.left, sternomaxillaris.left, sternothyroideus.left, sternohyoideus.right, sternomatoideus.right, sternomaxillaris.right, sternothyroideus.right
5	0.00023	cranium, C1, complexus.major.left, longus.atlantis.left, obliquus.capitis.caudalis.left, obliquus.capitis.cranialis.left, rectus.capitis.dorsalis.major.left, rectus.capitis.dorsalis.minor.left, rectus.capitis.lateralis.left, rectus.capitis.ventralis.left, complexus.major.right, longus.atlantis.right, obliquus.capitis.caudalis.right, obliquus.capitis.cranialis.right, rectus.capitis.dorsalis.major.right, rectus.capitis.dorsalis.minor.right, rectus.capitis.lateralis.right, rectus.capitis.ventralis.right

Table A12 Connectivity modules identified for *Canis lupus*.

ID	p-value	Elements
1	0.00152	sternum, hyoid, thyroid, ribs.left, ribs.right, scalenus.supracostalis.left, scalenus.supracostalis.right, sternohyoideus.left, sternohyoideus.right, sternomastoideus.left, sternomastoideus.right, sternooccipitalis.left, sternooccipitalis.right, sternothyroideus.left, sternothyroideus.right
2	0.00211	C2, C3, C4, longus.capitis.left, longus.capitis.right, longus.colli.left, longus.colli.right, semispinalis.cervicis.left, semispinalis.cervicis.right, spinalis.cervicis.left, spinalis.cervicis.right, interspinalis.1, interspinalis.2, interspinalis.3, multifidius.submultifidius.1.left, multifidius.submultifidius.2.left, multifidius.submultifidius.3.left, intertransversarii.dorsalis.1.left, intertransversarii.dorsalis.2.left, intertransversarii.dorsalis.3.left, intertransversarii.ventralis.left, multifidius.submultifidius.1.right, multifidius.submultifidius.2.right, multifidius.submultifidius.3.right, intertransversarii.dorsalis.1.right, intertransversarii.dorsalis.2.right, intertransversarii.dorsalis.3.right, intertransversarii.ventralis.right, intertransversarius.intermedius.1.right, intertransversarius.intermedius.2.right, intertransversarius.intermedius.3.right, intertransversarius.intermedius.1.left, intertransversarius.intermedius.2.left, intertransversarius.intermedius.3.left
3	1e-04	cranium, C1, scapula.left, humerus.left, biventer.cervicis.left, cleidocervicalis.left, cleidomastoideus.left, levator.claviculae.left, obliquus.capitis.caudalis.left, obliquus.capitis.caudalis.right, obliquus.capitis.cranialis.left, obliquus.capitis.cranialis.right, rectus.capitis.dorsalis.intermedius.left, rectus.capitis.dorsalis.intermedius.right, rectus.capitis.dorsalis.major.left, rectus.capitis.dorsalis.major.right, rectus.capitis.dorsalis.minor.left, rectus.capitis.dorsalis.minor.right, rectus.capitis.lateralis.left, rectus.capitis.lateralis.right, rectus.capitis.ventralis.left, rectus.capitis.ventralis.right, rhomboideus.capitis.left
4	0.0024	ligamentum.nuchae, scapula.right, thoracic.spine, humerus.right, biventer.cervicis.right, cleidocervicalis.right, cleidomastoideus.right, levator.claviculae.right, rhomboideus.capitis.right, rhomboideus.cervicis.left, rhomboideus.cervicis.right, splenius.left, splenius.right, trapezius.left, trapezius.right, interspinalis.6, intertransversarii.dorsalis.5.left, intertransversarii.dorsalis.6.left, intertransversarii.dorsalis.5.right, intertransversarii.dorsalis.6.right
5	0.00523	C5, C6, C7, complexus.left, complexus.right, longissimus.atlantis.left, longissimus.atlantis.right, longissimus.capitis.left, longissimus.capitis.right, longissimus.cervicis.left, longissimus.cervicis.right, scalenus.primae.costae.left, scalenus.primae.costae.right, serratus.ventralis.cervicis.left, serratus.ventralis.cervicis.right, interspinalis.4, interspinalis.5, multifidius.submultifidius.4.left, multifidius.submultifidius.5.left, multifidius.submultifidius.6.left, intertransversarii.dorsalis.4.left, multifidius.submultifidius.4.right, multifidius.submultifidius.5.right, multifidius.submultifidius.6.right, intertransversarii.dorsalis.4.right, intertransversarius.intermedius.4.right, intertransversarius.intermedius.5.right, intertransversarius.intermedius.6.right, intertransversarius.intermedius.4.left, intertransversarius.intermedius.5.left, intertransversarius.intermedius.6.left

Table A13 Connectivity modules identified for *Chinchilla lanigera*.

ID	p-value	Elements
1	0.91792	ribs.right, scalenus.anticus.right, scalenus.medius.right
2	0.12543	C2, C3, obliquus.capitis.caudalis.left, obliquus.capitis.caudalis.right, scalenus.medius.left, serratus.ventralis.left, splenius.left, splenius.right, multifidius.submultifidius.1.left, multifidius.submultifidius.2.left, intertransversarius.dorsalis.1.left, intertransversarius.dorsalis.2.left, intertransversarius.ventralis.1.left, intertransversarius.ventralis.2.left, multifidius.submultifidius.1.right, multifidius.submultifidius.2.right, intertransversarius.dorsalis.1.right, intertransversarius.dorsalis.2.right, intertransversarius.ventralis.1.right, intertransversarius.ventralis.2.right
3	0.9094	scapula.right, levator.claviculae.right, levator.scapulae.right, rhomboideus.capitis.right, rhomboideus.right, trapezius.right
4	0.0014	cranium, C1, scapula.left, biventer.cervicis.left, biventer.cervicis.right, levator.claviculae.left, levator.scapulae.left, obliquus.capitis.cranialis.left, obliquus.capitis.cranialis.right, rectus.capitis.dorsalis.major.left, rectus.capitis.dorsalis.major.right, rectus.capitis.dorsalis.minor.left, rectus.capitis.dorsalis.minor.right, rectus.capitis.lateralis.left, rectus.capitis.lateralis.right, rectus.capitis.ventralis.left, rectus.capitis.ventralis.right, rhomboideus.capitis.left, rhomboideus.left, trapezius.left
5	0.00026	clavicle.left, clavicle.right, sternum, hyoid, thyroid, ribs.left, cleidomastoideus.left, cleidomastoideus.right, scalenus.anticus.left, sternohyoideus.left, sternohyoideus.right, sternomastoideus.left, sternomastoideus.right, sternothyroideus.left, sternothyroideus.right
6	0.02974	C4, C5, C6, complexus.left, iliocostalis.cervicis.left, iliocostalis.cervicis.right, longissimus.cervicis.left, longissimus.cervicis.right, longus.capitis.left, longus.capitis.right, serratus.ventralis.right, spinalis.cervicis.left, spinalis.cervicis.right, splenius.capitis.left, splenius.capitis.right, multifidius.submultifidius.3.left, intertransversarius.dorsalis.3.left, intertransversarius.dorsalis.4.left, intertransversarius.ventralis.3.left, intertransversarius.ventralis.4.left, multifidius.submultifidius.3.right, intertransversarius.dorsalis.3.right, intertransversarius.dorsalis.4.right, intertransversarius.ventralis.3.right, intertransversarius.ventralis.4.right
7	0.03537	C7, thoracic.spine, complexus.right, longus.colli.left, longus.colli.right, multifidius.submultifidius.4.left, multifidius.submultifidius.5.left, multifidius.submultifidius.6.left, intertransversarius.dorsalis.5.left, intertransversarius.dorsalis.6.left, intertransversarius.ventralis.5.left, intertransversarius.ventralis.6.left, multifidius.submultifidius.4.right, multifidius.submultifidius.5.right, multifidius.submultifidius.6.right, intertransversarius.dorsalis.5.right, intertransversarius.dorsalis.6.right, intertransversarius.ventralis.5.right, intertransversarius.ventralis.6.right

Table A14 Connectivity modules identified for *Choloepus didactylus*.

ID	p-value	Elements
1	0.41149	clavicle.right, scapula.right, cleido.mastoideus.right, levator.claviculae.right, rhomboideus.capitis.right, serratus.ventralis.cervicis.right, trapezius.right
2	0.00125	cranium, C1, obliquus.capitis.caudalis.left, obliquus.capitis.caudalis.right, obliquus.capitis.cranialis.left, obliquus.capitis.cranialis.right, rectus.capitis.dorsalis.major.left, rectus.capitis.dorsalis.major.right, rectus.capitis.dorsalis.minor.left, rectus.capitis.dorsalis.minor.right, rectus.capitis.lateralis.left, rectus.capitis.lateralis.right, rectus.capitis.ventralis.left, rectus.capitis.ventralis.right
3	0.41149	clavicle.left, scapula.left, cleido.mastoideus.left, levator.claviculae.left, rhomboideus.capitis.left, serratus.ventralis.cervicis.left, trapezius.left
4	0.00211	C5, C6, thoracic.spine, iliocostalis.cervicis.left, iliocostalis.cervicis.right, longissimus.capitis.left, longissimus.capitis.right, longissimus.cervicis.left, longissimus.cervicis.right, longus.capitis.left, longus.capitis.right, rhomboideus.cervicis.left, rhomboideus.cervicis.right, semispinalis.capitis.left, interspinalis.4, interspinalis.5, intertransversarius.dorsalis.4.left, intertransversarius.dorsalis.5.left, intertransversarius.ventralis.4.left, intertransversarius.ventralis.5.left, intertransversarius.dorsalis.4.right, intertransversarius.dorsalis.5.right, intertransversarius.ventralis.4.right, intertransversarius.ventralis.5.right
5	0.07576	C2, C3, C4, longus.colli.left, longus.colli.right, scalenus.posticus.left, scalenus.posticus.right, semispinalis.capitis.right, splenius.capitis.left, splenius.capitis.right, splenius.cervicis.left, splenius.cervicis.right, interspinalis.1, interspinalis.2, interspinalis.3, intertransversarius.dorsalis.1.left, intertransversarius.dorsalis.2.left, intertransversarius.dorsalis.3.left, intertransversarius.ventralis.1.left, intertransversarius.ventralis.2.left, intertransversarius.ventralis.3.left, intertransversarius.dorsalis.1.right, intertransversarius.dorsalis.2.right, intertransversarius.dorsalis.3.right, intertransversarius.ventralis.1.right, intertransversarius.ventralis.2.right, intertransversarius.ventralis.3.right
6	0.00625	sternum, hyoid, thyroid, ribs.left, ribs.right, sternohyoid.left, sternohyoid.right, sternomastoideus.left, sternomastoideus.right, sternothyroideus.left, sternothyroideus.right

Table A15 Connectivity modules identified for *Chrysospalax trevelyani*.

ID	p-value	Elements
1	0.01538	sternum, hyoid, thyroid, ribs.left, iliocostalis.cervicis.left, omohyoid.left, omohyoid.right, sternohyoid.left, sternohyoid.right, sternomastoideus.left, sternomastoideus.right, sternothyroid.left, sternothyroid.right
2	0	cranium, C1, scapula.left, scapula.right, clavicula.left, clavicula.right, biventer.cervicis.left, biventer.cervicis.right, cleidomastoid.left, cleidomastoid.right, cleidooccipitalis.left, cleidooccipitalis.right, levator.claviculae.left, levator.claviculae.right, levator.scapulae.left, levator.scapulae.right, longus.capitis.left, longus.capitis.right, obliquus.capitis.caudalis.left, obliquus.capitis.caudalis.right, obliquus.capitis.cranialis.left, obliquus.capitis.cranialis.right, rectus.capitis.dorsalis.major.left, rectus.capitis.dorsalis.major.right, rectus.capitis.dorsalis.minor.left, rectus.capitis.dorsalis.minor.right, rectus.capitis.lateralis.left, rectus.capitis.lateralis.right, rectus.capitis.lateralis.right.1, rectus.capitis.ventralis.left, rhomboideus.capitis.left, rhomboideus.capitis.right
3	0.00036	C2, C3, C4, C5, complexus.left, complexus.right, longissimus.cervicis.left, longissimus.cervicis.right, scalenus.ventralis.left, scalenus.ventralis.right, serratus.ventralis.cervicis.left, serratus.ventralis.cervicis.right, trapezius.left, trapezius.right, interspinalis.1, interspinalis.2, interspinalis.3, multifidius.submultifidius.1.left, multifidius.submultifidius.2.left, multifidius.submultifidius.3.left, multifidius.submultifidius.4.left, intertransversarius.dorsalis.1.left, intertransversarius.dorsalis.2.left, intertransversarius.dorsalis.3.left, intertransversarius.ventralis.1.left, intertransversarius.ventralis.2.left, intertransversarius.ventralis.3.left, multifidius.submultifidius.1.right, multifidius.submultifidius.2.right, multifidius.submultifidius.3.right, multifidius.submultifidius.4.right, intertransversarius.dorsalis.1.right, intertransversarius.dorsalis.2.right, intertransversarius.dorsalis.3.right, intertransversarius.ventralis.1.right, intertransversarius.ventralis.2.right, intertransversarius.ventralis.3.right
4	0.00338	C6, C7, thoracic.spine, ribs.right, iliocostalis.cervicis.right, longissimus.capitis.left, longissimus.capitis.right, rhomboideus.cervicis.left, rhomboideus.cervicis.right, splenius.left, splenius.right, interspinalis.4, interspinalis.5, interspinalis.6, multifidius.submultifidius.5.left, multifidius.submultifidius.6.left, intertransversarius.dorsalis.4.left, intertransversarius.dorsalis.5.left, intertransversarius.dorsalis.6.left, intertransversarius.ventralis.4.left, intertransversarius.ventralis.5.left, intertransversarius.ventralis.6.left, multifidius.submultifidius.5.right, multifidius.submultifidius.6.right, intertransversarius.dorsalis.4.right, intertransversarius.dorsalis.5.right, intertransversarius.dorsalis.6.right, intertransversarius.ventralis.4.right, intertransversarius.ventralis.5.right, intertransversarius.ventralis.6.right

Table A16 Connectivity modules identified for *Civettictis civetta*.

ID	p-value	Elements
1	0.00017	C5, C6, C7, thoracic.spine, complexus.left, complexus.right, longissimus.capitis.left, longissimus.capitis.right, longissimus.cervicis.left, longissimus.cervicis.right, longus.colli.right, rhomboideus.cervicis.left, semispinalis.cervicis.left, semispinalis.cervicis.right, serratus.ventralis.cervicis.left, spinalis.cervicis.left, spinalis.cervicis.right, splenius.cervicis.left, splenius.cervicis.rigth, trapezius.left, interspinalis.4, interspinalis.5, multifidius.submultifidius.4.left, multifidius.submultifidius.5.left, intertransversarii.dorsalis.5.left, intertransversarii.dorsalis.6.left, intertransversarii.dorsalis.7.left, multifidius.submultifidius.4.right, multifidius.submultifidius.5.right, intertransversarii.dorsalis.5.right, intertransversarii.dorsalis.6.right, intertransversarii.dorsalis.7.right, intertransversarius.intermedius.4.right, intertransversarius.intermedius.4.left
2	0.95091	scapula.right, levator.claviculae.right, rhomboideus.cervicis.right, serratus.ventralis.cervicis.right, trapezius.right
3	0.00051	C2, C3, C4, longus.capitis.left, longus.capitis.right, longus.colli.left, interspinalis.1, interspinalis.2, interspinalis.3, multifidius.submultifidius.1.left, multifidius.submultifidius.2.left, multifidius.submultifidius.3.left, intertransversarii.dorsalis.2.left, intertransversarii.dorsalis.3.left, intertransversarii.dorsalis.4.left, intertransversarii.ventralis.left, multifidius.submultifidius.1.right, multifidius.submultifidius.2.right, multifidius.submultifidius.3.right, intertransversarii.dorsalis.2.right, intertransversarii.dorsalis.3.right, intertransversarii.dorsalis.4.right, intertransversarii.ventralis.right, intertransversarius.intermedius.1.right, intertransversarius.intermedius.2.right, intertransversarius.intermedius.3.right, intertransversarius.intermedius.1.left, intertransversarius.intermedius.2.left, intertransversarius.intermedius.3.left
4	0.00086	sternum, hyoid, thyroid, ribs.left, ribs.right, scalenus.primae.costae.left, scalenus.primae.costae.right, scalenus.supracostalis.left, scalenus.supracostalis.right, sternohyoideus.left, sternohyoideus.right, sternomastoideus.left, sternomastoideus.right, sternothyroideus.left, sternothyroideus.right
5	4e-05	cranium, C1, scapula.left, humerus.left, humerus.right, biventer.cervicis.left, biventer.cervicis.right, cleidocervicalis.left, cleidocervicalis.right, cleidomastoideus.left, cleidomastoideus.right, levator.claviculae.left, obliquus.capitis.caudalis.left, obliquus.capitis.caudalis.right, obliquus.capitis.cranialis.left, obliquus.capitis.cranialis.right, rectus.capitis.dorsalis.intermedius.left, rectus.capitis.dorsalis.intermedius.right, rectus.capitis.dorsalis.major.left, rectus.capitis.dorsalis.major.right, rectus.capitis.dorsalis.minor.left, rectus.capitis.dorsalis.minor.right, rectus.capitis.lateralis.left, rectus.capitis.lateralis.right, rectus.capitis.ventralis.left, rectus.capitis.ventralis.right, splenius.capitis.left, splenius.capitis.right, intertransversarii.dorsalis.1.left, intertransversarii.dorsalis.1.right

Table A17 Connectivity modules identified for *Cyclopes didactylus*.

ID	p-value	Elements
1	0.00077	cranium, C1, longissimus.capitis.left, longissimus.capitis.right, obliquus.capitis.caudalis.left, obliquus.capitis.caudalis.right, obliquus.capitis.cranialis.left, obliquus.capitis.cranialis.right, rectus.capitis.dorsalis.major.left, rectus.capitis.dorsalis.major.right, rectus.capitis.dorsalis.minor.left, rectus.capitis.dorsalis.minor.right, rectus.capitis.lateralis.left, rectus.capitis.lateralis.right, rectus.capitis.ventralis.left, rectus.capitis.ventralis.right
2	0.07221	C2, C3, C4, thoracic.spine, longissimus.cervicis.left, longissimus.cervicis.right, longus.capitis.right, longus.colli.left, longus.colli.right, rhomboideus.cervicis.left, rhomboideus.cervicis.right, semispinalis.capitis.left, semispinalis.capitis.right, splenius.capitis.left, splenius.capitis.right, interspinalis.1, interspinalis.2, intertransversarius.dorsalis.1.left, intertransversarius.dorsalis.2.left, intertransversarius.ventralis.1.left, intertransversarius.ventralis.2.left, intertransversarius.dorsalis.1.right, intertransversarius.dorsalis.2.right, intertransversarius.ventralis.1.right, intertransversarius.ventralis.2.right
3	0.55235	C5, interspinalis.3, interspinalis.4, intertransversarius.dorsalis.3.left, intertransversarius.dorsalis.4.left, intertransversarius.ventralis.3.left, intertransversarius.ventralis.4.left, intertransversarius.dorsalis.3.right, intertransversarius.dorsalis.4.right, intertransversarius.ventralis.3.right, intertransversarius.ventralis.4.right
4	0.36822	clavicle.right, scapula.right, atlantoscapularis.reifht, cleido.mastoideus.right, trapezius.right
5	0.00021	mandible, sternum, hyoid, thyroid, tongue, sterno.mastoideus.left, sterno.mastoideus.right, sterno.maxillaris.left, sterno.maxillaris.right, sternoglossus.left, sternoglossus.right, sternothyroideus.left, sternothyroideus.right
6	0.36822	clavicle.left, scapula.left, atlantoscapularis.left, cleido.mastoideus.left, trapezius.left
7	0.00435	C6, C7, ribs.left, ribs.right, iliocostalis.cervicis.left, iliocostalis.cervicis.right, longus.capitis.left, scalenus.longus.left, scalenus.longus.right, serratus.ventralis.cervicis.left, serratus.ventralis.cervicis.right, interspinalis.5, interspinalis.6, intertransversarius.dorsalis.5.left, intertransversarius.dorsalis.6.left, intertransversarius.ventralis.5.left, intertransversarius.ventralis.6.left, intertransversarius.dorsalis.5.right, intertransversarius.dorsalis.6.right, intertransversarius.ventralis.5.right, intertransversarius.ventralis.6.right

Table A18 Connectivity modules identified for *Dasypus novemcinctus*.

ID	p-value	Elements
1	0.00585	C6, C7, ribs.left, ribs.right, iliocostalis.cervicis.left, iliocostalis.cervicis.right, scalenus.brevis.left, scalenus.brevis.right, scalenus.longus.left, scalenus.longus.right, serratus.ventralis.cervicis.left, serratus.ventralis.cervicis.right, interspinalis.4, interspinalis.5, intertransversarius.dorsalis.4.left, intertransversarius.dorsalis.5.left, intertransversarius.ventralis.4.left, intertransversarius.ventralis.5.left, intertransversarius.dorsalis.4.right, intertransversarius.dorsalis.5.right, intertransversarius.ventralis.4.right, intertransversarius.ventralis.5.right
2	0.22839	clavicle.left, scapula.left, cleido.mastoideus.left, trapezius.left
3	0.75162	thoracic.spine, longissimus.capitis.left, longissimus.capitis.right, semispinalis.capitis.right, interspinalis.6, intertransversarius.dorsalis.6.left, intertransversarius.ventralis.6.left, intertransversarius.dorsalis.6.right, intertransversarius.ventralis.6.right
4	0.22839	clavicle.right, scapula.right, cleido.mastoideus.right, trapezius.right
5	0.00037	mandible, sternum, hyoid, thyroid, sternohyoideus.left, sternohyoideus.right, sterno.mastoideus.left, sterno.mastoideus.right, sterno.maxillaris.left, sterno.maxillaris.right, sternothyroideus.left, sternothyroideus.right
6	0.40099	C3, C4, C5, longissimus.cervicis.left, longissimus.cervicis.right, longus.capitis.left, longus.capitis.right, longus.colli.left, longus.colli.right, semispinalis.capitis.left, splenius.capitis.left, splenius.capitis.right, interspinalis.2, interspinalis.3, intertransversarius.dorsalis.2.left, intertransversarius.dorsalis.3.left, intertransversarius.ventralis.2.left, intertransversarius.ventralis.3.left, intertransversarius.dorsalis.2.right, intertransversarius.dorsalis.3.right, intertransversarius.ventralis.2.right, intertransversarius.ventralis.3.right
7	0.00045	cranium, C1, C2, obliquus.capitis.caudalis.left, obliquus.capitis.caudalis.right, obliquus.capitis.cranialis.left, obliquus.capitis.cranialis.right, rectus.capitis.dorsalis.major.left, rectus.capitis.dorsalis.major.right, rectus.capitis.dorsalis.minor.left, rectus.capitis.dorsalis.minor.right, rectus.capitis.lateralis.left, rectus.capitis.lateralis.right, rectus.capitis.ventralis.left, rectus.capitis.ventralis.right, rhomboideus.cervicis.left, rhomboideus.cervicis.right, interspinalis.1, intertransversarius.dorsalis.1.left, intertransversarius.ventralis.1.left, intertransversarius.dorsalis.1.right, intertransversarius.ventralis.1.right

Table A19 Connectivity modules identified for *Didelphis virginiana*.

ID	p-value	Elements
1	5e-05	C6, C7, thoracic.spine, ribs.left, ribs.right, cervicalis.ascendens.left, cervicalis.ascendens.right, complexus.left, iliocostalis.dorsi.left, iliocostalis.dorsi.right, multifidius.submultifidius.1.left, multifidius.submultifidius.1.right, scalenus.anticus.left, scalenus.anticus.right, scalenus.posticus.left, scalenus.posticus.right, semispinalis.cervicis.left, semispinalis.cervicis.right, splenius.right, interspinalis.1, interspinalis.2, multifidius.submultifidius.5.left, multifidius.submultifidius.6.left, intertransversarii.ventralis.4.left, intertransversarii.ventralis.5.left, intertransversarii.ventralis.6.left, multifidius.submultifidius.5.right, multifidius.submultifidius.6.right, intertransversarii.ventralis.4.right, intertransversarii.ventralis.5.right, intertransversarii.ventralis.6.right
2	0.00245	sternum, hyoid, thyroid, omohyoid.left, omohyoid.right, sternohyoideus.left, sternohyoideus.right, sternothyroid.left, sternothyroid.right, sternomastoideus.left, sternomastoideus.right
3	0.00032	C2, C3, C4, C5, intertransversarii.dorsalis.longi.left, intertransversarii.dorsalis.longi.right, levator.scapulae.right, longissimus.capitis.left, longissimus.capitis.right, longissimus.cervicis.left, longissimus.cervicis.right, longus.capitis.left, longus.colli.left, longus.colli.right, obliquus.capitis.caudalis.left, obliquus.capitis.caudalis.right, rectus.capitis.dorsalis.major.left, rectus.capitis.dorsalis.major.right, spinalis.cervicis.left, spinalis.cervicis.right, splenius.left, multifidius.submultifidius.1.left.1, multifidius.submultifidius.2.left, multifidius.submultifidius.3.left, multifidius.submultifidius.4.left, intertransversarii.ventralis.1.left, intertransversarii.ventralis.2.left, intertransversarii.ventralis.3.left, multifidius.submultifidius.1.right.1, multifidius.submultifidius.2.right, multifidius.submultifidius.3.right, multifidius.submultifidius.4.right, intertransversarii.ventralis.1.right, intertransversarii.ventralis.2.right, intertransversarii.ventralis.3.right
4	0.00392	cranium, C1, clavicle.right, scapula.right, atlantoacromialis.right, atlantoscapularis.right, biventer.cervicis.left, biventer.cervicis.right, cleidomastoideus.right, complexus.right, longus.capitis.right, obliquus.capitis.cranialis.left, obliquus.capitis.cranialis.right, rectus.capitis.dorsalis.minor.left, rectus.capitis.dorsalis.minor.right, rectus.capitis.dorsalis.superficialis.left, rectus.capitis.dorsalis.superficialis.right, rectus.capitis.lateralis.left, rectus.capitis.lateralis.right, rectus.capitis.ventralis.left, rectus.capitis.ventralis.right, rhomboideus.right, trapezius.right
5	0.68552	clavicle.left, scapula.left, atlantoacromialis.left, atlantoscapularis.left, cleidomastoideus.left, levator.scapulae.left, rhomboideus.left, trapezius.left

Table A20 Connectivity modules identified for *Dugong dugon*.

ID	p-value	Elements
1	0.02227	cranium, C1, scapula.left, humerus.left, brachiocephalicus.left, cephalohumeralis.left, obliquus.capitis.caudalis.left, obliquus.capitis.caudalis.right, obliquus.capitis.cranialis.left, obliquus.capitis.cranialis.right, rectus.capitis.dorsalis.major.left, rectus.capitis.dorsalis.major.right, rectus.capitis.dorsalis.minor.left, rectus.capitis.dorsalis.minor.right, rectus.capitis.intermedius.left, rectus.capitis.intermedius.right, rectus.capitis.lateralis.left, rectus.capitis.lateralis.right, semispinalis.capitis.left, semispinalis.capitis.right, spinalis.cervicis, spinalis.cervicis.1
2	0.0187	sternum, hyoid, thyroid, sternomastoideus.left, sternomastoideus.right, sternothyroideus.left, sternothyroideus.right
3	0.00838	C5, C6, longissimus.capitis.right, longus.capitis.left, longus.capitis.right, semispinalis.dorsi.left, interspinalis.3, interspinalis.4, multifidius.submultifidius.3.left, multifidius.submultifidius.4.left, intertransversarius.dorsalis.3.left, intertransversarius.dorsalis.4.left, intertransversarius.dorsalis.5.left, intertransversarius.ventralis.3.left, intertransversarius.ventralis.4.left, multifidius.submultifidius.3.right, multifidius.submultifidius.4.right, intertransversarius.dorsalis.3.right, intertransversarius.dorsalis.4.right, intertransversarius.dorsalis.5.right, intertransversarius.ventralis.3.right, intertransversarius.ventralis.4.right, intertransversarius.ventralis.5.right
4	0.45549	scapula.right, humerus.right, brachiocephalicus.right, cephalohumeralis.right, trapezius.right
5	0.00297	C2, C3, C4, longissimus.dorsi.left, longissimus.dorsi.right, rectus.capitis.ventralis.left, rectus.capitis.ventralis.right, scalenus.anterior.left, scalenus.anterior.right, semispinalis.dorsi.right, serratus.magnus.left, serratus.magnus.right, trapezius.left, interspinalis.1, interspinalis.2, multifidius.submultifidius.1.left, multifidius.submultifidius.2.left, intertransversarius.dorsalis.1.left, intertransversarius.dorsalis.2.left, intertransversarius.ventralis.1.left, intertransversarius.ventralis.2.left, multifidius.submultifidius.1.right, multifidius.submultifidius.2.right, intertransversarius.dorsalis.1.right, intertransversarius.dorsalis.2.right, intertransversarius.ventralis.1.right, intertransversarius.ventralis.2.right
6	0.00531	C7, thoracic.spine, ribs.left, ribs.right, iliocostalis.cervicis.left, iliocostalis.cervicis.right, longissimus.capitis.left, longus.colli.left, longus.colli.right, rhomboideus.cervicis.left, rhomboideus.cervicis.right, interspinalis.5, interspinalis.6, multifidius.submultifidius.5.left, multifidius.submultifidius.6.left, intertransversarius.dorsalis.6.left, intertransversarius.ventralis.5.left, intertransversarius.ventralis.6.left, multifidius.submultifidius.5.right, multifidius.submultifidius.6.right, intertransversarius.dorsalis.6.right, intertransversarius.ventralis.6.right

Table A21 Connectivity modules identified for *Elephas maximus*.

ID	p-value	Elements
1	0.5	C2, obliquus.capitis.caudalis.left, obliquus.capitis.caudalis.right, rectus.capitis.dorsalis.major.left, rectus.capitis.dorsalis.major.right, rectus.capitis.externus.left, rectus.capitis.externus.right, interspinalis.1, multifidius.submultifidius.1.left, intertransversarius.dorsalis.1.left, intertransversarius.ventralis.1.left, multifidius.submultifidius.1.right, intertransversarius.dorsalis.1.right, intertransversarius.ventralis.1.right
2	0.00285	mandible, sternum, hyoid, thyroid, ribs.left, ribs.right, sternomandibularis.left, sternomandibularis.right, sternomastoideus.left, sternomastoideus.right, sternothyroideus.left, sternothyroideus.right
3	0.00035	C3, C4, C5, levator.scapulae.left, levator.scapulae.right, longus.capitis.left, longus.capitis.right, longus.colli.left, longus.colli.right, rectus.capitis.ventralis.left, rectus.capitis.ventralis.right, scalenus.anterior.left, scalenus.anterior.right, interspinalis.2, interspinalis.3, multifidius.submultifidius.2.left, multifidius.submultifidius.4.left, intertransversarius.dorsalis.2.left, intertransversarius.dorsalis.3.left, intertransversarius.ventralis.2.left, intertransversarius.ventralis.3.left, intertransversarius.ventralis.4.left, multifidius.submultifidius.2.right, intertransversarius.dorsalis.2.right, intertransversarius.dorsalis.3.right, intertransversarius.dorsalis.4.right, intertransversarius.ventralis.2.right, intertransversarius.ventralis.3.right
4	0	cranium, C1, scapula.left, scapula.right, humerus.left, humerus.right, nuchal.ligament, levator.anguli.scapulae.left, levator.anguliscapulae.right, mastohumeralis.left, mastohumeralis.right, obliquus.capitis.cranialis.left, obliquus.capitis.cranialis.right, occipitoscapularis.left, occipitoscapularis.right, rectus.capitis.dorsalis.minor.left, rectus.capitis.dorsalis.minor.right, rectus.capitis.lateralis.left, rectus.capitis.lateralis.right, rhomboideus.cervicis.left, rhomboideus.cervicis.right, splenius.left, splenius.right, trapezius.left, trapezius.right
5	0.00167	C6, C7, thoracic.spine, longissimus.capitis.left, longissimus.capitis.right, longissimus.cervicis.left, longissimus.cervicis.right, semispinalis.capitis.left, semispinalis.capitis.right, semispinalis.cervicis.left, semispinalis.cervicis.right, interspinalis.4, interspinalis.5, interspinalis.6, multifidius.submultifidius.3.left, multifidius.submultifidius.5.left, multifidius.submultifidius.6.left, intertransversarius.dorsalis.4.left, intertransversarius.dorsalis.5.left, intertransversarius.dorsalis.6.left, intertransversarius.ventralis.5.left, intertransversarius.ventralis.6.left, multifidius.submultifidius.3.right, multifidius.submultifidius.4.right, multifidius.submultifidius.5.right, multifidius.submultifidius.6.right, intertransversarius.dorsalis.5.right, intertransversarius.dorsalis.6.right, intertransversarius.ventralis.4.right, intertransversarius.ventralis.5.right, intertransversarius.ventralis.6.right

Table A22 Connectivity modules identified for *Equus caballus*.

ID	p-value	Elements
1	0.00055	mandible, sternum, hyoid, thyroid, omohyoideus.left, sternohyoideus.left, sternohyoideus.right, sternomandibularis.left, sternomandibularis.right, sternothyroideus.left, sternothyroideus.right
2	0	C5, C6, C7, ribs.left, ribs.right, iliocostalis.cervicis.left, iliocostalis.cervicis.right, longissimus.cervicis.left, longissimus.cervicis.right, scalenus.medius.left, scalenus.medius.right, scalenus.ventralis.left, scalenus.ventralis.right, serratus.ventralis.cervicis.left, spinalis.cervicis.left, spinalis.cervicis.right, interspinalis.4, interspinalis.5, multifidius.submultifidius.4.left, multifidius.submultifidius.5.left, multifidius.submultifidius.6.left, intertransversarius.dorsalis.4.left, intertransversarius.dorsalis.5.left, intertransversarius.ventralis.4.left, intertransversarius.ventralis.5.left, multifidius.submultifidius.4.right, multifidius.submultifidius.5.right, multifidius.submultifidius.6.right, intertransversarius.dorsalis.4.right, intertransversarius.dorsalis.5.right, intertransversarius.ventralis.4.right, intertransversarius.ventralis.5.right, intertransversarius.intermedius.5.left, intertransversarius.intermedius.5.right
3	0.42899	scapula.right, humerus.right, cleidomastoideus.right, omohyoideus.right, omotransversarius.right, serratus.ventralis.cervicis.right
4	0.00023	cranium, C1, scapula.left, humerus.left, cleidomastoideus.left, obliquus.capitis.caudalis.left, obliquus.capitis.caudalis.right, obliquus.capitis.cranialis.left, obliquus.capitis.cranialis.right, omotransversarius.left, rectus.capitis.dorsalis.major.left, rectus.capitis.dorsalis.major.right, rectus.capitis.dorsalis.minor.left, rectus.capitis.dorsalis.minor.right, rectus.capitis.lateralis.left, rectus.capitis.lateralis.right, rectus.capitis.ventralis.left, rectus.capitis.ventralis.right, semispinalis.capitis.b.left, semispinalis.capitis.b.right
5	0.04537	thoracic.spine, nuchal.ligament, longissimus.atlantis.left, longissimus.atlantis.right, longissimus.capitis.left, longissimus.capitis.right, rhomboideus.cervicis.left, rhomboideus.cervicis.right, splenius.capitis.left, splenius.capitis.right, trapezius.left, trapezius.right, interspinalis.6, intertransversarius.dorsalis.6.left, intertransversarius.ventralis.6.left, intertransversarius.dorsalis.6.right, intertransversarius.ventralis.6.right
6	4e-05	C2, C3, C4, longus.capitis.left, longus.capitis.right, longus.colli.left, longus.colli.right, splenius.cervicis.left, splenius.cervicis.right, interspinalis.1, interspinalis.2, interspinalis.3, multifidius.submultifidius.1.left, multifidius.submultifidius.2.left, multifidius.submultifidius.3.left, intertransversarius.dorsalis.1.left, intertransversarius.dorsalis.2.left, intertransversarius.dorsalis.3.left, intertransversarius.ventralis.1.left, intertransversarius.ventralis.2.left, intertransversarius.ventralis.3.left, multifidius.submultifidius.1.right, multifidius.submultifidius.2.right, multifidius.submultifidius.3.right, intertransversarius.dorsalis.1.right, intertransversarius.dorsalis.2.right, intertransversarius.dorsalis.3.right, intertransversarius.ventralis.1.right, intertransversarius.ventralis.2.right, intertransversarius.ventralis.3.right, intertransversarius.intermedius.2.left, intertransversarius.intermedius.3.left, intertransversarius.intermedius.4.left, intertransversarius.intermedius.2.right, intertransversarius.intermedius.3.right, intertransversarius.intermedius.4.right

Table A23 Connectivity modules identified for *Erinaceus europaeus*.

ID	p-value	Elements
1	0.00071	cranium, C1, clavicle.left, scapula.left, cleido.mastoideus.left, cleido.occipitalis.left, levator.scapulae.left, obliquus.capitis.caudalis.left, obliquus.capitis.caudalis.right, obliquus.capitis.cranialis.left, obliquus.capitis.cranialis.right, rectus.capitis.dorsalis.major.left, rectus.capitis.dorsalis.major.right, rectus.capitis.dorsalis.minor.left, rectus.capitis.dorsalis.minor.right, rectus.capitis.lateralis.left, rectus.capitis.lateralis.right, rectus.capitis.ventralis.left, rectus.capitis.ventralis.right, rhomboideus.cervicis.left
2	0.00139	clavicle.right, scapula.right, sternum, hyoid, thyroid, ribs.left, ribs.right, cleido.mastoideus.right, cleido.occipitalis.right, iliocostalis.cervicis.left, iliocostalis.cervicis.right, levator.scapulae.right, omohyoideus.left, omohyoideus.right, sternohyoideus.left, sternohyoideus.right, sterno.mastoideus.left, sterno.mastoideus.right, sternothyroideus.left, sternothyroideus.right
3	0.00077	C5, C6, C7, thoracic.spine, longissimus.capitis.left, longissimus.capitis.right, longus.atlantis.left, longus.atlantis.right, longus.capitis.left, longus.capitis.right, longus.colli.right, rhomboideus.cervicis.right, semispinalis.capitis.left, semispinalis.capitis.right, semispinalis.cervicis.left, semispinalis.cervicis.right, splenius.left, splenius.right, multifidius.submultifidius.4.left, multifidius.submultifidius.5.left, multifidius.submultifidius.6.left, intertransversarius.dorsalis.4.left, intertransversarius.dorsalis.5.left, intertransversarius.dorsalis.6.left, intertransversarius.ventralis.4.left, intertransversarius.ventralis.5.left, intertransversarius.ventralis.6.left, multifidius.submultifidius.4.right, multifidius.submultifidius.5.right, multifidius.submultifidius.6.right, intertransversarius.dorsalis.4.right, intertransversarius.dorsalis.5.right, intertransversarius.dorsalis.6.right, intertransversarius.ventralis.4.right, intertransversarius.ventralis.5.right, intertransversarius.ventralis.6.right
4	0.01809	C2, C3, C4, longus.colli.left, scalenus.left, scalenus.right, serratus.ventralis.cervicis.left, serratus.ventralis.cervicis.right, trapezius.anticus.left, trapezius.anticus.right, multifidius.submultifidius.1.left, multifidius.submultifidius.2.left, multifidius.submultifidius.3.left, intertransversarius.dorsalis.1.left, intertransversarius.dorsalis.2.left, intertransversarius.dorsalis.3.left, intertransversarius.ventralis.1.left, intertransversarius.ventralis.2.left, intertransversarius.ventralis.3.left, multifidius.submultifidius.1.right, multifidius.submultifidius.2.right, multifidius.submultifidius.3.right, intertransversarius.dorsalis.1.right, intertransversarius.dorsalis.2.right, intertransversarius.dorsalis.3.right, intertransversarius.ventralis.1.right, intertransversarius.ventralis.2.right, intertransversarius.ventralis.3.right

Table A24 Connectivity modules identified for *Felis silvestris*.

ID	p-value	Elements
1	0.05328	C2, C3, acromiotrapezius.left, longus.atlantis.left, longus.atlantis.right, longus.colli.right, rectus.capitis.dorsalis.intermedius.left, rectus.capitis.dorsalis.major.right, scalenus..right, interspinalis.1, interspinalis.2, intertransversarii.dorsalis.1.left, intertransversarii.dorsalis.2.left, intertransversarii.dorsalis.3.left, intertransversarii.dorsalis.2.right, intertransversarii.dorsalis.3.right, intertransversarius.intermedius.1.right, intertransversarius.intermedius.2.right, intertransversarius.intermedius.1.left, intertransversarius.intermedius.2.left
2	0	cranium, C1, scapula.left, scapula.right, humerus.left, humerus.right, cleidocervicalis.left, cleidocervicalis.right, cleidomastoideus.left, cleidomastoideus.right, levator.claviculae.left, levator.claviculae.right, obliquus.capitis.caudalis.left, obliquus.capitis.caudalis.right, obliquus.capitis.cranialis.left, obliquus.capitis.cranialis.right, rectus.capitis.dorsalis.intermedius.right, rectus.capitis.dorsalis.major.left, rectus.capitis.dorsalis.minor.left, rectus.capitis.dorsalis.minor.right, rectus.capitis.lateralis.left, rectus.capitis.lateralis.right, rectus.capitis.ventralis.left, rectus.capitis.ventralis.right, rhomboideus.capitis.left, rhomboideus.capitis.right, intertransversarii.dorsalis.1.right
3	0.14192	C4, C5, C6, complexus.left, complexus.right, longissimus.capitis.left, longissimus.capitis.right, longissimus.cervicis.left, longissimus.cervicis.right, longus.capitis.left, longus.capitis.right, serratus.ventralis.cervicis.left, serratus.ventralis.cervicis.right, spinalis.cervicis.left, spinalis.cervicis.right, interspinalis.3, interspinalis.4, intertransversarii.dorsalis.4.left, intertransversarii.dorsalis.5.left, intertransversarii.ventralis.left, intertransversarii.dorsalis.4.right, intertransversarii.dorsalis.5.right, intertransversarii.ventralis.right, intertransversarius.intermedius.3.right, intertransversarius.intermedius.4.right, intertransversarius.intermedius.3.left, intertransversarius.intermedius.4.left
4	0.00203	sternum, hyoid, thyroid, ribs.left, ribs.right, scalenus.left, sternohyoideus.left, sternohyoideus.right, sternomastoideus.left, sternomastoideus.right, sternothyroideus.left, sternothyroideus.right
5	0.69676	C7, thoracic.spine, acromiotrapezius.right, biventer.cervicis.left, biventer.cervicis.right, longus.colli.left, rhomboideus.cervicis.left, rhomboideus.cervicis.right, semispinalis.cervicis.left, semispinalis.cervicis.right, splenius.cervicis.left, splenius.cervicis.rigth, interspinalis.5, intertransversarii.dorsalis.6.left, intertransversarii.dorsalis.7.left, intertransversarii.dorsalis.6.right, intertransversarii.dorsalis.7.right

Table A25 Connectivity modules identified for *Galictis cuja*.

ID	p-value	Elements
1	0.00196	C2, C3, C4, interspinalis.1, interspinalis.2, intertransversarii.dorsalis.2.left, intertransversarii.dorsalis.2.right, intertransversarii.dorsalis.3.left, intertransversarii.dorsalis.3.right, intertransversarii.dorsalis.4.left, intertransversarii.ventralis.left, intertransversarii.ventralis.right, intertransversarius.intermedius.1.left, intertransversarius.intermedius.1.right, intertransversarius.intermedius.2.left, intertransversarius.intermedius.2.right, intertransversarius.intermedius.3.left, longissimus.cervicis.right, longus.capitis.left, longus.capitis.right, longus.colli.left, multifidius.submultifidius.1.left, multifidius.submultifidius.1.right, multifidius.submultifidius.2.left, multifidius.submultifidius.2.right, multifidius.submultifidius.3.left, multifidius.submultifidius.3.right, spinalis.cervicis.right
2	0.00472	C5, C6, C7, ribs.left, complexus.left, complexus.right, interspinalis.3, interspinalis.4, interspinalis.5, intertransversarii.dorsalis.4.right, intertransversarii.dorsalis.5.left, intertransversarii.dorsalis.5.right, intertransversarii.dorsalis.6.left, intertransversarii.dorsalis.6.right, intertransversarius.intermedius.3.right, intertransversarius.intermedius.4.left, intertransversarius.intermedius.4.right, longissimus.cervicis.left, longus.colli.right, multifidius.submultifidius.4.left, multifidius.submultifidius.4.right, multifidius.submultifidius.5.left, multifidius.submultifidius.5.right, scalenus.dorsalis.left, scalenus.dorsalis.right, scalenus.medius.left, scalenus.medius.right, scalenus.ventralis.left, spinalis.cervicis.left
3	0.00282	sternum, hyoid, thyroid, ribs.right, omohyoideus.left, omohyoideus.right, scalenus.ventralis.right, sternohyoideus.left, sternohyoideus.right, sternomastoideus.left, sternomastoideus.right, sternooccipitalis.left, sternooccipitalis.right, sternothyroideus.left, sternothyroideus.right
4	0	cranium, C1, scapula.left, scapula.right, humerus.left, humerus.right, cleidocervicalis.left, cleidocervicalis.right, cleidomastoideus.left, cleidomastoideus.right, intertransversarii.dorsalis.1.left, intertransversarii.dorsalis.1.right, levator.claviculae.left, levator.claviculae.right, obliquus.capitis.caudalis.left, obliquus.capitis.caudalis.right, obliquus.capitis.cranialis.left, obliquus.capitis.cranialis.right, omotrachelian.left, omotrachelian.right, rectus.capitis.dorsalis.intermedius.left, rectus.capitis.dorsalis.intermedius.right, rectus.capitis.dorsalis.major.left, rectus.capitis.dorsalis.major.right, rectus.capitis.dorsalis.minor.left, rectus.capitis.dorsalis.minor.right, rectus.capitis.lateralis.left, rectus.capitis.lateralis.right, rectus.capitis.ventralis.left, rectus.capitis.ventralis.right, rhomboideus.capitis.left, rhomboideus.capitis.right
5	0.02777	ligamentum.nuchae, thoracic.spine, biventer.cervicis.left, biventer.cervicis.right, intertransversarii.dorsalis.7.left, intertransversarii.dorsalis.7.right, longissimus.capitis.left, longissimus.capitis.right, rhomboideus.cervicis.left, rhomboideus.cervicis.right, splenius.left, splenius.right, trapezius.left, trapezius.right

Table A26 Connectivity modules identified for *Giraffa camelopardalis*.

ID	p-value	Elements
1	0.03765	C2, C3, C4, longissimus.cervicis.left, longissimus.cervicis.right, longus.capitis.left, longus.capitis.right, longus.colli.left, longus.colli.right, omohyoideus.left, scalenus.ventralis.left, scalenus.ventralis.right, semispinalis.cervicis.left, interspinalis.1, interspinalis.2, multifidius.submultifidius.1.left, multifidius.submultifidius.2.left, multifidius.submultifidius.3.left, intertransversarius.dorsalis.2.left, multifidius.submultifidius.1.right, multifidius.submultifidius.2.right, multifidius.submultifidius.3.right, intertransversarius.dorsalis.2.right
2	0.00054	cranium, C1, biventer.cervicis.left, biventer.cervicis.right, obliquus.capitis.caudalis.left, obliquus.capitis.caudalis.right, obliquus.capitis.cranialis.left, obliquus.capitis.cranialis.right, rectus.capitis.dorsalis.major.left, rectus.capitis.dorsalis.major.right, rectus.capitis.dorsalis.minor.left, rectus.capitis.dorsalis.minor.right, rectus.capitis.lateralis.left, rectus.capitis.lateralis.right, rectus.capitis.ventralis.left, rectus.capitis.ventralis.right, splenius.capitis.left, splenius.capitis.right
3	0.07988	C7, thoracic.spine, nuchal.ligament, complexus.left, complexus.right, iliocostalis.cervicis.left, iliocostalis.cervicis.right, rhomboideus.cervicis.left, rhomboideus.cervicis.right, semispinalis.cervicis.right, trapezius.left, trapezius.right, interspinalis.5, interspinalis.6, multifidius.submultifidius.5.left, multifidius.submultifidius.6.left, intertransversarius.dorsalis.5.left, intertransversarius.dorsalis.6.left, multifidius.submultifidius.5.right, multifidius.submultifidius.6.right, intertransversarius.dorsalis.5.right, intertransversarius.dorsalis.6.right
4	0.03006	C5, C6, scapula.left, scapula.right, humerus.left, humerus.right, cleidooccipitalis.left, cleidooccipitalis.right, longissimus.atlantis.left, longissimus.atlantis.right, longissimus.capitis.left, longissimus.capitis.right, omohyoideus.right, omotransversarius.left, omotransversarius.right, serratus.ventralis.cervicis.left, serratus.ventralis.cervicis.right, interspinalis.3, interspinalis.4, multifidius.submultifidius.4.left, intertransversarius.dorsalis.3.left, intertransversarius.dorsalis.4.left, intertransversarius.ventralis.longus.left, intertransversarius.ventralis.longus.right, multifidius.submultifidius.4.right, intertransversarius.dorsalis.3.right, intertransversarius.dorsalis.4.right
5	0.00023	mandible, sternum, hyoid, thyroid, ribs.left, ribs.right, scalenus.dorsalis.left, scalenus.dorsalis.right, scalenus.medius.left, scalenus.medius.right, scalenus.tertius.left, scalenus.tertius.right, sternomandibularis.left, sternomandibularis.right, sternothyrohyoideus.left, sternothyrohyoideus.right

Table A27 Connectivity modules identified for *Heteromys desmarestianus*.

ID	p-value	Elements
1	0.05536	C2, C3, C4, C5, intertransversarius.lateralis.longus.left, intertransversarius.lateralis.longus.right, longissimus.cervicis.left, longissimus.cervicis.right, longus.capitis.left, longus.capitis.right, rhomboideus.cervicis.left, rhomboideus.cervicis.right, scalenus.left, scalenus.right, semispinalis.cervicis.left, semispinalis.cervicis.right, serratus.ventralis.left, serratus.ventralis.right, multifidius.submultifidius.1.left, multifidius.submultifidius.2.left, intertransversarius.dorsalis.1.left, intertransversarius.dorsalis.2.left, intertransversarius.dorsalis.3.left, multifidius.submultifidius.1.right, multifidius.submultifidius.2.right, intertransversarius.dorsalis.1.right, intertransversarius.dorsalis.2.right, intertransversarius.dorsalis.3.right
2	0.37478	clavicle.right, scapula.right, acromiotrapezius.right, cleidomastoideus.right, cleidooccipitalis.right, omocervicalis.right, omohyoideus.right, rhomboideus.capitis.right
3	0.07763	C6, C7, thoracic.spine, iliocostalis.cervicis.left, iliocostalis.cervicis.right, longus.colli.left, longus.colli.right, semispinalis.capitis.left, semispinalis.capitis.right, splenius.left, splenius.right, multifidius.submultifidius.3.left, multifidius.submultifidius.4.left, multifidius.submultifidius.5.left, multifidius.submultifidius.6.left, intertransversarius.dorsalis.4.left, intertransversarius.dorsalis.5.left, intertransversarius.dorsalis.6.left, multifidius.submultifidius.3.right, multifidius.submultifidius.4.right, multifidius.submultifidius.5.right, multifidius.submultifidius.6.right, intertransversarius.dorsalis.4.right, intertransversarius.dorsalis.5.right, intertransversarius.dorsalis.6.right
4	0.08267	sternum, ribs.left, ribs.right, longissimus.capitis.left, longissimus.capitis.right, sternohyoideus.left, sternohyoideus.right, sternomastoideus.left, sternomastoideus.right, sternothyroideus.left, sternothyroideus.right
5	0.15519	clavicle.left, scapula.left, hyoid, thyroid, acromiotrapezius.left, cleidomastoideus.left, cleidooccipitalis.left, omocervicalis.left, omohyoideus.left, rhomboideus.capitis.left
6	0.00145	cranium, C1, obliquus.capitis.caudalis.left, obliquus.capitis.caudalis.right, obliquus.capitis.cranialis.left, obliquus.capitis.cranialis.right, rectus.capitis.dorsalis.major.left, rectus.capitis.dorsalis.major.right, rectus.capitis.dorsalis.minor.left, rectus.capitis.dorsalis.minor.right, rectus.capitis.lateralis.left, rectus.capitis.lateralis.right, rectus.capitis.ventralis.left, rectus.capitis.ventralis.right

Table A28 Connectivity modules identified for *Homo sapiens*.

ID	p-value	Elements
1	0.02531	C6, C7, thoracic.spine, longissimus.capitis.left, longissimus.capitis.right, longus.colli.left, longus.colli.right, rhomboideus.minor.left, rhomboideus.minor.right, semispinalis.capitis.left, semispinalis.capitis.right, semispinalis.cervicis.left, spinalis.cervicis.left, spinalis.cervicis.right, interspinalis.5, interspinalis.6, multifidius.submultifidius.6.left, intertransversarius.dorsalis.5.left, intertransversarius.dorsalis.6.left, intertransversarius.ventralis.5.left, intertransversarius.ventralis.6.left, multifidius.submultifidius.6.right, intertransversarius.dorsalis.5.right, intertransversarius.dorsalis.6.right, intertransversarius.ventralis.5.right, intertransversarius.ventralis.6.right
2	0.00423	C4, C5, ribs.left, ribs.right, iliocostalis.cervicis.left, iliocostalis.cervicis.right, longus.capitis.left, longus.capitis.right, scalenus.anticus.left, scalenus.anticus.right, scalenus.medius.left, scalenus.medius.right, scalenus.posticus.left, scalenus.posticus.right, interspinalis.3, interspinalis.4, multifidius.submultifidius.5.left, intertransversarius.dorsalis.3.left, intertransversarius.dorsalis.4.left, intertransversarius.ventralis.3.left, intertransversarius.ventralis.4.left, multifidius.submultifidius.5.right, intertransversarius.dorsalis.3.right, intertransversarius.dorsalis.4.right, intertransversarius.ventralis.3.right, intertransversarius.ventralis.4.right
3	0.03763	cranium, C1, nuchal.ligament, obliquus.capitis.caudalis.left, obliquus.capitis.caudalis.right, obliquus.capitis.cranialis.left, obliquus.capitis.cranialis.right, rectus.capitis.dorsalis.major.left, rectus.capitis.dorsalis.major.right, rectus.capitis.dorsalis.minor.left, rectus.capitis.dorsalis.minor.right, rectus.capitis.lateralis.left, rectus.capitis.lateralis.right, rectus.capitis.ventralis.left, rectus.capitis.ventralis.right, splenius.capitis.left, splenius.capitis.right, splenius.cervicis.left, splenius.cervicis.right, trapezius.left, trapezius.right
4	4e-05	clavicle.left, clavicle.right, scapula.left, scapula.right, sternum, hyoid, thyroid, omohyoideus.left, omohyoideus.right, sterno.cleido.mastoideus.left, sterno.cleido.mastoideus.right, sternohyoideus.left, sternohyoideus.right, sternothyroideus.left, sternothyroideus.right
5	0.12286	C2, C3, levator.scapulae.left, levator.scapulae.right, longissimus.cervicis.left, longissimus.cervicis.right, semispinalis.cervicis.right, interspinalis.1, interspinalis.2, multifidius.submultifidius.1.left, multifidius.submultifidius.2.left, multifidius.submultifidius.3.left, multifidius.submultifidius.4.left, intertransversarius.dorsalis.1.left, intertransversarius.dorsalis.2.left, intertransversarius.ventralis.1.left, intertransversarius.ventralis.2.left, multifidius.submultifidius.1.right, multifidius.submultifidius.2.right, multifidius.submultifidius.3.right, multifidius.submultifidius.4.right, intertransversarius.dorsalis.1.right, intertransversarius.dorsalis.2.right, intertransversarius.ventralis.1.right, intertransversarius.ventralis.2.right

Table A29 Connectivity modules identified for *Kogia breviceps*.

ID	p-value	Elements
1	0.03821	scapula.left, humerus.left, atlantoscapularis.ventralis.left, cleidomastoideus.left, rhomboideus.capitis.left
2	0.03821	scapula.right, humerus.right, atlantoscapularis.ventralis.right, cleidomastoideus.right, rhomboideus.capitis.right
3	0.00131	sternum, hyoid, thyroid, sternohyoideus.left, sternohyoideus.right, sternomastoideus.left, sternomastoideus.right, sternothyroideus.left, sternothyroideus.right
4	0.00264	C2, C3, C4, longus.colli.left, spinalis.cervicis.left, spinalis.cervicis.right, interspinalis.1, interspinalis.2, multifidius.submultifidius.1.left, multifidius.submultifidius.2.left, multifidius.submultifidius.3.left, intertransversarius.dorsalis.2.left, intertransversarius.ventralis.2.left, multifidius.submultifidius.1.right, multifidius.submultifidius.2.right, multifidius.submultifidius.3.right, intertransversarius.dorsalis.2.right, intertransversarius.ventralis.2.right
5	1e-05	cranium, C1, ribs.left, ribs.right, longissimus.capitis.left, longissimus.capitis.right, obliquus.capitis.cranialis.left, obliquus.capitis.cranialis.right, rectus.capitis.dorsalis.minor.left, rectus.capitis.dorsalis.minor.right, rectus.capitis.lateralis.left, rectus.capitis.lateralis.right, rectus.capitis.ventralis.left, rectus.capitis.ventralis.right, scalenus.dorsalis.left, scalenus.dorsalis.right, scalenus.medius.left, scalenus.medius.right, transversarius.capitis.left, transversarius.capitis.right
6	0.00235	C5, C6, longus.colli.right, interspinalis.3, interspinalis.4, interspinalis.5, multifidius.submultifidius.4.left, multifidius.submultifidius.5.left, intertransversarius.dorsalis.3.left, intertransversarius.dorsalis.4.left, intertransversarius.dorsalis.5.left, intertransversarius.ventralis.3.left, intertransversarius.ventralis.4.left, multifidius.submultifidius.4.right, multifidius.submultifidius.5.right, intertransversarius.dorsalis.3.right, intertransversarius.dorsalis.4.right, intertransversarius.ventralis.3.right, intertransversarius.ventralis.4.right
7	0.00086	C7, thoracic.spine, biventer.cervicis.left, biventer.cervicis.right, iliocostalis.capitis.left, iliocostalis.capitis.right, semispinalis.cervicis.left, semispinalis.cervicis.right, splenius.left, splenius.right, interspinalis.6, multifidius.submultifidius.6.left, intertransversarius.dorsalis.6.left, intertransversarius.ventralis.5.left, intertransversarius.ventralis.6.left, multifidius.submultifidius.6.right, intertransversarius.dorsalis.5.right, intertransversarius.dorsalis.6.right, intertransversarius.ventralis.5.right, intertransversarius.ventralis.6.right

Table A30 Connectivity modules identified for *Loris tardigradus*.

ID	p-value	Elements
1	0.06377	C2, C3, C4, longissimus.cervicis.left, longus.capitis.left, longus.capitis.right, longus.colli.left, scalenus.medius.left, scalenus.medius.right, semispinalis.cervicis.right, serratus.ventralis.cervicis.left, serratus.ventralis.cervicis.left.1, splenius.capitis.left, splenius.capitis.right, interspinalis.1, interspinalis.2, multifidius.submultifidius.1.left, multifidius.submultifidius.3.left, multifidius.submultifidius.4.left, intertransversarius.dorsalis.1.left, intertransversarius.ventralis.1.left, intertransversarius.ventralis.2.left, multifidius.submultifidius.1.right, multifidius.submultifidius.3.right, multifidius.submultifidius.4.right, intertransversarius.dorsalis.1.right, intertransversarius.ventralis.1.right, intertransversarius.ventralis.2.right
2	0.00077	cranium, C1, clavicle.left, scapula.left, atlantoscapularis.anterior.left, cleidomastoideus.left, obliquus.capitis.caudalis.left, obliquus.capitis.caudalis.right, obliquus.capitis.cranialis.left, obliquus.capitis.cranialis.right, rectus.capitis.dorsalis.major.left, rectus.capitis.dorsalis.major.right, rectus.capitis.dorsalis.minor.left, rectus.capitis.dorsalis.minor.right, rectus.capitis.lateralis.left, rectus.capitis.lateralis.right, rectus.capitis.ventralis.left, rectus.capitis.ventralis.right, rhomboideus.capitis.left, trapezius.left
3	0.85385	C5, interspinalis.3, interspinalis.4, multifidius.submultifidius.2.left, multifidius.submultifidius.5.left, intertransversarius.dorsalis.2.left, intertransversarius.dorsalis.4.left, intertransversarius.ventralis.3.left, intertransversarius.ventralis.4.left, multifidius.submultifidius.2.right, multifidius.submultifidius.5.right, intertransversarius.dorsalis.2.right, intertransversarius.dorsalis.4.right, intertransversarius.ventralis.3.right, intertransversarius.ventralis.4.right
4	0.02154	C6, C7, thoracic.spine, complexus.left, complexus.right, longissimus.capitis.left, longissimus.capitis.right, longissimus.cervicis.right, longus.colli.right, rhomboideus.minor.left, rhomboideus.minor.right, semispinalis.cervicis.left, spinalis.cervicis.left, spinalis.cervicis.right, interspinalis.5, interspinalis.6, multifidius.submultifidius.6.left, intertransversarius.dorsalis.3.left, intertransversarius.dorsalis.5.left, intertransversarius.dorsalis.6.left, intertransversarius.ventralis.5.left, intertransversarius.ventralis.6.left, multifidius.submultifidius.6.right, intertransversarius.dorsalis.3.right, intertransversarius.dorsalis.5.right, intertransversarius.dorsalis.6.right, intertransversarius.ventralis.5.right, intertransversarius.ventralis.6.right
5	0.35463	clavicle.right, scapula.right, atlantoscapularis.anterior.right, cleidomastoideus.right, omohyoideus.right, rhomboideus.capitis.right, trapezius.right
6	0.01359	sternum, hyoid, thyroid, ribs.left, ribs.right, iliocostalis.dorsalis.left, iliocostalis.dorsalis.right, omohyoideus.left, scalenus.posticus.left, scalenus.posticus.right, sternohyoideus.left, sternohyoideus.right, sternomastoideus.left, sternomastoideus.right, sternothyroideus.left, sternothyroideus.right

Table A31 Connectivity modules identified for *Macaca mulatta*.

ID	p-value	Elements
1	0.00022	cranium, C1, clavicle.left, scapula.left, atlantoscapularis.anterior.left, atlantoscapularis.posterior.left, biventer.cervicis.left, biventer.cervicis.right, cleidomastoideus.left, cleidooccipitalis.left, obliquus.capitis.cranialis.left, obliquus.capitis.cranialis.right, rectus.capitis.dorsalis.major.right, rectus.capitis.dorsalis.minor.left, rectus.capitis.dorsalis.minor.right, rectus.capitis.lateralis.left, rectus.capitis.lateralis.right, rectus.capitis.ventralis.left, rectus.capitis.ventralis.right, rhomboideus.capitis.left, trapezius.left
2	0.00188	clavicle.right, scapula.right, sternum, hyoid, thyroid, atlantoscapularis.anterior.right, atlantoscapularis.posterior.right, cleidomastoideus.right, cleidooccipitalis.right, omohyoideus.left, omohyoideus.right, rhomboideus.capitis.right, sternohyoideus.left, sternohyoideus.right, sternomastoideus.left, sternomastoideus.right, sternothyroideus.left, sternothyroideus.right, trapezius.right
3	0.85385	C2, obliquus.capitis.caudalis.left, obliquus.capitis.caudalis.right, rectus.capitis.dorsalis.major.left, interspinalis.1, multifidius.submultifidius.1.left, multifidius.submultifidius.2.left, multifidius.submultifidius.3.left, intertransversarius.dorsalis.1.left, intertransversarius.ventralis.1.left, multifidius.submultifidius.1.right, multifidius.submultifidius.2.right, multifidius.submultifidius.3.right, intertransversarius.dorsalis.1.right, intertransversarius.ventralis.1.right
4	0.13821	C6, C7, thoracic.spine, longissimus.capitis.left, longissimus.capitis.right, longissimus.cervicis.right, rhomboideus.minor.left, rhomboideus.minor.right, semispinalis.cervicis.left, semispinalis.cervicis.right, spinalis.cervicis.left, spinalis.cervicis.right, splenius.capitis.left, splenius.capitis.right, interspinalis.4, interspinalis.5, interspinalis.6, multifidius.submultifidius.6.left, intertransversarius.dorsalis.4.left, intertransversarius.dorsalis.5.left, intertransversarius.dorsalis.6.left, intertransversarius.ventralis.4.left, intertransversarius.ventralis.5.left, intertransversarius.ventralis.6.left, multifidius.submultifidius.6.right, intertransversarius.dorsalis.3.right, intertransversarius.dorsalis.5.right, intertransversarius.dorsalis.6.right, intertransversarius.ventralis.5.right, intertransversarius.ventralis.6.right
5	0.01653	C3, C4, C5, complexus.left, complexus.right, longissimus.cervicis.left, longus.capitis.left, longus.capitis.right, longus.colli.left, longus.colli.right, scalenus.anticus.left, scalenus.anticus.right, serratus.ventralis.cervicis.left, serratus.ventralis.cervicis.left.1, interspinalis.2, interspinalis.3, multifidius.submultifidius.4.left, multifidius.submultifidius.5.left, intertransversarius.dorsalis.2.left, intertransversarius.dorsalis.3.left, intertransversarius.ventralis.2.left, intertransversarius.ventralis.3.left, multifidius.submultifidius.4.right, multifidius.submultifidius.5.right, intertransversarius.dorsalis.2.right, intertransversarius.dorsalis.4.right, intertransversarius.ventralis.2.right, intertransversarius.ventralis.3.right, intertransversarius.ventralis.4.right
6	0.84134	ribs.left, iliocostalis.dorsalis.left, scalenus.medius.left, scalenus.posticus.left
7	0.84134	ribs.right, iliocostalis.dorsalis.right, scalenus.medius.right, scalenus.posticus.right

Table A32 Connectivity modules identified for *Macropus rufus*.

ID	p-value	Elements
1	0.00655	C2, C3, C4, acromiotrachelien.left, longissimus.atlantis.left, longissimus.atlantis.right, longissimus.capitis.left, obliquus.capitis.caudalis.left, obliquus.capitis.caudalis.right, semispinalis.capitis.right, splenius.cervicis.left, splenius.cervicis.right, multifidius.submultifidius.1.left, multifidius.submultifidius.2.left, multifidius.submultifidius.3.left, intertransversarii.dorsalis.1.left, intertransversarii.dorsalis.2.left, intertransversarii.dorsalis.3.left, intertransversarii.ventralis.1.left, intertransversarii.ventralis.2.left, intertransversarii.ventralis.3.left, multifidius.submultifidius.1.right, multifidius.submultifidius.2.right, multifidius.submultifidius.3.right, intertransversarii.dorsalis.1.right, intertransversarii.dorsalis.2.right, intertransversarii.dorsalis.3.right, intertransversarii.ventralis.1.right, intertransversarii.ventralis.2.right, intertransversarii.ventralis.3.right
2	0.00039	cranium, C1, clavicle.right, scapula.right, nuchal.ligament, acromiotrachelien.right, atlantoscapularis.right, cleidomastoideus.right, obliquus.capitis.cranialis.left, obliquus.capitis.cranialis.right, rectus.capitis.dorsalis.major.left, rectus.capitis.dorsalis.major.right, rectus.capitis.dorsalis.minor.left, rectus.capitis.dorsalis.minor.right, rectus.capitis.dorsalis.superficialis.left, rectus.capitis.dorsalis.superficialis.right, rectus.capitis.lateralis.left, rectus.capitis.lateralis.right, rhomboideus.left, rhomboideus.right, semispinalis.capitis.left, splenius.capitis.left, splenius.capitis.right, trapezius.right
3	0	C5, C6, C7, thoracic.spine, ribs.left, ribs.right, iliocostalis.dorsi.left, iliocostalis.dorsi.right, longissimus.capitis.right, longissimus.cervicis.left, longissimus.cervicis.right, longus.capitis.left, longus.capitis.right, longus.colli.left, longus.colli.right, scalenus.medius.left, scalenus.medius.right, scalenus.posticus.left, scalenus.posticus.right, serratus.magnus.right, interspinalis.6, multifidius.submultifidius.4.left, multifidius.submultifidius.5.left, multifidius.submultifidius.6.left, intertransversarii.dorsalis.4.left, intertransversarii.dorsalis.5.left, intertransversarii.dorsalis.6.left, intertransversarii.ventralis.4.left, intertransversarii.ventralis.5.left, intertransversarii.ventralis.6.left, spinalis.cervicis.left, multifidius.submultifidius.4.right, multifidius.submultifidius.5.right, multifidius.submultifidius.6.right, intertransversarii.dorsalis.4.right, intertransversarii.dorsalis.5.right, intertransversarii.dorsalis.6.right, intertransversarii.ventralis.4.right, intertransversarii.ventralis.5.right, intertransversarii.ventralis.6.right, spinalis.cervicis.right
4	0.00151	clavicle.left, scapula.left, sternum, hyoid, thyroid, atlantoscapularis.left, cleidomastoideus.left, omohyoideus.left, omohyoideus.right, serratus.magnus.left, sternohyoideus.left, sternohyoideus.right, sternomastoideus.left, sternomastoideus.right, sternothyroideus.left, sternothyroideus.right, trapezius.left

Table A33 Connectivity modules identified for *Macrotis lagotis*.

ID	p-value	Elements
1	0.92103	C5, scalenus.posticus.left, interspinalis.3, interspinalis.4, multifidius.submultifidius.4.left, intertransversarii.dorsalis.3.left, intertransversarii.dorsalis.4.left, intertransversarii.ventralis.3.left, intertransversarii.ventralis.4.left, multifidius.submultifidius.4.right, intertransversarii.dorsalis.3.right, intertransversarii.dorsalis.4.right, intertransversarii.ventralis.3.right, intertransversarii.ventralis.4.right
2	0.95573	ribs.right, longus.colli.right, scalenus.medius.right, scalenus.posticus.right
3	0.00123	sternum, hyoid, thyroid, omohyoideus.left, omohyoideus.right, sternohyoideus.left, sternohyoideus.right, sternomastoideus.left, sternomastoideus.right, sternothyroideus.left, sternothyroideus.right
4	0.01198	C6, C7, thoracic.spine, ribs.left, complexus.right, iliocostalis.dorsi.left, iliocostalis.dorsi.right, longissimus.capitis.left, longus.colli.left, scalenus.medius.left, semispinalis.cervicis.left, semispinalis.cervicis.right, splenius.capitis.left, splenius.capitis.right, splenius.cervicis.right, interspinalis.5, interspinalis.6, multifidius.submultifidius.5.left, multifidius.submultifidius.6.left, intertransversarii.dorsalis.5.left, intertransversarii.dorsalis.6.left, intertransversarii.ventralis.5.left, intertransversarii.ventralis.6.left, spinalis.cervicis.left, multifidius.submultifidius.5.right, multifidius.submultifidius.6.right, intertransversarii.dorsalis.5.right, intertransversarii.dorsalis.6.right, intertransversarii.ventralis.5.right, intertransversarii.ventralis.6.right
5	0.09181	C2, C3, C4, complexus.left, longissimus.capitis.right, longissimus.cervicis.left, longissimus.cervicis.right, rhomboideus.cervicis.left, serratus.magnus.left, serratus.magnus.right, splenius.cervicis.left, interspinalis.1, interspinalis.2, multifidius.submultifidius.1.left, multifidius.submultifidius.2.left, multifidius.submultifidius.3.left, intertransversarii.dorsalis.1.left, intertransversarii.dorsalis.2.left, intertransversarii.ventralis.1.left, intertransversarii.ventralis.2.left, multifidius.submultifidius.1.right, multifidius.submultifidius.2.right, multifidius.submultifidius.3.right, intertransversarii.dorsalis.1.right, intertransversarii.dorsalis.2.right, intertransversarii.ventralis.1.right, intertransversarii.ventralis.2.right, spinalis.cervicis.right
6	0.5	scapula.right, humerus.right, atlantoacromialis.right, atlantoscapularis.right, cleidooccipitalis.right, rhomboideus.capitis.right, rhomboideus.cervicis.right, trapezius.right
7	0.002	cranium, C1, scapula.left, humerus.left, atlantoacromialis.left, atlantoscapularis.left, biventer.cervicis.left, biventer.cervicis.right, cleidooccipitalis.left, longus.capitis.left, longus.capitis.right, obliquus.capitis.caudalis.left, obliquus.capitis.caudalis.right, obliquus.capitis.cranialis.left, obliquus.capitis.cranialis.right, rectus.capitis.dorsalis.major.left, rectus.capitis.dorsalis.major.right, rectus.capitis.dorsalis.minor.left, rectus.capitis.dorsalis.minor.right, rectus.capitis.dorsalis.superficialis.left, rectus.capitis.dorsalis.superficialis.right, rectus.capitis.lateralis.left, rectus.capitis.lateralis.right, rhomboideus.capitis.left, trapezius.left

Table A34 Connectivity modules identified for *Manis pentadactyla*.

ID	p-value	Elements
1	0.20531	C2, C3, longus.capitis.right, rectus.capitis.dorsalis.major.right, scalenus.longus.left, scalenus.longus.right, serratus.ventralis.cervicis.left, serratus.ventralis.cervicis.right, splenius.capitis.right, interspinalis.1, interspinalis.2, intertransversarius.dorsalis.1.left, intertransversarius.dorsalis.2.left, intertransversarius.ventralis.1.left, intertransversarius.dorsalis.1.right, intertransversarius.dorsalis.2.right, intertransversarius.ventralis.1.right
2	0.20356	C6, C7, thoracic.spine, iliocostalis.cervicis.left, iliocostalis.cervicis.right, longissimus.cervicis.left, longissimus.cervicis.right, longus.colli.left, longus.colli.right, semispinalis.capitis.left, semispinalis.capitis.right, semispinalis.cervicis.left, semispinalis.cervicis.right, trapezius.left, interspinalis.5, interspinalis.6, intertransversarius.dorsalis.5.left, intertransversarius.dorsalis.6.left, intertransversarius.ventralis.5.left, intertransversarius.ventralis.6.left, intertransversarius.dorsalis.5.right, intertransversarius.dorsalis.6.right, intertransversarius.ventralis.5.right, intertransversarius.ventralis.6.right
3	0.02714	C4, C5, longissimus.capitis.left, longissimus.capitis.right, longus.capitis.left, scalenus.brevis.left, scalenus.brevis.right, splenius.capitis.left, interspinalis.3, interspinalis.4, intertransversarius.dorsalis.3.left, intertransversarius.dorsalis.4.left, intertransversarius.ventralis.2.left, intertransversarius.ventralis.3.left, intertransversarius.ventralis.4.left, intertransversarius.dorsalis.3.right, intertransversarius.dorsalis.4.right, intertransversarius.ventralis.2.right, intertransversarius.ventralis.3.right, intertransversarius.ventralis.4.right
4	0.00082	sternum, hyoid, thyroid, ribs.left, ribs.right, tongue, sternoglossus.left, sternoglossus.right, sternohyoideus.left, sternohyoideus.right, sterno.mastoideus.left, sterno.mastoideus.right, sternothyroideus.left, sternothyroideus.right
5	6e-05	cranium, C1, humerus.left, humerus.right, scapula.left, scapula.right, cleidohumeralis.left, cleidohumeralis.right, levator.claviculae.left, levator.claviculae.right, mastoscapularis.left, mastoscapularis.right, obliquus.capitis.caudalis.left, obliquus.capitis.caudalis.right, obliquus.capitis.cranialis.left, obliquus.capitis.cranialis.right, rectus.capitis.dorsalis.major.left, rectus.capitis.dorsalis.minor.left, rectus.capitis.dorsalis.minor.right, rectus.capitis.lateralis.left, rectus.capitis.lateralis.right, rhomboideus.capitis.left, rhomboideus.capiti.right, rhomboideus.cervicis.left, rhomboideus.cervicis.right, trapezius.right

Table A35 Connectivity modules identified for *Micropotamogale ruwenzorii*.

ID	p-value	Elements
1	4e-05	scapula.left, scapula.right, sternum, hyoid, thyroid, clavicula.left, clavicula.right, omocervicalis.left, omocervicalis.right, omohyoid.left, omohyoid.right, sternohyoid.left, sternohyoid.right, sternomastoideus.left, sternomastoideus.right, sternooccipitalis.left, sternothyroid.left, sternothyroid.right
2	0.00988	cranium, C1, biventer.cervicis.left, biventer.cervicis.left.1, longus.capitis.left, longus.capitis.right, obliquus.capitis.caudalis.left, obliquus.capitis.caudalis.right, obliquus.capitis.cranialis.left, obliquus.capitis.cranialis.right, rectus.capitis.dorsalis.major.left, rectus.capitis.dorsalis.major.right, rectus.capitis.dorsalis.minor.left, rectus.capitis.dorsalis.minor.right, rectus.capitis.lateralis.left, rectus.capitis.lateralis.right, rectus.capitis.lateralis.right.1, rectus.capitis.ventralis.left, rhomboideus.left, rhomboideus.right, sternooccipitalis.right, trapezius.left, trapezius.right
3	0.74307	C5, interspinalis.3, interspinalis.4, intertransversarius.dorsalis.3.left, intertransversarius.dorsalis.4.left, intertransversarius.ventralis.3.left, intertransversarius.ventralis.4.left, multifidius.submultifidius.4.right, intertransversarius.dorsalis.3.right, intertransversarius.dorsalis.4.right, intertransversarius.ventralis.3.right, intertransversarius.ventralis.4.right
4	0.00136	C2, C3, C4, ribs.left, ribs.right, iliocostalis.cervicis.left, iliocostalis.cervicis.right, scalenus.medius.left, scalenus.medius.right, scalenus.supracostalis.left, scalenus.supracostalis.right, serratus.ventralis.cervicis.left, serratus.ventralis.cervicis.right, interspinalis.1, interspinalis.2, multifidius.submultifidius.1.left, multifidius.submultifidius.2.left, multifidius.submultifidius.3.left, intertransversarius.dorsalis.1.left, intertransversarius.dorsalis.2.left, intertransversarius.ventralis.1.left, intertransversarius.ventralis.2.left, multifidius.submultifidius.1.right, multifidius.submultifidius.2.right, multifidius.submultifidius.3.right, intertransversarius.dorsalis.1.right, intertransversarius.dorsalis.2.right, intertransversarius.ventralis.1.right, intertransversarius.ventralis.2.right
5	0.03031	C6, C7, thoracic.spine, biventer.cervicis.right, biventer.cervicis.right.1, longissimus.capitis.left, longissimus.capitis.right, longissimus.cervicis.left, longissimus.cervicis.right, splenius.left, splenius.right, interspinalis.5, interspinalis.6, multifidius.submultifidius.4.left, multifidius.submultifidius.5.left, multifidius.submultifidius.6.left, intertransversarius.dorsalis.5.left, intertransversarius.dorsalis.6.left, intertransversarius.ventralis.5.left, intertransversarius.ventralis.6.left, multifidius.submultifidius.5.right, multifidius.submultifidius.6.right, intertransversarius.dorsalis.5.right, intertransversarius.dorsalis.6.right, intertransversarius.ventralis.5.right, intertransversarius.ventralis.6.right

Table A36 Connectivity modules identified for *Neotoma fuscipes*.

ID	p-value	Elements
1	7e-05	C5, C6, C7, thoracic.spine, acromiotrapezius.left, acromiotrapezius.right, semispinalis.capitis.left, semispinalis.capitis.right, serratus.ventralis.right, longissimus.capitis.left, longissimus.capitis.right, longissimus.cervicis.left, longissimus.cervicis.right, longus.colli.left, longus.colli.right, rhomboideus.anticus.right, splenius.left, splenius.right, semispinalis.cervicis.left, semispinalis.cervicis.right, interspinalis.4, interspinalis.5, interspinalis.6, multifidius.submultifidius.4.left, multifidius.submultifidius.5.left, multifidius.submultifidius.6.left, intertransversarius.dorsalis.4.left, intertransversarius.dorsalis.5.left, intertransversarius.dorsalis.6.left, intertransversarius.ventralis.4.left, intertransversarius.ventralis.5.left, intertransversarius.ventralis.6.left, multifidius.submultifidius.4.right, multifidius.submultifidius.5.right, multifidius.submultifidius.6.right, intertransversarius.dorsalis.4.right, intertransversarius.dorsalis.5.right, intertransversarius.dorsalis.6.right, intertransversarius.ventralis.4.right, intertransversarius.ventralis.5.right, intertransversarius.ventralis.6.right
2	1e-05	cranium, C1, clavicle.left, clavicle.right, scapula.right, cleidomastoideus.left, cleidomastoideus.right, cleidotrapezius.left, cleidotrapezius.right, levator.claviculae.left, levator.claviculae.right, obliquus.capitis.caudalis.left, obliquus.capitis.caudalis.right, obliquus.capitis.cranialis.left, obliquus.capitis.cranialis.right, rectus.capitis.dorsalis.major.left, rectus.capitis.dorsalis.major.right, rectus.capitis.dorsalis.minor.left, rectus.capitis.dorsalis.minor.right, rectus.capitis.dorsalis.superficialis.left, rectus.capitis.dorsalis.superficialis.right, rectus.capitis.lateralis.left, rectus.capitis.lateralis.right, rectus.capitis.ventralis.left, rectus.capitis.ventralis.right, rhomboideus.capitis.right
3	0.04027	sternum, hyoid, thyroid, ribs.left, ribs.right, iliocostalis.cervicis.left, iliocostalis.cervicis.right, omohyoideus.right, sternohyoideus.left, sternohyoideus.right, sternomastoideus.left, sternomastoideus.right, sternothyroideus.left, sternothyroideus.right
4	0.00199	C2, C3, C4, longus.atlantis.left, longus.atlantis.right, longus.capitis.left, longus.capitis.right, scalenus.anticus.left, scalenus.anticus.right, scalenus.medius.left, scalenus.medius.right, scalenus.tertius.left, scalenus.tertius.right, interspinalis.1, interspinalis.2, interspinalis.3, multifidius.submultifidius.1.left, multifidius.submultifidius.2.left, multifidius.submultifidius.3.left, intertransversarius.dorsalis.1.left, intertransversarius.dorsalis.2.left, intertransversarius.dorsalis.3.left, intertransversarius.ventralis.1.left, intertransversarius.ventralis.2.left, intertransversarius.ventralis.3.left, multifidius.submultifidius.1.right, multifidius.submultifidius.2.right, multifidius.submultifidius.3.right, intertransversarius.dorsalis.1.right, intertransversarius.dorsalis.2.right, intertransversarius.dorsalis.3.right, intertransversarius.ventralis.1.right, intertransversarius.ventralis.2.right, intertransversarius.ventralis.3.right
5	0.92103	scapula.left, serratus.ventralis.left, omohyoideus.left, rhomboideus.anticus.left, rhomboideus.capitis.left

Table A37 Connectivity modules identified for *Notoryctes typhlops*.

ID	p-value	Elements
1	0.02926	cranium, C1, longus.capitis.left, longus.capitis.right, obliquus.capitis.cranialis.left, obliquus.capitis.cranialis.right, rectus.capitis.dorsalis.major.left, rectus.capitis.dorsalis.major.right, rectus.capitis.dorsalis.minor.left, rectus.capitis.dorsalis.minor.right, rectus.capitis.dorsalis.superficialis.left, rectus.capitis.dorsalis.superficialis.right, rectus.capitis.lateralis.left, rectus.capitis.lateralis.right
2	0.29166	clavicle.left, scapula.left, humerus.left, acromiotrapezius.left, cleidooccipitalis.left, omohyoideus.left, rhomboideus.left
3	0.03674	C2, C3, C4, complexus.left, complexus.right, longissimus.cervicis.left, longissimus.cervicis.right, obliquus.capitis.caudalis.left, obliquus.capitis.caudalis.right, scalenus.medius.left, scalenus.medius.right, splenius..left, splenius.right, interspinalis.1, interspinalis.2, multifidius.submultifidius.1.left, multifidius.submultifidius.2.left, multifidius.submultifidius.3.left, intertransversarii.dorsalis.1.left, intertransversarii.dorsalis.2.left, intertransversarii.ventralis.1.left, intertransversarii.ventralis.2.left, spinalis.cervicis.left, multifidius.submultifidius.1.right, multifidius.submultifidius.2.right, multifidius.submultifidius.3.right, intertransversarii.dorsalis.1.right, intertransversarii.dorsalis.2.right, intertransversarii.ventralis.1.right, intertransversarii.ventralis.2.right, spinalis.cervicis.right
4	0.00174	clavicle.right, scapula.right, sternum, hyoid, thyroid, humerus.right, cleidooccipitalis.right, omohyoideus.right, rhomboideus.right, sternohyoideus.left, sternohyoideus.right, sternomastoideus.left, sternomastoideus.right, sternothyroideus.left, sternothyroideus.right
5	0.3078	C5, C6, scalenus.posticus.left, scalenus.posticus.right, semispinalis.cervicis.right, serratus.magnus.left, serratus.magnus.right, interspinalis.3, interspinalis.4, multifidius.submultifidius.4.left, multifidius.submultifidius.6.left, intertransversarii.dorsalis.3.left, intertransversarii.dorsalis.4.left, intertransversarii.dorsalis.5.left, intertransversarii.ventralis.3.left, intertransversarii.ventralis.4.left, multifidius.submultifidius.4.right, multifidius.submultifidius.5.right, intertransversarii.dorsalis.3.right, intertransversarii.dorsalis.4.right, intertransversarii.ventralis.3.right, intertransversarii.ventralis.4.right
6	0.09564	C7, thoracic.spine, ribs.left, ribs.right, acromiotrapezius.right, iliocostalis.dorsi.left, iliocostalis.dorsi.right, longissimus.capitis.left, longissimus.capitis.right, longus.colli.left, longus.colli.right, semispinalis.cervicis.left, interspinalis.5, interspinalis.6, multifidius.submultifidius.5.left, intertransversarii.dorsalis.6.left, intertransversarii.ventralis.5.left, intertransversarii.ventralis.6.left, multifidius.submultifidius.6.right, intertransversarii.dorsalis.5.right, intertransversarii.dorsalis.6.right, intertransversarii.ventralis.5.right, intertransversarii.ventralis.6.right

Table A38 Connectivity modules identified for *Ornithorhynchus anatinus*.

ID	p-value	Elements
1	0.15264	C2, C3, intertransversarii.dorsalis.cervicis.left, intertransversarii.dorsalis.cervicis.right, obliquus.capitis.caudalis.left, obliquus.capitis.caudalis.right, scalenus.left, scalenus.right, spinalis.cervicis.left, spinalis.cervicis.right, intertransversarius.ventralis.2.left, intertransversarius.ventralis.3.left, intertransversarius.ventralis.2.right, intertransversarius.ventralis.3.right
2	0.00021	C4, C5, C6, C7, thoracic.spine, complexus.major.left, complexus.major.right, iliocostalis.cervicis.left, iliocostalis.cervicis.right, longissimus.capitis.left, longissimus.capitis.right, longissimus.cervicis.left, longissimus.cervicis.right, longus.colli.left, longus.colli.right, rhomboideus.left, rhomboideus.right, serratus.ventralis.cervicis.left, serratus.ventralis.cervicis.right, splenius.left, splenius.right, intertransversarius.ventralis.4.left, intertransversarius.ventralis.5.left, intertransversarius.ventralis.6.left, intertransversarius.ventralis.7.left, intertransversarius.ventralis.4.right, intertransversarius.ventralis.5.right, intertransversarius.ventralis.6.right, intertransversarius.ventralis.7.right
3	0.5	scapula.left, clavicula.left, levator.scapulae.dorsalis.left, levator.scapulae.ventralis.left, omohyoid.left, sterno.cleido.mastoid.left, trapezius.anterior.left
4	0.00196	scapula.right, sternum, hyoid, thyroid, ribs.left, ribs.right, clavicula.right, levator.scapulae.dorsalis.right, levator.scapulae.ventralis.right, omohyoid.right, sterno.cleido.mastoid.right, sternohyoid.left, sternohyoid.right, sternothyroid.left, sternothyroid.right, trapezius.anterior.right
5	0.06848	cranium, C1, biventer.cervicis.left, biventer.cervicis.right, longus.capitis.left, longus.capitis.right, rectus.capitis.dorsalis.major.left, rectus.capitis.dorsalis.major.right, rectus.capitis.dorsalis.minor.left, rectus.capitis.dorsalis.minor.right, rectus.capitis.lateralis.brevis.left, rectus.capitis.lateralis.brevis.right, rectus.capitis.lateralis.longus.left, rectus.capitis.lateralis.longus.right, rectus.capitis.ventralis.left, rectus.capitis.ventralis.right, intertransversarius.ventralis.1.left, intertransversarius.ventralis.1.right

Table A39 Connectivity modules identified for *Orycteropus afer*.

ID	p-value	Elements
1	0.03997	C6, C7, thoracic.spine, ribs.left, iliocostalis.cervicis.left, iliocostalis.cervicis.right, longissimus.cervicis.left, longissimus.cervicis.right, longus.colli.left, longus.colli.right, semispinalis.capitis.left, semispinalis.capitis.right, semispinalis.cervicis.left, semispinalis.cervicis.right, splenius.capitis.left, splenius.capitis.right, interspinalis.5, interspinalis.6, intertransversarius.dorsalis.5.left, intertransversarius.dorsalis.6.left, intertransversarius.ventralis.5.left, intertransversarius.ventralis.6.left, intertransversarius.dorsalis.5.right, intertransversarius.dorsalis.6.right, intertransversarius.ventralis.5.right, intertransversarius.ventralis.6.right
2	1e-05	cranium, C1, clavicle.left, clavicle.right, scapula.left, scapula.right, nuchal.ligament, clavotrapezius.left, clavotrapezius.right, cleido.mastoideus.left, cleido.mastoideus.right, obliquus.capitis.caudalis.right, obliquus.capitis.cranialis.left, obliquus.capitis.cranialis.right, rectus.capitis.dorsalis.medius.left, rectus.capitis.dorsalis.medius.right, rectus.capitis.dorsalis.minor.left, rectus.capitis.dorsalis.minor.right, rectus.capitis.lateralis.left, rectus.capitis.lateralis.right, rhomboideus.cervicis.left, rhomboideus.cervicis.right, trapezius.left, trapezius.right
3	0.06344	C4, C5, longissimus.capitis.left, longissimus.capitis.right, longus.capitis.left, scalenus.brevis.left, scalenus.brevis.right, serratus.ventralis.cervicis.left, interspinalis.3, interspinalis.4, intertransversarius.dorsalis.2.left, intertransversarius.dorsalis.3.left, intertransversarius.dorsalis.4.left, intertransversarius.ventralis.2.left, intertransversarius.ventralis.3.left, intertransversarius.ventralis.4.left, intertransversarius.dorsalis.2.right, intertransversarius.dorsalis.3.right, intertransversarius.dorsalis.4.right, intertransversarius.ventralis.3.right, intertransversarius.ventralis.4.right
4	0.00868	sternum, hyoid, thyroid, ribs.right, sternohyoideus.left, sternohyoideus.right, sterno.mastoideus.left, sterno.mastoideus.right, sternothyroideus.left, sternothyroideus.right
5	0.01016	C2, C3, levator.claviculae.left, levator.claviculae.right, levator.scapulae.left, levator.scapulae.right, longus.capitis.right, obliquus.capitis.caudalis.left, rectus.capitis.dorsalis.major.left, rectus.capitis.dorsalis.major.right, scalenus.longus.left, scalenus.longus.right, serratus.ventralis.cervicis.right, interspinalis.1, interspinalis.2, intertransversarius.dorsalis.1.left, intertransversarius.ventralis.1.left, intertransversarius.dorsalis.1.right, intertransversarius.ventralis.1.right, intertransversarius.ventralis.2.right

Table A40 Connectivity modules identified for *Oryctolagus cuniculus*.

ID	p-value	Elements
1	0.08183	C2, C3, longus.capitis.right, obliquus.capitis.caudalis.left, obliquus.capitis.caudalis.right, rectus.capitis.dorsalis.superficialis.left, rhomboideus.right, semispinalis.capitis.right, splenius.left, splenius.right, trapezius.left, interspinalis.1, interspinalis.2, multifidius.submultifidius.1.left, multifidius.submultifidius.2.left, intertransversarius.dorsalis.1.left, intertransversarius.dorsalis.2.left, intertransversarius.ventralis.1.left, intertransversarius.ventralis.2.left, multifidius.submultifidius.1.right, multifidius.submultifidius.2.right, intertransversarius.dorsalis.1.right, intertransversarius.dorsalis.2.right, intertransversarius.ventralis.1.right, intertransversarius.ventralis.2.right
2	0.00124	sternum, hyoid, thyroid, ribs.left, iliocostalis.cervicis.left, scalenus.medius.left, sternohyoideus.left, sternohyoideus.right, sternomastoideus.left, sternomastoideus.right, sternothyroideus.left, sternothyroideus.right
3	0	cranium, C1, humerus.left, humerus.right, scapula.left, scapula.right, cleidomastoideus.left, cleidomastoideus.right, cleidooccipitalis.left, cleidooccipitalis.right, levator.claviculae.left, levator.claviculae.right, levator.scapulae.left, levator.scapulae.right, longissimus.capitis.left, longissimus.capitis.right, longus.capitis.left, obliquus.capitis.cranialis.left, obliquus.capitis.cranialis.right, rectus.capitis.dorsalis.major.left, rectus.capitis.dorsalis.major.right, rectus.capitis.dorsalis.minor.left, rectus.capitis.dorsalis.minor.right, rectus.capitis.dorsalis.superficialis.right, rectus.capitis.lateralis.left, rectus.capitis.lateralis.right, rectus.capitis.ventralis.left, rectus.capitis.ventralis.right, semispinalis.capitis.left
4	0.05301	C4, C5, ribs.right, iliocostalis.cervicis.right, longus.atlantis.left, longus.atlantis.right, scalenus.anticus.left, scalenus.anticus.right, scalenus.medius.right, scalenus.tertius.left, scalenus.tertius.right, serratus.ventralis.left, serratus.ventralis.right, interspinalis.3, interspinalis.4, multifidius.submultifidius.3.left, multifidius.submultifidius.4.left, intertransversarius.dorsalis.3.left, intertransversarius.dorsalis.4.left, intertransversarius.ventralis.3.left, intertransversarius.ventralis.4.left, multifidius.submultifidius.3.right, multifidius.submultifidius.4.right, intertransversarius.dorsalis.3.right, intertransversarius.dorsalis.4.right, intertransversarius.ventralis.3.right, intertransversarius.ventralis.4.right
5	0.04779	C6, C7, thoracic.spine, longissimus.cervicis.left, longissimus.cervicis.right, longus.colli.left, longus.colli.right, rhomboideus.left, semispinalis.cervicis.left, semispinalis.cervicis.right, spinalis.cervicis.left, spinalis.cervicis.right, splenius.capitis.left, splenius.capitis.right, trapezius.right, interspinalis.5, interspinalis.6, multifidius.submultifidius.5.left, multifidius.submultifidius.6.left, intertransversarius.dorsalis.5.left, intertransversarius.dorsalis.6.left, intertransversarius.ventralis.5.left, intertransversarius.ventralis.6.left, multifidius.submultifidius.5.right, multifidius.submultifidius.6.right, intertransversarius.dorsalis.5.right, intertransversarius.dorsalis.6.right, intertransversarius.ventralis.5.right, intertransversarius.ventralis.6.right

Table A41 Connectivity modules identified for *Pedetes capensis*.

ID	p-value	Elements
1	0.65572	clavicle.left, scapula.left, cleidomastoideus.left, cleidotrapezius.left, levator.claviculae.left, rhomboideus.anticus.left, trapezius.left
2	0.00014	cranium, C1, clavicle.right, scapula.right, cleidomastoideus.right, cleidotrapezius.right, levator.claviculae.right, obliquus.capitis.caudalis.left, obliquus.capitis.caudalis.right, obliquus.capitis.cranialis.left, obliquus.capitis.cranialis.right, rectus.capitis.dorsalis.major.left, rectus.capitis.dorsalis.major.right, rectus.capitis.dorsalis.minor.left, rectus.capitis.dorsalis.minor.right, rectus.capitis.dorsalis.superficialis.left, rectus.capitis.dorsalis.superficialis.right, rectus.capitis.lateralis.left, rectus.capitis.lateralis.right, rectus.capitis.ventralis.left, rectus.capitis.ventralis.right, rhomboideus.anticus.right, splenius.cervicis.left, splenius.cervicis.right, trapezius.right
3	0.01531	sternum, hyoid, thyroid, ribs.left, ribs.right, sternohyoideus.left, sternohyoideus.right, sternomastoideus.left, sternomastoideus.right, sternothyroideus.left, sternothyroideus.right
4	0.01957	C6, C7, thoracic.spine, iliocostalis.cervicis.left, iliocostalis.cervicis.right, longissimus.cervicis.left, longissimus.cervicis.right, longus.colli.left, longus.colli.right, semispinalis.spinalis.cervicis.left, semispinalis.spinalis.cervicis.left.1, splenius.left, splenius.right, interspinalis.5, interspinalis.6, multifidius.submultifidius.3.left, multifidius.submultifidius.5.left, multifidius.submultifidius.6.left, intertransversarius.dorsalis.5.left, intertransversarius.dorsalis.6.left, intertransversarius.ventralis.5.left, intertransversarius.ventralis.6.left, multifidius.submultifidius.3.right, multifidius.submultifidius.5.right, multifidius.submultifidius.6.right, intertransversarius.dorsalis.5.right, intertransversarius.dorsalis.6.right, intertransversarius.ventralis.5.right, intertransversarius.ventralis.6.right
5	0.02471	C2, C3, C4, longus.capitis.left, longus.capitis.right, scalenus.medius.left, scalenus.medius.right, scalenus.tertius.left, scalenus.tertius.right, semispinalis.capitis.left, semispinalis.capitis.right, serratus.ventralis.left, serratus.ventralis.right, interspinalis.1, interspinalis.2, multifidius.submultifidius.1.left, intertransversarius.dorsalis.1.left, intertransversarius.dorsalis.2.left, intertransversarius.ventralis.1.left, intertransversarius.ventralis.2.left, multifidius.submultifidius.1.right, intertransversarius.dorsalis.1.right, intertransversarius.dorsalis.2.right, intertransversarius.ventralis.1.right, intertransversarius.ventralis.2.right
6	0.5382	C5, interspinalis.3, interspinalis.4, multifidius.submultifidius.2.left, multifidius.submultifidius.4.left, intertransversarius.dorsalis.3.left, intertransversarius.dorsalis.4.left, intertransversarius.ventralis.3.left, intertransversarius.ventralis.4.left, multifidius.submultifidius.2.right, multifidius.submultifidius.4.right, intertransversarius.dorsalis.3.right, intertransversarius.dorsalis.4.right, intertransversarius.ventralis.3.right, intertransversarius.ventralis.4.right

Table A42 Connectivity modules identified for *Phascolarctos cinereus*.

ID	p-value	Elements
1	0	cranium, mandible, C1, clavicle.left, clavicle.right, scapula.left, scapula.right, sternum, ribs.right, atlantoscapularis.left, atlantoscapularis.right, biventer.cervicis.left, biventer.cervicis.right, cleidomastoideus.left, cleidomastoideus.right, cleidooccipitalis.left, cleidooccipitalis.right, obliquus.capitis.cranialis.left, obliquus.capitis.cranialis.right, omohyoideus.left, omohyoideus.right, rectus.capitis.dorsalis.major.left, rectus.capitis.dorsalis.major.right, rectus.capitis.dorsalis.minor.left, rectus.capitis.dorsalis.minor.right, rectus.capitis.dorsalis.superficialis.left, rectus.capitis.dorsalis.superficialis.right, rectus.capitis.lateralis.left, rectus.capitis.lateralis.right, rhomboideus.left, rhomboideus.right, sternohyoideus.left, sternohyoideus.right, sternomastoideus.left, sternomastoideus.right, trapezius.left, trapezius.right
2	0.00236	C2, C3, C4, longissimus.cervicis.left, longus.capitis.left, longus.capitis.right, obliquus.capitis.caudalis.left, obliquus.capitis.caudalis.right, scalenus.posticus.left, scalenus.posticus.right, serratus.magnus.left, serratus.magnus.right, interspinalis.1, interspinalis.2, interspinalis.3, multifidius.submultifidius.1.left, multifidius.submultifidius.2.left, multifidius.submultifidius.3.left, intertransversarii.dorsalis.1.left, intertransversarii.dorsalis.2.left, intertransversarii.dorsalis.3.left, intertransversarii.ventralis.1.left, intertransversarii.ventralis.2.left, intertransversarii.ventralis.3.left, multifidius.submultifidius.1.right, multifidius.submultifidius.2.right, multifidius.submultifidius.3.right, intertransversarii.dorsalis.1.right, intertransversarii.dorsalis.2.right, intertransversarii.dorsalis.3.right, intertransversarii.ventralis.1.right, intertransversarii.ventralis.2.right, intertransversarii.ventralis.3.right
3	0.74991	ribs.left, iliocostalis.dorsi.right, longus.colli.right
4	2e-05	C5, C6, C7, thoracic.spine, complexus.left, complexus.right, longissimus.capitis.left, longissimus.capitis.right, longissimus.cervicis.right, scalenus.medius.left, scalenus.medius.right, semispinalis.cervicis.left, semispinalis.cervicis.right, splenius.capitis.left, splenius.capitis.right, splenius.cervicis.left, splenius.cervicis.right, interspinalis.4, interspinalis.5, interspinalis.6, multifidius.submultifidius.4.left, multifidius.submultifidius.5.left, multifidius.submultifidius.6.left, intertransversarii.dorsalis.4.left, intertransversarii.dorsalis.5.left, intertransversarii.dorsalis.6.left, intertransversarii.ventralis.4.left, intertransversarii.ventralis.5.left, intertransversarii.ventralis.6.left, spinalis.cervicis.left, multifidius.submultifidius.4.right, multifidius.submultifidius.5.right, multifidius.submultifidius.6.right, intertransversarii.dorsalis.4.right, intertransversarii.dorsalis.5.right, intertransversarii.dorsalis.6.right, intertransversarii.ventralis.4.right, intertransversarii.ventralis.5.right, intertransversarii.ventralis.6.right, spinalis.cervicis.right
5	0.87411	hyoid, thyroid, iliocostalis.dorsi.left, longus.colli.left, sternothyroideus.left, sternothyroideus.right

Table A43 Connectivity modules identified for *Procavia capensis*.

ID	p-value	Elements
1	0.00026	cranium, C1, scapula.left, humerus.left, brachiocephalicus.left, levator.claviculae.left, levator.scapulae.left, obliquus.capitis.caudalis.left, obliquus.capitis.caudalis.right, obliquus.capitis.cranialis.left, obliquus.capitis.cranialis.right, rectus.capitis.dorsalis.major.left, rectus.capitis.dorsalis.major.right, rectus.capitis.dorsalis.minor.left, rectus.capitis.dorsalis.minor.right, rectus.capitis.lateralis.left, rectus.capitis.lateralis.right, rhomboideus.capitis.left, splenius.capitis.left, splenius.capitis.right, splenius.cervicis.left, splenius.cervicis.right, trapezius.left
2	0.40414	scapula.right, humerus.right, brachiocephalicus.right, levator.claviculae.right, levator.scapulae.right, rhomboideus.capitis.right, rhomboideus.cervicis.right, serratus.ventralis.right, trapezius.right
3	1e-04	mandible, sternum, hyoid, thyroid, cleidomastoideus.left, cleidomastoideus.right, sternohyoid.left, sternohyoid.right, sternomaxillaris.left, sternomaxillaris.right, sternothyroid.left, sternothyroid.right
4	0.06012	C2, C3, C4, complexus.major.left, complexus.tertius.left, complexus.tertius.right, longus.colli.left, longus.colli.right, rhomboideus.cervicis.left, interspinalis.1, interspinalis.2, multifidius.submultifidius.1.left, multifidius.submultifidius.2.left, multifidius.submultifidius.3.left, intertransversarius.dorsalis.1.left, intertransversarius.dorsalis.2.left, intertransversarius.ventralis.1.left, intertransversarius.ventralis.2.left, spinalis.cervicis.left, multifidius.submultifidius.1.right, multifidius.submultifidius.2.right, multifidius.submultifidius.3.right, intertransversarius.dorsalis.1.right, intertransversarius.dorsalis.2.right, intertransversarius.ventralis.1.right, intertransversarius.ventralis.2.right, spinalis.cervicis.right
5	0.0413	C5, C6, ribs.left, ribs.right, longus.capitis.left, longus.capitis.right, scalenus.anticus.left, scalenus.anticus.right, scalenus.posticus.left, scalenus.posticus.right, serratus.ventralis.left, interspinalis.3, interspinalis.4, multifidius.submultifidius.4.left, intertransversarius.dorsalis.3.left, intertransversarius.dorsalis.4.left, intertransversarius.ventralis.3.left, intertransversarius.ventralis.4.left, multifidius.submultifidius.4.right, intertransversarius.dorsalis.3.right, intertransversarius.dorsalis.4.right, intertransversarius.ventralis.3.right, intertransversarius.ventralis.4.right
6	0.15363	C7, thoracic.spine, complexus.major.right, longissimus.capitis.left, longissimus.capitis.right, longissimus.cervicis.left, longissimus.cervicis.right, interspinalis.5, interspinalis.6, multifidius.submultifidius.5.left, multifidius.submultifidius.6.left, intertransversarius.dorsalis.5.left, intertransversarius.dorsalis.6.left, intertransversarius.ventralis.5.left, intertransversarius.ventralis.6.left, multifidius.submultifidius.5.right, multifidius.submultifidius.6.right, intertransversarius.dorsalis.5.right, intertransversarius.dorsalis.6.right, intertransversarius.ventralis.5.right, intertransversarius.ventralis.6.right

Table A44 Connectivity modules identified for *Pteropus vampyrus*.

ID	p-value	Elements
1	0	C5, C6, C7, thoracic.spine, levator.scapulae.left, levator.scapulae.right, scalenus.posterior.left, scalenus.posterior.right, semispinalis.capitis.left, semispinalis.capitis.right, semispinalis.cervicis.left, semispinalis.cervicis.right, splenius.capitis.left, splenius.capitis.right, multifidius.submultifidius.4.left, multifidius.submultifidius.5.left, multifidius.submultifidius.6.left, intertransversarius.dorsalis.4.left, intertransversarius.dorsalis.5.left, intertransversarius.dorsalis.6.left, intertransversarius.ventralis.4.left, intertransversarius.ventralis.5.left, intertransversarius.ventralis.6.left, multifidius.submultifidius.4.right, multifidius.submultifidius.5.right, multifidius.submultifidius.6.right, intertransversarius.dorsalis.4.right, intertransversarius.dorsalis.5.right, intertransversarius.dorsalis.6.right, intertransversarius.ventralis.4.right, intertransversarius.ventralis.5.right, intertransversarius.ventralis.6.right
2	0.12745	cranium, C1, longus.capitis.left, longus.capitis.right, obliquus.capitis.caudalis.left, obliquus.capitis.caudalis.right, rectus.capitis.dorsalis.major.left, rectus.capitis.dorsalis.major.right, rectus.capitis.dorsalis.minor.left, rectus.capitis.dorsalis.minor.right, rectus.capitis.lateralis.left, rectus.capitis.lateralis.right, sternomastoideus.right
3	0.97939	ribs.left, scalenus.medius.left
4	3e-04	C2, C3, C4, cervico.clavicularis.left, cervico.clavicularis.right, intertransversus.lateralis.left, intertransversus.lateralis.right, longus.colli.left, longus.colli.right, scalenus.medius.right, multifidius.submultifidius.1.left, multifidius.submultifidius.2.left, multifidius.submultifidius.3.left, intertransversarius.dorsalis.1.left, intertransversarius.dorsalis.2.left, intertransversarius.dorsalis.3.left, intertransversarius.ventralis.1.left, intertransversarius.ventralis.2.left, intertransversarius.ventralis.3.left, multifidius.submultifidius.1.right, multifidius.submultifidius.2.right, multifidius.submultifidius.3.right, intertransversarius.dorsalis.1.right, intertransversarius.dorsalis.2.right, intertransversarius.dorsalis.3.right, intertransversarius.ventralis.1.right, intertransversarius.ventralis.2.right, intertransversarius.ventralis.3.right
5	7e-05	clavicle.left, clavicle.right, scapula.left, scapula.right, sternum, hyoid, thyroid, ribs.right, cleidomastoideus.left, cleidomastoideus.right, omohyoideus.left, omohyoideus.right, sternohyoideus.left, sternohyoideus.right, sternomastoideus.left, sternothyroideus.left, sternothyroideus.right

Table A45 Connectivity modules identified for *Ptilocercus lowii*.

ID	p-value	Elements
1	0.04718	sternum, thyroid, ribs.left, ribs.right, scalenus.medius.left, sternohyoideus.left, sternohyoideus.right, sternomastoideus.left, sternomastoideus.right, sternothyroideus.left, sternothyroideus.right
2	7e-04	C2, C3, C4, levator.anguli.scapulae.left, levator.anguli.scapulae.right, rectus.capitis.dorsalis.superficialis.left, rectus.capitis.dorsalis.superficialis.right, scalenus.medius.right, scalenus.posticus.left, scalenus.posticus.right, trapezius.right, interspinalis.1, interspinalis.2, interspinalis.3, multifidius.submultifidius.1.left, multifidius.submultifidius.2.left, multifidius.submultifidius.3.left, intertransversarius.dorsalis.1.left, intertransversarius.dorsalis.2.left, intertransversarius.dorsalis.3.left, intertransversarius.ventralis.1.left, intertransversarius.ventralis.2.left, intertransversarius.ventralis.3.left, multifidius.submultifidius.1.right, multifidius.submultifidius.2.right, multifidius.submultifidius.3.right, intertransversarius.dorsalis.1.right, intertransversarius.dorsalis.2.right, intertransversarius.dorsalis.3.right, intertransversarius.ventralis.1.right, intertransversarius.ventralis.2.right, intertransversarius.ventralis.3.right
3	0.00011	C5, C6, C7, thoracic.spine, complexus.left, complexus.right, longissimus.capitis.left, longissimus.capitis.right, longissimus.cervicis.left, longissimus.cervicis.right, longus.capitis.left, longus.capitis.right, longus.colli.left, longus.colli.right, semispinalis.cervicis.left, semispinalis.cervicis.right, splenius.capitis.left, splenius.capitis.right, interspinalis.4, interspinalis.5, interspinalis.6, multifidius.submultifidius.4.left, multifidius.submultifidius.5.left, multifidius.submultifidius.6.left, intertransversarius.dorsalis.4.left, intertransversarius.dorsalis.5.left, intertransversarius.dorsalis.6.left, intertransversarius.ventralis.4.left, intertransversarius.ventralis.5.left, intertransversarius.ventralis.6.left, multifidius.submultifidius.4.right, multifidius.submultifidius.5.right, multifidius.submultifidius.6.right, intertransversarius.dorsalis.4.right, intertransversarius.dorsalis.5.right, intertransversarius.dorsalis.6.right, intertransversarius.ventralis.4.right, intertransversarius.ventralis.5.right, intertransversarius.ventralis.6.right
4	0	cranium, C1, clavicle.left, clavicle.right, scapula.left, scapula.right, hyoid, cleidomastoideus.left, cleidomastoideus.right, cleidooccipitalis.left, cleidooccipitalis.right, levator.claviculae.left, levator.claviculae.right, levator.scapulae.left, levator.scapulae.right, obliquus.capitis.caudalis.left, obliquus.capitis.caudalis.right, obliquus.capitis.cranialis.left, obliquus.capitis.cranialis.right, omohyoideus.left, omohyoideus.right, rectus.capitis.dorsalis.major.left, rectus.capitis.dorsalis.major.right, rectus.capitis.dorsalis.minor.left, rectus.capitis.dorsalis.minor.right, rectus.capitis.lateralis.left, rectus.capitis.lateralis.right, rectus.capitis.ventralis.left, rectus.capitis.ventralis.right, rhomboideus.capitis.left, rhomboideus.capitis.right, trapezius.left

Table A46 Connectivity modules identified for *Sarcophilus harrisii*.

ID	p-value	Elements
1	1e-05	C5, C6, C7, thoracic.spine, ribs.left, complexus.left, iliocostalis.dorsi.left, longissimus.capitis.left, longissimus.capitis.right, longus.colli.left, rhomboideus.left, rhomboideus.right, scalenus.posticus.left, semispinalis.cervicis.left, semispinalis.cervicis.right, serratus.magnus.left, serratus.magnus.right, interspinalis.4, interspinalis.5, interspinalis.6, multifidius.submultifidius.4.left, multifidius.submultifidius.5.left, multifidius.submultifidius.6.left, intertransversarii.dorsalis.4.left, intertransversarii.dorsalis.5.left, intertransversarii.dorsalis.6.left, intertransversarii.ventralis.4.left, intertransversarii.ventralis.5.left, intertransversarii.ventralis.6.left, spinalis.cervicis.left, multifidius.submultifidius.4.right, multifidius.submultifidius.5.right, multifidius.submultifidius.6.right, intertransversarii.dorsalis.4.right, intertransversarii.dorsalis.5.right, intertransversarii.dorsalis.6.right, intertransversarii.ventralis.4.right, intertransversarii.ventralis.5.right, intertransversarii.ventralis.6.right, spinalis.cervicis.right
2	0.09383	cranium, C1, longus.capitis.left, longus.capitis.right, obliquus.capitis.cranialis.left, obliquus.capitis.cranialis.right, rectus.capitis.dorsalis.major.left, rectus.capitis.dorsalis.major.right, rectus.capitis.dorsalis.minor.left, rectus.capitis.dorsalis.minor.right, rectus.capitis.dorsalis.superficialis.left, rectus.capitis.dorsalis.superficialis.right, rectus.capitis.lateralis.left, rectus.capitis.lateralis.right, splenius..left, splenius.right
3	0.0071	sternum, hyoid, thyroid, omohyoideus.left, sternohyoideus.left, sternohyoideus.right, sternomastoideus.left, sternomastoideus.right, sternothyroideus.left, sternothyroideus.right
4	0.14549	clavicle.right, scapula.right, humerus.right, atlantoscapularis.dorsalis.right, atlantoscapularis.right, cleidomastoideus.right, omohyoideus.right, rhomboideus.capitis.right, trapezius.right
5	0.19186	clavicle.left, scapula.left, humerus.left, atlantoscapularis.dorsalis.left, atlantoscapularis.left, cleidomastoideus.left, rhomboideus.capitis.left, trapezius.left
6	0.00064	C2, C3, C4, complexus.right, longissimus.cervicis.left, longissimus.cervicis.right, obliquus.capitis.caudalis.left, obliquus.capitis.caudalis.right, scalenus.medius.left, scalenus.medius.right, interspinalis.1, interspinalis.2, interspinalis.3, multifidius.submultifidius.1.left, multifidius.submultifidius.2.left, multifidius.submultifidius.3.left, intertransversarii.dorsalis.1.left, intertransversarii.dorsalis.2.left, intertransversarii.dorsalis.3.left, intertransversarii.ventralis.1.left, intertransversarii.ventralis.2.left, intertransversarii.ventralis.3.left, multifidius.submultifidius.1.right, multifidius.submultifidius.2.right, multifidius.submultifidius.3.right, intertransversarii.dorsalis.1.right, intertransversarii.dorsalis.2.right, intertransversarii.dorsalis.3.right, intertransversarii.ventralis.1.right, intertransversarii.ventralis.2.right, intertransversarii.ventralis.3.right
7	0.97573	ribs.right, iliocostalis.dorsi.right, longus.colli.right, scalenus.posticus.right

Table A47 Connectivity modules identified for *Scalopus aquaticus*.

ID	p-value	Elements
1	0.00031	C2, C3, C4, longissimus.capitis.left, longissimus.capitis.right, longus.atlantis.left, longus.atlantis.right, longus.colli.left, longus.colli.right, scalenus.b.left, scalenus.b.right, splenius.left, multifidius.submultifidius.1.left, multifidius.submultifidius.2.left, multifidius.submultifidius.3.left, intertransversarius.dorsalis.1.left, intertransversarius.dorsalis.2.left, intertransversarius.dorsalis.3.left, intertransversarius.ventralis.1.left, intertransversarius.ventralis.2.left, intertransversarius.ventralis.3.left, multifidius.submultifidius.1.right, multifidius.submultifidius.2.right, multifidius.submultifidius.3.right, intertransversarius.dorsalis.1.right, intertransversarius.dorsalis.2.right, intertransversarius.dorsalis.3.right, intertransversarius.ventralis.1.right, intertransversarius.ventralis.2.right, intertransversarius.ventralis.3.right
2	0	cranium, C1, clavicle.left, clavicle.right, scapula.left, scapula.right, cleido.mastoideus.left, cleido.mastoideus.right, cleido.occipitalis.left, cleido.occipitalis.right, levator.claviculae.left, levator.claviculae.right, levator.scapulae.left, levator.scapulae.right, obliquus.capitis.caudalis.left, obliquus.capitis.caudalis.right, obliquus.capitis.cranialis.left, obliquus.capitis.cranialis.right, rectus.capitis.dorsalis.major.left, rectus.capitis.dorsalis.major.right, rectus.capitis.dorsalis.minor.left, rectus.capitis.dorsalis.minor.right, rectus.capitis.ventralis.left, rectus.capitis.ventralis.right, rhomboideus.capitis.left, rhomboideus.capitis.right
3	0.0055	sternum, hyoid, thyroid, ribs.left, ribs.right, scalenus.a.left, scalenus.a.right, sternohyoideus.left, sternohyoideus.right, sterno.mastoideus.left, sterno.mastoideus.right, sterno.occipitalis.left, sterno.occipitalis.right, sternothyroideus.left, sternothyroideus.right
4	0.00013	C5, C6, C7, thoracic.spine, longissimus.cervicis.left, longissimus.cervicis.right, longus.capitis.left, longus.capitis.right, rhomboideus.cervicis.left, rhomboideus.cervicis.right, semispinalis.capitis.left, semispinalis.capitis.right, semispinalis.cervicis.left, semispinalis.cervicis.right, serratus.ventralis.cervicis.left, serratus.ventralis.cervicis.right, splenius.right, trapezius.anticus.left, trapezius.anticus.right, multifidius.submultifidius.4.left, multifidius.submultifidius.5.left, multifidius.submultifidius.6.left, intertransversarius.dorsalis.4.left, intertransversarius.dorsalis.5.left, intertransversarius.dorsalis.6.left, intertransversarius.ventralis.4.left, intertransversarius.ventralis.5.left, intertransversarius.ventralis.6.left, multifidius.submultifidius.4.right, multifidius.submultifidius.5.right, multifidius.submultifidius.6.right, intertransversarius.dorsalis.4.right, intertransversarius.dorsalis.5.right, intertransversarius.dorsalis.6.right, intertransversarius.ventralis.4.right, intertransversarius.ventralis.5.right, intertransversarius.ventralis.6.right

Table A48 Connectivity modules identified for *Sciurus vulgaris*.

ID	p-value	Elements
1	1e-05	cranium, C1, C2, scapula.left, biventer.cervicis.left, biventer.cervicis.right, levator.claviculae.left, levator.scapulae.left, obliquus.capitis.caudalis.left, obliquus.capitis.caudalis.right, obliquus.capitis.cranialis.left, obliquus.capitis.cranialis.right, rectus.capitis.dorsalis.major.left, rectus.capitis.dorsalis.major.right, rectus.capitis.dorsalis.minor.left, rectus.capitis.dorsalis.minor.right, rectus.capitis.dorsalis.superficialis.left, rectus.capitis.dorsalis.superficialis.right, rectus.capitis.lateralis.left, rectus.capitis.lateralis.right, rectus.capitis.ventralis.left, rectus.capitis.ventralis.right, rhomboideus.capitis.left, rhomboideus.left, splenius.left, splenius.right, trapezius.left, interspinalis.1, multifidius.submultifidius.1.left, intertransversarius.dorsalis.1.left, intertransversarius.ventralis.1.left, multifidius.submultifidius.1.right, intertransversarius.dorsalis.1.right, intertransversarius.ventralis.1.right
2	0	C6, C7, thoracic.spine, ribs.left, ribs.right, complexus.left, complexus.right, iliocostalis.cervicis.left, iliocostalis.cervicis.right, longissimus.capitis.left, longissimus.capitis.right, longissimus.cervicis.left, longissimus.cervicis.right, longus.atlantis.d.left, longus.atlantis.d.right, scalenus.tertius.left, scalenus.tertius.right, semispinalis.cervicis.left, semispinalis.cervicis.right, spinalis.cervicis.left, spinalis.cervicis.right, splenius.capitis.left, splenius.capitis.right, interspinalis.5, interspinalis.6, multifidius.submultifidius.5.left, multifidius.submultifidius.6.left, intertransversarius.dorsalis.5.left, intertransversarius.dorsalis.6.left, intertransversarius.ventralis.5.left, intertransversarius.ventralis.6.left, multifidius.submultifidius.5.right, multifidius.submultifidius.6.right, intertransversarius.dorsalis.5.right, intertransversarius.dorsalis.6.right, intertransversarius.ventralis.5.right, intertransversarius.ventralis.6.right
3	0.00056	C3, C4, C5, longus.atlantis.v.left, longus.atlantis.v.right, longus.capitis.left, longus.capitis.right, longus.colli.left, longus.colli.right, scalenus.anticus.left, scalenus.anticus.right, scalenus.medius.left, scalenus.medius.right, serratus.ventralis.left, interspinalis.2, interspinalis.3, interspinalis.4, multifidius.submultifidius.2.left, multifidius.submultifidius.3.left, multifidius.submultifidius.4.left, intertransversarius.dorsalis.2.left, intertransversarius.dorsalis.3.left, intertransversarius.dorsalis.4.left, intertransversarius.ventralis.2.left, intertransversarius.ventralis.3.left, intertransversarius.ventralis.4.left, multifidius.submultifidius.2.right, multifidius.submultifidius.3.right, multifidius.submultifidius.4.right, intertransversarius.dorsalis.2.right, intertransversarius.dorsalis.3.right, intertransversarius.dorsalis.4.right, intertransversarius.ventralis.2.right, intertransversarius.ventralis.3.right, intertransversarius.ventralis.4.right
4	0.89312	scapula.right, levator.claviculae.right, levator.scapulae.right, omohyoideus.right, rhomboideus.capitis.right, rhomboideus.right, serratus.ventralis.right, trapezius.right
5	0.00061	clavicle.left, clavicle.right, sternum, hyoid, thyroid, cleidomastoideus.left, cleidomastoideus.right, cleidooccipitalis.left, cleidooccipitalis.right, omohyoideus.left, sternohyoideus.left, sternohyoideus.right, sternomastoideus.left, sternomastoideus.right, sternothyroideus.left, sternothyroideus.right

Table A49 Connectivity modules identified for *Suncus murinus*.

ID	p-value	Elements
1	0.00539	C2, C3, C4, rectus.capitis.dorsalis.major.left, rectus.capitis.dorsalis.major.right, scalenus.b.left, splenius.left, splenius.right, trapezius.anticus.left, trapezius.anticus.right, multifidius.submultifidius.1.left, multifidius.submultifidius.2.left, multifidius.submultifidius.3.left, intertransversarius.dorsalis.1.left, intertransversarius.dorsalis.2.left, intertransversarius.dorsalis.3.left, intertransversarius.ventralis.1.left, intertransversarius.ventralis.2.left, intertransversarius.ventralis.3.left, multifidius.submultifidius.1.right, multifidius.submultifidius.2.right, multifidius.submultifidius.3.right, intertransversarius.dorsalis.1.right, intertransversarius.dorsalis.2.right, intertransversarius.dorsalis.3.right, intertransversarius.ventralis.1.right, intertransversarius.ventralis.2.right, intertransversarius.ventralis.3.right
2	0.95246	ribs.right, iliocostalis.cervicis.left.1, scalenus.a.right, scalenus.b.right
3	0.97033	ribs.left, iliocostalis.cervicis.left, scalenus.a.left
4	0.11436	C1, longus.colli.right, obliquus.capitis.caudalis.left, obliquus.capitis.caudalis.right, obliquus.capitis.cranialis.left, obliquus.capitis.cranialis.right, rectus.capitis.dorsalis.minor.left, rectus.capitis.dorsalis.minor.right, rectus.capitis.ventralis.left, rectus.capitis.ventralis.right
5	5e-05	C5, C6, C7, thoracic.spine, longissimus.capitis.left, longissimus.capitis.right, longissimus.cervicis.left, longissimus.cervicis.right, longus.colli.left, rhomboideus.cervicis.left, rhomboideus.cervicis.right, semispinalis.capitis.left, semispinalis.capitis.right, serratus.ventralis.cervicis.left, serratus.ventralis.cervicis.right, multifidius.submultifidius.4.left, multifidius.submultifidius.5.left, multifidius.submultifidius.6.left, intertransversarius.dorsalis.4.left, intertransversarius.dorsalis.5.left, intertransversarius.dorsalis.6.left, intertransversarius.ventralis.4.left, intertransversarius.ventralis.5.left, intertransversarius.ventralis.6.left, multifidius.submultifidius.4.right, multifidius.submultifidius.5.right, multifidius.submultifidius.6.right, intertransversarius.dorsalis.4.right, intertransversarius.dorsalis.5.right, intertransversarius.dorsalis.6.right, intertransversarius.ventralis.4.right, intertransversarius.ventralis.5.right, intertransversarius.ventralis.6.right
6	0	cranium, clavicle.left, clavicle.right, scapula.left, scapula.right, sternum, hyoid, thyroid, cleido.mastoideus.left, cleido.mastoideus.right, cleido.occipitalis.left, cleido.occipitalis.right, levator.claviculae.left, levator.claviculae.right, longus.capitis.left, longus.capitis.right, rhomboideus.capitis.left, rhomboideus.capitis.right, sternohyoideus.left, sternohyoideus.right, sterno.mastoideus.left, sterno.mastoideus.right, sternothyroideus.left, sternothyroideus.right, trapezius.capitis.left, trapezius.capitis.right

Table A50 Connectivity modules identified for *Tachyglossus aculeatus*.

ID	p-value	Elements
1	0.7044	scapula.left, clavicula.left, levator.scapulae.dorsalis.left, levator.scapulae.ventralis.left, trapezius.anterior.left
2	0.3559	C3, C4, C5, intertransversarii.dorsalis.cervicis.left, longissimus.capitis.left, longissimus.capitis.right, longissimus.cervicis.left, longissimus.cervicis.right, obliquus.capitis.caudalis.right, scalenus.left, scalenus.right, serratus.ventralis.cervicis.left, serratus.ventralis.cervicis.right, intertransversarius.ventralis.3.left, intertransversarius.ventralis.4.left, intertransversarius.ventralis.3.right, intertransversarius.ventralis.4.right
3	0.00035	sternum, hyoid, thyroid, ribs.left, ribs.right, omohyoid.left, omohyoid.right, sterno.cleido.mastoid.left, sterno.cleido.mastoid.right, sternoglossus.left, sternoglossus.right, sternothyroid.left, sternothyroid.right, tongue
4	0.58505	scapula.right, clavicula.right, levator.scapulae.dorsalis.right, levator.scapulae.ventralis.right, trapezius.anterior.right
5	0.15295	C6, C7, thoracic.spine, complexus.major.left, complexus.major.right, iliocostalis.cervicis.left, iliocostalis.cervicis.right, longus.colli.left, longus.colli.right, rhomboideus.left, rhomboideus.right, spinalis.cervicis.left, spinalis.cervicis.right, splenius.left, splenius.right, intertransversarius.ventralis.5.left, intertransversarius.ventralis.6.left, intertransversarius.ventralis.7.left, intertransversarius.ventralis.5.right, intertransversarius.ventralis.6.right, intertransversarius.ventralis.7.right
6	0.00175	cranium, C1, C2, biventer.cervicis.left, biventer.cervicis.right, intertransversarii.dorsalis.cervicis.right, longus.capitis.left, longus.capitis.right, obliquus.capitis.caudalis.left, rectus.capitis.dorsalis.major.left, rectus.capitis.dorsalis.major.right, rectus.capitis.dorsalis.minor.left, rectus.capitis.dorsalis.minor.right, rectus.capitis.lateralis.brevis.left, rectus.capitis.lateralis.brevis.right, rectus.capitis.lateralis.longus.left, rectus.capitis.lateralis.longus.right, rectus.capitis.ventralis.left, rectus.capitis.ventralis.right, intertransversarius.ventralis.1.left, intertransversarius.ventralis.2.left, intertransversarius.ventralis.1.right, intertransversarius.ventralis.2.right

Table A51 Connectivity modules identified for *Tapirus indicus*.

ID	p-value	Elements
1	0.00021	mandible, scapula.right, sternum, hyoid, thyroid, ribs.left, ribs.right, omohyoideus.right, scalenus.1st.rib.right, scalenus.medius.left, scalenus.medius.right, scalenus.transcostalis.left, scalenus.transcostalis.right, sternohyoideus.left, sternohyoideus.right, sternomandibularis.left, sternomandibularis.right, sternomastoideus.left, sternomastoideus.right, sternothyroideus.left, sternothyroideus.right
2	0.0018	C5, C6, C7, levator.scapulae.left, levator.scapulae.right, longissimus.cervicis.left, longissimus.cervicis.right, longus.capitis.left, longus.capitis.right, scalenus.1st.rib.left, semispinalis.capitis.b.left, semispinalis.capitis.b.right, spinalis.cervicis.left, spinalis.cervicis.right, interspinalis.4, interspinalis.5, multifidius.submultifidius.4.left, multifidius.submultifidius.5.left, multifidius.submultifidius.6.left, intertransversarius.dorsalis.4.left, intertransversarius.dorsalis.5.left, intertransversarius.ventralis.4.left, intertransversarius.ventralis.5.left, multifidius.submultifidius.4.right, multifidius.submultifidius.5.right, multifidius.submultifidius.6.right, intertransversarius.dorsalis.4.right, intertransversarius.dorsalis.5.right, intertransversarius.ventralis.4.right, intertransversarius.ventralis.5.right
3	0.01125	thoracic.spine, nuchal.ligament, rhomboideus.cervicis.left, rhomboideus.cervicis.right, semispinalis.capitis.a.left, semispinalis.capitis.a.right, splenius.left, splenius.right, trapezius.left, trapezius.right, interspinalis.6, intertransversarius.dorsalis.6.left, intertransversarius.ventralis.6.left, intertransversarius.dorsalis.6.right, intertransversarius.ventralis.6.right
4	0.00821	C2, C3, C4, iliocostalis.cervicis.left, iliocostalis.cervicis.right, longus.colli.left, longus.colli.right, interspinalis.1, interspinalis.2, interspinalis.3, multifidius.submultifidius.1.left, multifidius.submultifidius.2.left, multifidius.submultifidius.3.left, intertransversarius.dorsalis.2.left, intertransversarius.dorsalis.3.left, intertransversarius.ventralis.2.left, intertransversarius.ventralis.3.left, multifidius.submultifidius.1.right, multifidius.submultifidius.2.right, multifidius.submultifidius.3.right, intertransversarius.dorsalis.2.right, intertransversarius.dorsalis.3.right, intertransversarius.ventralis.2.right, intertransversarius.ventralis.3.right
5	0.00272	cranium, C1, humerus.right, brachiocephalicus.a.right, brachiocephalicus.b.right, longissimus.capitis.left, longissimus.capitis.right, obliquus.capitis.caudalis.left, obliquus.capitis.caudalis.right, obliquus.capitis.cranialis.left, obliquus.capitis.cranialis.right, rectus.capitis.dorsalis.major.left, rectus.capitis.dorsalis.major.right, rectus.capitis.dorsalis.minor.left, rectus.capitis.dorsalis.minor.right, rectus.capitis.lateralis.left, rectus.capitis.lateralis.right, rectus.capitis.ventralis.left, rectus.capitis.ventralis.right
6	0.41123	scapula.left, humerus.left, brachiocephalicus.a.left, brachiocephalicus.b.left, omohyoideus.left

Table A52 Connectivity modules identified for *Trichosurus vulpecula*.

ID	p-value	Elements
1	0.01524	mandible, sternum, thyroid, ribs.left, ribs.right, iliocostalis.dorsi.left, iliocostalis.dorsi.right, sternohyoideus.left, sternohyoideus.right, sternomastoideus.left, sternomastoideus.right, sternothyroideus.left, sternothyroideus.right
2	0	cranium, C1, clavicle.left, clavicle.right, scapula.left, scapula.right, hyoid, atlantoscapularis..ventralis.right, atlantoscapularis.dorsalis.left, atlantoscapularis.dorsalis.right, atlantoscapularis.ventralis.left, biventer.cervicis.left, biventer.cervicis.right, cleidomastoideus.left, cleidomastoideus.right, cleidooccipitalis.left, cleidooccipitalis.right, longissimus.capitis.left, longissimus.capitis.right, obliquus.capitis.cranialis.left, obliquus.capitis.cranialis.right, omohyoideus.left, omohyoideus.right, rectus.capitis.dorsalis.major.left, rectus.capitis.dorsalis.major.right, rectus.capitis.dorsalis.minor.left, rectus.capitis.dorsalis.minor.right, rectus.capitis.lateralis.left, rectus.capitis.lateralis.right, rhomboideus.right, splenius.right
3	0.00022	C5, C6, C7, thoracic.spine, complexus.left, complexus.right, lo.cer..iliocostalis.cervicis..left, lo.cer..iliocostalis.cervicis..right, longus.colli.left, longus.colli.right, scalenus.medius.left, scalenus.medius.right, serratus.magnus.left, serratus.magnus.right, trapezius.left, trapezius.right, multifidius.submultifidius.4.left, multifidius.submultifidius.5.left, multifidius.submultifidius.6.left, intertransversarii.dorsalis.4.left, intertransversarii.dorsalis.5.left, intertransversarii.dorsalis.6.left, intertransversarii.ventralis.4.left, intertransversarii.ventralis.5.left, intertransversarii.ventralis.6.left, spinalis.cervicis.left, multifidius.submultifidius.4.right, multifidius.submultifidius.5.right, multifidius.submultifidius.6.right, intertransversarii.dorsalis.4.right, intertransversarii.dorsalis.5.right, intertransversarii.dorsalis.6.right, intertransversarii.ventralis.4.right, intertransversarii.ventralis.5.right, intertransversarii.ventralis.6.right, spinalis.cervicis.right
4	0.00392	C2, C3, C4, longus.capitis.left, longus.capitis.right, obliquus.capitis.caudalis.left, obliquus.capitis.caudalis.right, rhomboideus.left, scalenus.posticus.left, scalenus.posticus.right, splenius.left, multifidius.submultifidius.1.left, multifidius.submultifidius.2.left, multifidius.submultifidius.3.left, intertransversarii.dorsalis.1.left, intertransversarii.dorsalis.2.left, intertransversarii.dorsalis.3.left, intertransversarii.ventralis.1.left, intertransversarii.ventralis.2.left, intertransversarii.ventralis.3.left, multifidius.submultifidius.1.right, multifidius.submultifidius.2.right, multifidius.submultifidius.3.right, intertransversarii.dorsalis.1.right, intertransversarii.dorsalis.2.right, intertransversarii.dorsalis.3.right, intertransversarii.ventralis.1.right, intertransversarii.ventralis.2.right, intertransversarii.ventralis.3.right

Table A53 Connectivity modules identified for *Vespertilio murinus*.

ID	p-value	Elements
1	4e-05	clavicle.left, clavicle.right, scapula.left, scapula.right, sternum, hyoid, thyroid, cervico.clavicularis.left, cervico.clavicularis.right, cleidomastoideus.left, cleidomastoideus.right, omohyoideus.left, omohyoideus.right, sternohyoideus.left, sternohyoideus.right, sternomastoideus.left, sternomastoideus.right, sternothyroideus.left, sternothyroideus.right
2	0.01239	C2, C3, C4, longus.capitis.left, longus.capitis.right, longus.colli.left, longus.colli.right, splenius.capitis.left, splenius.capitis.right, multifidius.submultifidius.1.left, multifidius.submultifidius.2.left, multifidius.submultifidius.3.left, intertransversarius.dorsalis.1.left, intertransversarius.dorsalis.2.left, intertransversarius.dorsalis.3.left, intertransversarius.ventralis.1.left, intertransversarius.ventralis.2.left, intertransversarius.ventralis.3.left, multifidius.submultifidius.1.right, multifidius.submultifidius.2.right, multifidius.submultifidius.3.right, intertransversarius.dorsalis.1.right, intertransversarius.dorsalis.2.right, intertransversarius.dorsalis.3.right, intertransversarius.ventralis.1.right, intertransversarius.ventralis.2.right, intertransversarius.ventralis.3.right
3	0	C5, C6, C7, thoracic.spine, ribs.left, ribs.right, levator.scapulae.left, levator.scapulae.right, scalenus.medius.left, scalenus.medius.right, scalenus.posterior.left, scalenus.posterior.right, semispinalis.capitis.left, semispinalis.capitis.right, semispinalis.cervicis.left, semispinalis.cervicis.right, multifidius.submultifidius.4.left, multifidius.submultifidius.5.left, multifidius.submultifidius.6.left, intertransversarius.dorsalis.4.left, intertransversarius.dorsalis.5.left, intertransversarius.dorsalis.6.left, intertransversarius.ventralis.4.left, intertransversarius.ventralis.5.left, intertransversarius.ventralis.6.left, multifidius.submultifidius.4.right, multifidius.submultifidius.5.right, multifidius.submultifidius.6.right, intertransversarius.dorsalis.4.right, intertransversarius.dorsalis.5.right, intertransversarius.dorsalis.6.right, intertransversarius.ventralis.4.right, intertransversarius.ventralis.5.right, intertransversarius.ventralis.6.right
4	0.00071	cranium, C1, longissimus.capitis.left, longissimus.capitis.right, obliquus.capitis.caudalis.left, obliquus.capitis.cranialis.left, obliquus.capitis.caudalis.right, obliquus.capitis.cranialis.right, rectus.capitis.dorsalis.major.left, rectus.capitis.dorsalis.major.right, rectus.capitis.dorsalis.minor.left, rectus.capitis.dorsalis.minor.right, rectus.capitis.lateralis.left, rectus.capitis.lateralis.right, rectus.capitis.ventralis.left, rectus.capitis.ventralis.right

Table A54 Connectivity modules identified for *Zalophus californianus*.

ID	p-value	Elements
1	0.00749	sternum, hyoid, thyroid, ribs.left, omohyoideus.left, omohyoideus.right, scalenus.dorsalis.left, scalenus.medius.left, sternomastoideus.left, sternomastoideus.right, sternothyrohyoideus.left, sternothyrohyoideus.right
2	0.8807	ribs.right, scalenus.dorsalis.right, scalenus.medius.right
3	0.03134	ligamentum.nuchae, scapula.left, scapula.right, humerus.left, humerus.right, cleidocervicalis.left, cleidocervicalis.right, cleidomastoideus.left, cleidomastoideus.right, humerotrapezius.left, humerotrapezius.right, levator.scapula.1.left, levator.scapula.1.right, levator.scapula.2.left, levator.scapula.2.right, rhomboideus.capitis.left, rhomboideus.capitis.right, rhomboideus.cervicis.left, rhomboideus.cervicis.right, splenius.left, splenius.right
4	2e-05	C2, C3, C4, complexus.left, complexus.right, interspinalis.1, interspinalis.2, interspinalis.3, intertransversarii.dorsalis.2.left, intertransversarii.dorsalis.2.right, intertransversarii.dorsalis.3.left, intertransversarii.dorsalis.3.right, intertransversarii.dorsalis.4.left, intertransversarii.dorsalis.4.right, intertransversarii.ventralis.left, intertransversarii.ventralis.right, intertransversarius.intermedius.1.left, intertransversarius.intermedius.1.right, intertransversarius.intermedius.2.left, intertransversarius.intermedius.2.right, intertransversarius.intermedius.3.left, intertransversarius.intermedius.3.right, levator.scapula.3.left, levator.scapula.3.right, longus.capitis.right, multifidius.submultifidius.1.left, multifidius.submultifidius.1.right, multifidius.submultifidius.2.left, multifidius.submultifidius.2.right, multifidius.submultifidius.3.left, multifidius.submultifidius.3.right, scalenus.ventralis.left, scalenus.ventralis.right
5	0.00594	cranium, C1, intertransversarii.dorsalis.1.left, intertransversarii.dorsalis.1.right, longus.capitis.left, obliquus.capitis.caudalis.left, obliquus.capitis.caudalis.right, obliquus.capitis.cranialis.left, obliquus.capitis.cranialis.right, rectus.capitis.dorsalis.major.left, rectus.capitis.dorsalis.major.right, rectus.capitis.dorsalis.minor.left, rectus.capitis.dorsalis.minor.right, rectus.capitis.lateralis.left, rectus.capitis.lateralis.right, rectus.capitis.ventralis.left, rectus.capitis.ventralis.right
6	0	C5, C6, C7, thoracic.spine, interspinalis.4, interspinalis.5, intertransversarii.dorsalis.5.left, intertransversarii.dorsalis.5.right, intertransversarii.dorsalis.6.left, intertransversarii.dorsalis.6.right, intertransversarii.dorsalis.7.left, intertransversarii.dorsalis.7.right, intertransversarius.intermedius.4.left, intertransversarius.intermedius.4.right, longissimus.capitis.left, longissimus.capitis.right, longissimus.cervicis.left, longissimus.cervicis.right, longus.colli.left, longus.colli.right, multifidius.submultifidius.4.left, multifidius.submultifidius.4.right, multifidius.submultifidius.5.left, multifidius.submultifidius.5.right, semispinalis.capitis.left, semispinalis.capitis.right, serratus.ventralis.cervicis.left, serratus.ventralis.cervicis.right, spinalis.cervicis.left, spinalis.cervicis.right, splenius.cervicis.left, splenius.cervicis.right
