# Supplementary material for: Musculoskeletal networks reveal topological disparity in mammalian neck evolution
Source: BMC Evol Biol. 2017 Dec 13;17:251. doi: 10.1186/s12862-017-1101-1 (PMC5729486; doi:10.1186/s12862-017-1101-1)
Supplement: Supplementary file 2 — AF2 Systematics and references of investigated species. (PDF 414 kb) [file 12862_2017_1101_MOESM2_ESM.pdf]

Musculoskeletal networks reveal topological disparity in mammalian neck evolution

Patrick Arnold[*], Borja Esteve-Altava, Martin S. Fischer

Additional file AF2_references

*author of correspondence: patrick_arnold@eva.mpg.de

Table A55 Systematics and references of investigated species

Higher Taxon	Order	Family	Species	Predatory behavior	References
Afrotheria	Afrosoricida	Chrysochloridae	*Chrysospalax trevelyani*	P	[1-4]
		Potamogalidae	*Micropotamogale ruwenzorii*	P	[4-7]
	Hyracoidea	Procaviidae	*Procavia capensis*	N	[7-10]
	Proboscidea	Elephantidae	*Elephas maximus*	N	[11-13]
	Sirenia	Dugongidae	*Dugong dugon*	N	[14, 15]
	Tubulidentata	Orycteropodidae	*Orycteropus afer*	N	[7, 16-20]
Euarchonta	Lagomorpha	Leporidae	*Oryctolagus cuniculus*	N	[21-24]
	Primates	Cercopithecidae	*Macaca mulatta*	N	[25-28]
		Hominidae	*Homo sapiens*	N	[29, 30]
		Lorisidae	*Loris tardigradus*	N	[31-34]
	Rodentia	Chinchillidae	*Chinchilla lanigera*	N	[35-38]
		Cricetidae	*Neotoma fuscipes*	N	[22, 39, 40]
		Heteromyidae	*Heteromys desmarestianus*	N	[22, 41]
		Pedetidae	*Pedetes capensis*	N	[42]
		Sciuridae	*Sciurus vulgaris*	N	[21, 36, 43, 44]
	Scandentia	Ptilocercidae	*Ptilocercus lowii*	P	[45-48]
Laurasiatheria	Carnivora	Canidae	*Canis lupus*	P	[49-51]
		Felidae	*Felis silvestris*	P	[52-54]
		Mustelidae	*Galictis cuja*	P	[55, 56]
		Otariidae	*Zalophus californianus*	P	[57, 58]
		Viverridae	*Civettictis civetta*	P	[51, 59-62]
	Cetartiodactyla	Bovidae	*Bos taurus*	N	[63-66]
		Camelidae	*Camelus bactrianus*	N	[9, 67]
		Giraffidae	*Giraffa camelopardalis*	N	[68-72]
		Kogiidae	*Kogia breviceps*	P	[73-77]
		Suidae	*Babyrousa babyrussa*	N	[78-80]
	Chiroptera	Pteropodidae	*Pteropus vampyrus*	N	[81, 82]
		Vespertilionidae	*Vespertilio murinus*	P	[81, 83]
	Eulipotyphla	Erinaceidae	*Erinaceus europaeus*	P	[4, 84, 85]
		Soricidae	*Suncus murinus*	P	[4, 84, 86-8[illegible]
		Talpidae	*Scalopus aquaticus*	P	[4, 89-92]
	Perissodactyla	Equidae	*Equus caballus*	N	[93-95]
		Tapiridae	*Tapirus indicus*	N	[9, 10, 96]
	Pholidota	Manidae	*Manis pentadactyla*	N	[20, 97-99]
Marsupialia	Dasyuromorpha	Dasyuridae	*Sarcophilus harrisii*	P	[100-104]
	Didelphimorphia	Didelphidae	*Didelphis virginiana*	P	[105-108]
	Diprotodontia	Macropodidae	*Macropus rufus*	N	[109-112]
		Phalangeridae	*Trichosurus vulpecula*	N	[113-115]
		Phascolarctidae	*Phascolarctos cinereus*	N	[114-118]
	Notoryctemorphia	Notoryctidae	*Notoryctes typhlops*	P	[119-121]
	Paucituberculata	Caenolestidae	*Caenolestes fuliginosus*	P	[122]
	Peramelemorphia	Thylacomyidae	*Macrotis lagotis*	P	[123-125]

Higher Taxon	Order	Family	Species	Predatory behavior	References
Monotremata	Monotremata	Ornithorhynchidae	*Ornithorhynchus anatinus*	P	[108, 126-131]
		Tachyglossidae	*Tachyglossus aculeatus*	N	[127-129, 132-134]
Xenarthra	Cingulata	Dasypodidae	*Dasypus novemcinctus*	P	[20, 135-13[illegible]
	Pilosa	Bradypodidae	*Bradypus tridactylus*	N	[20, 98, 139-141]
		Cyclopedidae	*Cyclopes didactylus*	N	[20, 98, 136, 142]
		Megalonychidae	*Choloepus didactylus*	N	[20, 98, 141, 143, 144]

Footnote: references also includes those ones for closely related species for which anatomical information were available to asses topological variation within the lineage
P: predatory; N: non-predatory

References

1. Campbell B: A reconsideration of the shoulder musculature of the Cape golden mole. *Journal of Mammalogy* 1938, 19(2):234-240.
2. Parsons F: On the muscles and joints of the giant golden mole (*Chrysochloris trevelyani*). *Proceedings of the Zoological Society of London* 1901, 1:26-34.
3. Puttick JU, Gillian M: The functional anatomy of the neck and forelimbs of the Cape golden mole, *Chrysochloris asiatica* (Lipotyphla: Chrysochloridae). *African Zoology* 1977, 12(2):445-458.
4. Dobson GE: A monograph of the Insectivora, systematic and anatomical. London: J. van Voorst; 1882-1890.
5. Jullien R: Musculature du membre antérieur chez les principaux types d'insectivores. *Memoires du Museum National d'Histoire Naturelle, Serie A, Zoology* 1967, 48(1):1-68.
6. Verheyen WN: Recherches anatomique sur *Micropotamogale ruwenzorii* - 2. La myologie de la tête, du cou et de la patte anterieure. *Bulletin de la Société royale de zoologie d'Anvers* 1961, 21:1-27.
7. Voegele GM: Forelimb myology and the evolutionary relationships of the aardvark, *Orycteropus afer*, and other small afrotheres. *PhD thesis.* Baltimore: John Hopkins University; 2014.
8. Murie J, Mivart GJ: On the myology of *Hyrax capensis*. *Proceedings of the Zoological Society of London* 1865, 33(1):329-352.
9. Windle B, Parson F: The muscles of the Ungulata. Part I - Muscles of the head, neck, and forelimb. *Proceedings of the Zoological Society of London* 1901, 1901:656-702.
10. Beddard FE: Contributions to the anatomy of certain ungulata, including *Tapirus*, *Hyrax*, and *Antilocapra*. In: *Proceedings of the Zoological Society of London.* vol. XI: Wiley Online Library; 1909: 160-197.
11. Miall LC, Greenwood F: The anatomy of the Indian elephant. *Journal of Anatomy and Physiology* 1878, 13:17-50.
12. Shindo T, Mori M: Musculature of Indian elephant. Part III. Musculature of the trunk, neck and head. *Okajimas Folia Anatomica Japonica* 1956, 29(1-2):17-40.
13. Shindo T, Mori M: Musculature of Indian elephant. Part 1. Musculature of the forelimb. *Okajimas Folia Anatomica Japonica* 1956, 28(1-6):89-113.
14. Domning DP: Observations on the Myology of *Dugong dugon* (Müller). *Smithonian Contributions to Zoology* 1977, 226:55pp.
15. Slijper EJ: Comparative biological-anatomical Investigations of the vertebral column and spinal musculature. *Koninklijke Nederlandse Akademie van Wetenschappen, Verhandelingen (Tweede Sectie)* 1946, 42:1-128.
16. Galton JC: The myology of the upper and lower extremities of *Orycteropus capensis*. *Transactions of the Linnean Society of London* 1869, 26(3):567-608.
17. Humphry G: On the myology of *Orycteropus capensis* and *Phoca communis*. *Journal of Anatomy and Physiology* 1868, 2:290-332.
18. Sonntag CF: A monograph of *Orycteropus afer*—I. Anatomy except the nervous system, skin, and skeleton. *Proceedings of the Zoological Society of London* 1925, 95(2):331-437.

19. Thewissen J, Badoux D: The descriptive and functional myology of the fore-limb of the aardvark (*Orycteropus afer*, Pallas 1766). *Anatomischer Anzeiger* 1986, 162:109-123.
20. Windle BCA, Parsons FG: On the myology of the Edentata. *Proceedings of the Zoological Society of London* 1899, 1899:314-339.
21. Alezais H: Contribution à la myologie des rongeurs. Paris: F. Alcan; 1900.
22. Parsons F: Myology of rodents, Part II. An account of the myology of the Myomorpha, together with a comparison of the muscles of the various suborders of rodents. *Proceedings of the Zoological Society of London* 1896, 20:159-192.
23. Krause W: Die Anatomie des Kaninchens in topographischer und operativer Rücksicht, 2nd edition. Leipzig: Verlag von Wilhelm Engelmann; 1884.
24. Craigie EH: Bensley's Practical Anatomy of the Rabbit: An Elementary Laboratory Text-Book in Mammalian Anatomy (Revised and Edited), 8th edition. Philadelphia: The Blakiston Company; 1948.
25. Hartman CG, Strauss WL: The anatomy of the rhesus monkey (*Macaca mulatta*). Baltimore: The Williams & Wilkins Company; 1933.
26. Hsiao C-H: The deep lateral muscles of the neck in *Macaca cyclopis* (Mm. Scalenii). *Okajimas Folia Anatomica Japonica* 1976, 52(5):233-247.
27. Kang W-B: The superficial lateral muscles of the neck in *Macaca cyclopis* (Formosan monkey). *Okajimas Folia Anatomica Japonica* 1975, 52(4):151-165.
28. Richmond FJ, Singh K, Corneil BD: Neck muscles in the rhesus monkey. I. Muscle morphometry and histochemistry. *Journal of Neurophysiology* 2001, 86(4):1717-1728.
29. Williams PL: Gray’s Anatomy: The anatomical basis of medicine and surgery, 38th edition. New York: Churchill Livingstone; 1995.
30. Eisler P: Die Muskeln des Stammes. Jena: Gustav Fischer Verlag; 1912.
31. Diogo R, Wood B: Soft-tissue anatomy of the primates: phylogenetic analyses based on the muscles of the head, neck, pectoral region and upper limb, with notes on the evolution of these muscles. *Journal of Anatomy* 2011, 219(3):273-359.
32. Mivart SGJ, Murie J: Observations on the anatomy of *Nycticebus tardigradus*. *Proceedings of the Zoological Society of London* 1865, 1865:240-256.
33. Murie J, Mivart GJ: On the anatomy of the Lemuroidea. *The Transactions of the Zoological Society of London* 1869, 7(1):1-113.
34. Miller RA: Functional and morphological adaptations in the forelimbs of the slow lemurs. *Developmental Dynamics* 1943, 73(2):153-183.
35. Olborth H: Zur Anatomie des Bewegungsapparates des Chinchilla (postkranialer Bereich). *Anatomischer Anzeiger* 1964, 114:302-327.
36. Parsons FG: On the myology of the sciuromorphine and hystricomorphine rodents. *Proceedings of the Zoological Society of London* 1894, 18:251-297.
37. Wood AE, White RR: The myology of the chinchilla. *Journal of Morphology* 1950, 86(3):547-597.
38. Woods CA: Comparative myology of jaw, hyoid, and pectoral appendicular regions of New and Old World hystricomorph rodents. *Bulletin of the American Museum of Natural History* 1972, 147(3):115-198.
39. Howell AB: Anatomy of the wood rat: comparative anatomy of the subgenera of the American wood rat (genus *Neotoma*). Baltimore: The Williams & Wilkins Company; 1926.
40. Rinker GC: The comparative myology of the mammalian genera *Sigmodon*, *Oryzomys*, *Neotoma*, and *Peromyscus* (Cricetinae), with remarks on their intergeneric relationships. *Miscellaneous Publications, Museum of Zoology, University of Michigan* 1954, 83:1-124.
41. Ryan JM: Comparative myology and phylogenetic systematics of the Heteromyidae (Mammalia, Rodentia). *Miscellaneous Publications, Museum of Zoology, University of Michigan* 1989, 176:1-103.
42. Parsons FG: On the anatomy of the African jumping-hare *(Pedetes caffer*) compared with that of the Dipodidœ. *Journal of Zoology* 1898, 66(4):858-890.
43. Hoffmann CK, Weyenbergh Jr H: Die Osteologie und Myologie von *Sciurus vulgaris* L., verglichen mit der Anatomie der Lemuriden und des *Chiromys* und über die Stellung des letzteren im natürlichen Systeme. Haarlem: Loosjes Erben; 1870.
44. Thorington Jr RW, Darrow K, Betts AD: Comparative myology of the forelimb of squirrels (Sciuridae). *Journal of Morphology* 1997, 234(2):155-182.
45. Clark W: The myology of the tree-shrew (*Tupaia minor*). *Journal of Zoology* 1924, 94(2):461-497.
46. George RM: The limb musculature of the Tupaiidae. *Primates* 1977, 18:1-34.
47. Clark W: On the anatomy of the pen-tailed tree-shrew (*Ptilocercus lowii*). *Proceedings of the Zoological Society of London* 1926, 96(4):1179-1309.
48. Panyutina AA, Korzun LP, Kuznetsov AN: Forelimb morphology of tree shrews. In: *Flight of Mammals: From Terrestrial Limbs to Wings.* Edited by Panyutina AA, Korzun LP, Kuznetsov AN. New York: Springer-Verlag; 2015: 1-50.

49. Evans HE, De Lahunta A: Miller's Anatomy of the Dog. St. Louis: Elsevier Saunders; 2013.
50. Sharir A, Milgram J, Shahar R: Structural and functional anatomy of the neck musculature of the dog (*Canis familiaris*). *Journal of Anatomy* 2006, 208(3):331-351.
51. Windle BCA, Parsons FG: On the myology of the terrestrial Carnivora.—Part I. Muscles of the head, neck, and fore-limb. *Proceedings of the Zoological Society of London* 1897, 65(2):370-409.
52. Cuff AR, Sparkes EL, Randau M, Pierce SE, Kitchener AC, Goswami A, Hutchinson JR: The scaling of postcranial muscles in cats (Felidae) I: forelimb, cervical, and thoracic muscles. *Journal of anatomy* 2016, 229(1):128-141.
53. Reighard J, Jennings HS: Anatomy of the Cat. New York: Henry Holt and Co; 1923.
54. Richmond F, Abrahams V: Morphology and enzyme histochemistry of dorsal muscles of the cat neck. *Journal of Neurophysiology* 1975, 38(6):1312-1321.
55. Ercoli MD, Álvarez A, Busker F, Morales MM, Julik E, Smith HF, Adrian B, Barton M, Bhagavatula K, Poole M: Myology of the head, neck, and thoracic region of the lesser Grison (*Galictis cuja*) in comparison with the red panda (*Ailurus fulgens*) and other carnivorans: phylogenetic and functional implications. *Journal of Mammalian Evolution* 2016:1-34.
56. Ercoli MD, Álvarez A, Stefanini MI, Busker F, Morales MM: Muscular anatomy of the forelimbs of the lesser grison (*Galictis cuja*), and a functional and phylogenetic overview of Mustelidae and other Caniformia. *Journal of Mammalian Evolution* 2015, 22(1):57-91.
57. Howell AB: Contribution to the comparative anatomy of the eared and earless seals (genera *Zalophus* and *Phoca*). 1928, 73:1-142.
58. Mori M: The skeleton and musculature of *Zalophus*. *Okajimas Folia Anatomica Japonica* 1958, 31(3-4):203-284.
59. Devis C: Notes on the myology of *Viverra civetta*. *Journal of Anatomy and Physiology* 1868, 2(2):207-217.
60. Taylor M: The functional anatomy of the forelimb of some African Viverridae (Carnivora). *Journal of Morphology* 1974, 143(3):307-335.
61. Watson M: On the muscular anatomy of *Proteles* as compared with that of *Hyaena* and *Viverra*. *Proceedings of the Zoological Society of London* 1882, 50(3):579-586.
62. Young AH: Myology of *Viverra civetta*. *Journal of Anatomy and Physiology* 1880, 14(2):166.
63. Budras K-D, Habel RE: Bovine Anatomy, 2nd edition. Hannover: Schlütersche Verlagsgesellschaft; 2011.
64. Smuts M: Mm. intertransversarii eervicis of the ox (*Bos taurus* L.). *Anatomia, Histologia, Embryologia* 1976, 5(2):135-146.
65. Smuts M: Areas of muscular attachment and their correlation with foraminous areas of the cervical vertebrae of the ox (*Bos taurus* L.). *Anatomia, Histologia, Embryologia* 1976, 5(3):253-266.
66. Smuts M, le Roux J: Mm. scaleni of the Ox (*Bos taurus* L). *Anatomia, Histologia, Embryologia* 1975, 4(3):256-264.
67. Lesbre MFX: Recherches anatomiques sur les camélidés, vol. 8. Lyon: Archives du Muséum d'Histoire Naturelle de Lyon; 1903.
68. Cobbold TS: Contributions to the anatomy of the giraffe. *Proceedings of the Zoological Society of London* 1860, 28:99-105.
69. Dagg AI: *Giraffa camelopardalis*. *Mammalian Species* 1971(5):1-8.
70. Endo H, Yamagiwa D, Fujisawa M, Kimura J, Kurohmaru M, Hayashi Y: Modified neck muscular system of the giraffe (*Giraffa camelopardalis*). *Annals of Anatomy-Anatomischer Anzeiger* 1997, 179(5):481-485.
71. Murie J: On the horns, viscera, and muscles of the giraffe; with a record of the post mortem examination of two specimens killed by a fire. *Annals and Magazine of Natural History* 1872, 9(51):177-195.
72. Owen R: Notes on the anatomy of the Nubian giraffe. *The Transactions of the Zoological Society of London* 1839, 2(3):217-243.
73. Schulte HvW, Smith MDF: The external characters, skeletal muscles, and peripheral nerves of *Kogia breviceps* (Blainville). *Bulletin American Museum of Natural History* 1918, 38:7-72.
74. Howell AB: Contribution to the anatomy of the Chinese finless porpoise, *Neomeris phocaenoides*. *Proceedings of the United States National Museum* 1927, 70:1-43.
75. Howell AB: Myology of the narwhal (*Monodon monoceros*). *American Journal of Anatomy* 1930, 46(2):187-215.
76. Howell BA: Aquatic mammals. Their adaptations to life the water. Baltimore: Charles C. Thomas; 1930.
77. Strickler TL: Myology of the shoulder of *Pontoporia blainvillei*, including a review of the literature on shoulder morphology in the cetacea. *American Journal of Anatomy* 1978, 152(3):419-431.
78. Kneepkens A, Macdonald A: Vertebral column, rib and sternal muscles of Sulawesi babirusa (*Babyrousa celebensis*). *Anatomia, Histologia, Embryologia* 2011, 40(2):149-161.
79. Kneepkens AF, Badoux DM, MacDonald AA: Descriptive and comparative myology of the forelimb of the babirusa (*Babyrousa babyrussa* L. 1758). *Anatomia, Histologia, Embryologia* 1989, 18(4):349-365.

80. Davis DD: Notes on the anatomy of the babirusa. *Field Museum of Natural History (Zoological Series)* 1940, 22:363-911.
81. Macalister A: The myology of the Cheiroptera. *Philosophical Transactions of the Royal Society of London* 1872, 162:125-171.
82. Mori M: Muskulatur des *Pteropus edulis*. *Okajimas Folia Anatomica Japonica* 1960, 36(3-4):253-307.
83. Maisonneuve P: Traité de l'ostéologie et de la myologie du *Vespertilio murinus*, précédé d'un exposé de la classification des chéiroptèi et de considérations sur les moeurs de ces animaux. Paris: O. Doin; 1878.
84. Neveu P, Gasc JP: Lipotyphla limb myology comparison. *Journal of Morphology* 2002, 252(2):183-201.
85. Gupta B: A study on the skeleton and musculature of the neck, the thorax and the abdomen of the Indian hedgehogs. *Zoologica Poloniae* 1962, 12:365-422.
86. Ärnbäck-Christie-Linde A: Der Bau der Soriciden und ihre Beziehungen zu andern Säugetieren. *Gegenbaurs Morphologisches Jahrbuch* 1907, 36:463-514.
87. Reed CA: Locomotion and appendicular anatomy in three soricoid insectivores. *American Midland Naturalist* 1951, 45:513-671.
88. Sharma D: Studies on the anatomy of the indian insectivore, *Suncus murinus*. *Journal of Morphology* 1958, 102(3):427-553.
89. Gaughran GR: A comparative study of the osteology and myology of the cranial and cervical regions of the shrew, *Blarina brevicauda*, and the mole, *Scalopus aquaticus*. *Miscellaneous Publications, Museum of Zoology, University of Michigan* 1954, 80:1-67.
90. Whidden HP: Comparative myology of moles and the phylogeny of the Talpidae (Mammalia, Lipotyphla). *American Museum of Natural History Novitates* 2000, 3294:1-53.
91. Edwards LF: Morphology of the forelimb of the mole (*Scalops aquaticus*, L.) in relation to its fossorial habits. *Ohio Journal of Science* 1937, 37:20-41.
92. Campbell B: The shoulder anatomy of the moles. A study in phylogeny and adaptation. *American Journal of Anatomy* 1939, 64(1):1-39.
93. Budras K-D, Sack WO, Röck S: Anatomy of the horse: An illustrated text, 4th edition. Hannover: Schlütersche Verlagsgesellschaft; 2003.
94. Gellman K, Bertram J, Hermanson J: Morphology, histochemistry, and function of epaxial cervical musculature in the horse (*Equus caballus*). *Journal of Morphology* 2002, 251(2):182-194.
95. Nickel R, Schummer A, Seiferle E, Frewin J, Wilke KH: The anatomy of the domestic animals. Vol. 1.: The locomotor system of the domestic mammals. New York: Springer-Verlag; 1986.
96. Bressou C: La myologie du tapir (*Tapirus indicus* L.). *Mammalia* 1961, 25(3):358-400.
97. Chan L-K: Extrinsic lingual musculature of two pangolins (Pholidota: Manidae). *Journal of Mammalogy* 1995, 76(2):472-480.
98. Humphry G: The myology of the limbs of the Unau, the Ai, the two-toed anteater, and the Pangolin. *Journal of Anatomy and Physiology* 1869, 3:2-78.
99. Kawashima T, Thorington RW, Bohaska PW, Chen YJ, Sato F: Anatomy of shoulder girdle muscle modifications and walking adaptation in the scaly Chinese pangolin (*Manis pentadactyla pentadactyla*: Pholidota) compared with the partially osteoderm-clad armadillos (Dasypodidae). *The Anatomical Record* 2015, 298(7):1217-1236.
100. Macalister A: On the myology of the Wombat (*Phascolomys wombata*) and the Tasmanian Devil (*Sarcophilus ursinus*). *Annals and Magazine of Natural History* 1870, 5(27):153-173.
101. Carlsson A: Über den Bau des *Dasyuroides byrnei* und seine Beziehungen zu den übrigen Dasyuridae. *Acta Zoologica* 1926, 7(2-3):249-275.
102. Jones FW: The study of a generalized marsupial (*Dasycercus cristicauda* Krefft). *The Transactions of the Zoological Society of London* 1949, 26(5):409-501.
103. Cunningham D: Some points in the anatomy of the Thylacine (*Thylacinus cynocephalus*), Cuscus (*Palangista maculata*) and Phascogale (*Phascogale calura*) collected by HMS Challenger, during the years 1873-1876; with an account of the comparative anatomy of the intrinsic muscles and nerves of the mammalian pes. *Report on the Scientific Results of the Voyage of HMS Challenger Zoology* 1882, 5:1-192.
104. MacCormick A: Myology of the limbs of *Dasyurus viverrinus*. *Journal of Anatomy and Physiology* 1886, 21:103-136.
105. Coues E, Wyman J: On the osteology and myology of *Didelphys virginiana*. *Memoirs of the Boston Society of Natural History* 1872, 2:41-154.
106. Jenkins PA, Weijs W: The functional anatomy of the shoulder in the Virginia opossum (*Didelphis virginiana*). *Journal of Zoology* 1979, 188(3):379-410.
107. Stein BR: Comparative limb myology of two opossums, *Didelphis* and *Chironectes*. *Journal of Morphology* 1981, 169(1):113-140.
108. Nishi S: Zur vergleichenden Anatomie der eigentlichen (genuinen) Rückenmuskeln. *Gegenbaurs Morphologisches Jahrbuch* 1916, 50:167-318.

109. Harvey KJ, Warburton N: Forelimb musculature of kangaroos with particular emphasis on the tammar wallaby *Macropus eugenii* (Desmarest, 1817). *Australian Mammalogy* 2010, 32(1):1-9.
110. Windle BC, Parsons F: On the anatomy of *Macropus rufus*. *Journal of Anatomy and Physiology* 1897, 32:119.
111. Jüschke S: Untersuchungen zur funktionellen Anpassung der Rückenmuskulatur und der Wirbelsäule quadrupeder Affen und Känguruhs. *Anatomy and Embryology* 1972, 137(1):47-85.
112. Parsons FG: On the Anatomy of *Petrogale xanthopus*, compared with that of other Kangaroos. *Proceedings of the Zoological Society of London* 1896, 66:683-714.
113. Barbour RA: The musculature and limb plexuses of *Trichosurus vulpecula*. *Australian Journal of Zoology* 1963, 11(4):488-610.
114. Sonntag CF: The comparative anatomy of the koala (*Phascolartos cinereus*) and vulpine phalanger (*Trichosurus vulpecula*). *Proceedings of the Zoological Society of London* 1921, 91(3):547-577.
115. Sonntag CF: On the myology and classification of the wombat, koala, and phalangers. *Proceedings of the Zoological Society of London* 1922, 92(4):863-896.
116. Forbes W: On some Points in the Anatomy of the Koala *Phascolarctos cinereus*. *Proceedings of the Zoological Society of London* 1881, 49(1):180-195.
117. Macalister A: The muscular anatomy of the koala (*Phascolarctos cinereus*). *Proceedings of the Zoological Society of London* 1865, 1865:127-135.
118. Young AH: The muscular anatomy of the koala (*Phascolarctos cinereus*), with additional notes. *Journal of Anatomy and Physiology* 1882, 16(2):217-242.
119. Carlsson A: Zur Anatomie des *Notoryctes typhlops*. *Zoologisches Jahrbuch Abteilung Anatomie und Ontogonie* 1904, 20:81-122.
120. Warburton NM: Functional morphology of marsupial moles (Marsupialia: Notoryctidae). *Verhandlungen des Naturwissenschaftlichen Vereins in Hamburg* 2006, 42:39-149.
121. Wilson JT: On the myology of *Notoryctes typhlops*, with comparative notes. *Transactions of the Royal Society of SouthAustralia* 1894, 18:3-74.
122. Osgood WH, Herrick CJ, Obenchain JB: A monographic study of the American marsupial *Caenolestes*, with a description of the brain of *Caenolestes* by C. Judson Herrick. *Field Museum of Natural History (Zoological Series)* 1921, 14:1-162.
123. Filan SL: Myology of the Head and Neck of the Bandicoot (Marsupialia, Peramelemorphia). *Australian Journal of Zoology* 1990, 38(6):617-634.
124. Parsons F: On the anatomy of the pig-footed bandicoot (*Chœropus castanotis*). *Journal of the Linnean Society of London, Zoology* 1903, 29(188):64-80.
125. Warburton NM, Grégoire L, Jacques S, Flandrin C: Adaptations for digging in the forelimb muscle anatomy of the southern brown bandicoot (*Isoodon obesulus*) and bilby (*Macrotis lagotis*). *Australian Journal of Zoology* 2014, 61(5):402-419.
126. Coues E: On the myology of the *Ornithorhynchus*. *Proceedings of the Essex Institute* 1871, 6:127-173.
127. Gambaryan PP, Kuznetsov AN, Panyutina AA, Gerasimov SV: Shoulder girdle and forelimb myology of extant Monotremata. *Russian Journal of Theriology* 2015, 14(1):1-56.
128. Howell AB: Morphogenesis of the shoulder architecture. Part V. Monotremata. *The Quarterly Review of Biology* 1937, 12(2):191-205.
129. Jouffroy F, Lessertisseur J, Saban R: Particularités musculaires des Monotrémes – musculature post-craniénne. In: *Traité de Zoologie Mamifères, Tome XVI, Fac III* Edited by Grassé P-P. Paris: Masson et Cie editeurs; 1971: 679-836.
130. Manners-Smith T: On some points in the anatomy of *Ornithorhynchus paradoxus*. *Proceedings of the Zoological Society of London* 1894, 96:715-722.
131. Virchow H: Die tiefen Rückenmuskeln des *Ornithorhynchus*. *Gegenbaurs Morphologisches Jahrbuch* 1929, 60:481-559.
132. Fewkes JW: Contributions to the Myology of *Tachyglossa Hystrix*, *Echidna Hystrix*. *Bulletin of the Essex Institute, Salem* 1877, 9:111-137.
133. Mivart SG: On some points in the Anatomy of *Echidna hystrix*. *Transactions of the Linnean Society of London* 1866, 25(3):379-403.
134. Westling C: Anatomische Untersuchungen uber *Echidna*. *Bihang till Koniglika Svenska Vetenskapsakademiens Handlingar* 1889, 15/4(3).
135. Olson R, Womble M, Thomas D, Glenn Z, Butcher M: Functional morphology of the forelimb of the nine-banded armadillo (*Dasypus novemcinctus*): comparative perspectives on the myology of Dasypodidae. *Journal of Mammalian Evolution* 2016, 23(1):49-69.
136. Galton JC: The myology of *Cyclothurus didactylus*. *Annals and Magazine of Natural History* 1869, 4(22):244-264.
137. Macalister A: A monograph of the anatomy of *Chlamydophorus truncatus* (Harlan), with notes on the structure of other species of Edentata. *The Transactions of the Royal Irish Academy* 1875, 25:219-278.
138. Miles SS: The shoulder anatomy of the armadillo. *Journal of Mammalogy* 1941, 22(2):157-169.

139. Macalister A: On the myology of Bradypus tridactylus; with remarks on the general mucular anatomy of the Edentata. *Annals and Magazine of Natural History* 1869, 4(19):51-67.
140. Mackintosh H: On the myology of the genus *Bradypus*. *Proceedings of the Royal Irish Academy* 1870, 1:517-529.
141. Miller RA: Functional adaptations in the forelimb of the sloths. *Journal of Mammalogy* 1935, 16(1):38-51.
142. Reiss KZ: Myology of the feeding apparatus of myrmecophagid anteaters (Xenarthra: Myrmecophagidae). *Journal of Mammalian Evolution* 1997, 4(2):87-117.
143. Lucae JCG: Statik und Mechanik der Quadrupeden an dem Skelett und den Muskeln des Lemur und eines *Choloepus*. *Abhandlungen der Senckenbergischen Naturforschenden Gesellschaft Frankfurt* 1884, 13:1-92.
144. Mackintosh H: On the muscular anatomy of *Choloepus didactylus*. *Proceedings of the Royal Irish Academy* 1875, 1:66-67^69.
