# Supplementary material for: Musculoskeletal networks reveal topological disparity in mammalian neck evolution
Source: BMC Evol Biol. 2017 Dec 13;17:251. doi: 10.1186/s12862-017-1101-1 (PMC5729486; doi:10.1186/s12862-017-1101-1)
Supplement: Supplementary file 3 — AF3 Additional information on methods. (PDF 210 kb) [file 12862_2017_1101_MOESM3_ESM.pdf]

Musculoskeletal networks reveal topological disparity in mammalian neck evolution

Patrick Arnold[*], Borja Esteve-Altava, Martin S. Fischer

Additional file AF3_methods

*author of correspondence: patrick_arnold@eva.mpg.de

Anatomical definitions

Muscles

We defined cervical muscles as muscles that originate from the cervical vertebrae, the skull (cranium or mandible), the nuchal ligament, or the hyoid/thyroid and insert on the (cervical or thoracic) vertebrae, sternum, pectoral girdle (scapulae, claviculae, humeri), or ribs. The definition includes:

- accessory field muscles (trapezius, sterno-cleido-cephalic, and their derivatives)
- dorsoscapular muscles (cervical and capital parts of rhomboid, atlantoscapularis, serratus ventralis, and their derivatives)
- prevertebral muscles (longus capitis and colli)
- long infrahyoid muscles (sternohyoid, sternothyroid, and omohyoid)
- cervical costal muscles (scaleni, intertransversarii ventralis, and their derivatives)
- transversospinal muscles (semispinalis capitis and cervicis, multifidi and their derivatives)
- sacrospinal muscles (cervical and capital parts of the longissimus, intertransversarii dorsalis and medialis, cervical and capital parts of the iliocostalis, and their derivatives)
- spinalis muscles (cervical spinalis and interspinalis)
- spinotransversal muscles (splenius capitis and cervicis)
- suboccipital muscles (rectus capitis dorsalis major, dorsalis minor, intermdius, lateralis, and ventralis and oblique capitis cranialis and caudalis)

The long infrahyoid muscles (sterno-, thyro- and omohyoid) were included as they act synergistic during head/neck motion [1, 2]. They were also closely related to the neck complex in an earlier study [3]. The thyrohoid muscle, in contrast, is excluded here as it is integrated in the laryngeal complex [3]. In cases in which the arrangement of the small intervertebral muscles (interspinalis, intertransversarii, multifidi) are not reported in detail, the descriptions of the large scale comparative works were used [4-6].

Bones and ligaments

Although consisting of several bones, the cranium, thoracic spine, left/right ribs, and hyoid were represented by only one element, respectively, to capture that their internal movements are not part of the head/neck motion. The nuchal ligament was only included if it was present as a distinct structure (e.g., when funicular and lamellar portions are present) rather than only

by a nuchal raphe. Taxa in which the claviculae are either absent, reduced, intra-muscular or without physical connection to the sternum were treated as aclaviculate. Their cleidocephalic and clavo-deltoid muscles form a single functional unit connecting the cranium with the humeri (i.e., cephalo-humeral muscles).

Community structure and modularity

The community structure of an anatomical network is a hypothesis of modularity of their anatomical elements based on their structural relations, which can be caused by or affect to developmental, geometric and functional relations among parts (Esteve-Altava et al. 2013). A network module (or *community*) is a group of nodes highly connected among them and poorly connected to nodes in other groups. Finding the best partition of a medium-large network into modules is an NP-complete problem. For this reason community detection algorithms use heuristic approaches to find the best partition into modules (Fortunato 2010).

We used a spin-glass model and simulated annealing algorithm to identify the modules of the network. In this model, each node can be in one of c partitions, and the links between nodes specify which pairs of nodes would prefer to stay in the same module (because Q is higher) and which ones prefer to have different module (because Q is lower). The model is then simulated for a given number of steps. In the first steps, the algorithm is allowed, with more frequency, to accept partitions that are worse (lower Q) than our current partition. This gives the algorithm the ability to jump out of any local optimums it finds itself in early on in execution. Each successive step, this frequency of accepting worst partitions decreases, allowing the algorithm to gradually focus in on a area of the search space in which hopefully, a close to optimum solution can be found. For each network the algorithm is iterated 100 times and we take the consensus result, i.e., present most frequently.

We measured the *modularity* (Q) as defined by Newman and Girvan (2004) for the resulting modular organization of every method. The expected error on Q has been calculated using a jackknife procedure where every link is an independent observation, as suggested in (Newman and Girvan 2004). According to these authors, if the number of connections within modules is no better than random, Q will be close to 0; the higher the Q the stronger the modular organization of the network (maximum, Q=1); in practice, strongly modular networks show Q vales ranging from 0.3 to 0.7. Therefore, we consider that an anatomical network has a strong

modular structure if $Q - error \geq 0.3$. The expected error on Q can be calculated using a jackknife procedure, where every link is an independent observation.

Complementarily, we evaluated the statistical significance of every module independently using a two-sample Wilcoxon rank-sum test on the *internal* and *external* connections of the module. According to the general definition of module as a group of nodes highly connected among them and poorly connected to nodes in other groups, we expect internal connections to be significatively higher than external connections. Thus,

- Ho: internal degree = external degree
- Ha: internal degree > external degree

A low *p-value* tells us to reject Ho, and hence we can assume the alternative hypothesis that the nodes of the module are more connected among them than to other nodes outside the module. In other words, the module identified is not expected by a random grouping of nodes.

Finally, to compare the degree of modularity among different anatomical networks we calculate the Parcellation index.

References

1. de Mayo T, Miralles R, Barrero D, Bulboa A, Carvajal D, Valenzuela S, Ormeño G. Breathing type and body position effects on sternocleidomastoid and suprahyoid EMG activity. *Journal of Oral Rehabilitation* 2005; 32(7):487-494.
2. Forsberg CM, Hellsing E, Linder-Aronson S, Sheikholeslam A. EMG activity in neck and masticatory muscles in relation to extension and flexion of the head. *European Journal of Orthodontics* 1985; 7(3):177-184.
3. Esteve-Altava B, Diogo R, Smith C, Boughner JC, Rasskin-Gutman D. Anatomical networks reveal the musculoskeletal modularity of the human head. *Scientific Reports* 2015; 5:8298.
4. Eisler P. Die Muskeln des Stammes. Jena: Gustav Fischer Verlag; 1912.
5. Jouffroy FK. Musculature épisomatique. In: *Traité de Zoologie Mamifères, Tome XVI, Fac II.* Edited by Grassé PP. Paris: Masson et Cie editeurs; 1968: 479-548.
6. Lessetisseur J. Musculature hyposomatique. In: *Traité de Zoologie Mamifères, Tome XVI, Fac II.* Edited by Grassé P-P. Paris: Masson et Cie editeurs; 1968: 549-732.
7. Esteve-Altava B, Marugan-Lobon J, Bastir M, Botella H, Rasskin-Gutman D. Grist for Riedl's Mill: A network model perspective on the integration and modularity of the human skull. *Journal of Experimental Zoology Part B: Molecular and Developmental Evolution* 2013; 320(8): 489–500.
8. Fortunato S. Community detection in graphs. *Physics Reports* 2010; 486: 75–174.
9. Newman MEJ, Girvan M. Finding and evaluating community structure in networks. *Phyisical Review E* 2004; 69(2): 026113.
